# K_3_PO_4_-promoted domino reactions: diastereoselective synthesis of *trans*-2,3-dihydrobenzofurans from salicyl *N-tert*-butanesulfinyl imines and sulfur ylides†‡

**DOI:** 10.1039/c9ra00309f

**Published:** 2019-04-16

**Authors:** Minxuan Zhang, Tianyu Lu, Yun Zhao, Guixian Xie, Zhiwei Miao

**Affiliations:** State Key Laboratory and Institute of Elemento-Organic Chemistry, College of Chemistry, Nankai University Weijin Road 94 Tianjin 300071 People's Republic of China miaozhiwei@nankai.edu.cn; Collaborative Innovation Center of Chemical Science and Engineering (Tianjin) Tianjin 300071 People's Republic of China; College of Resources and Environment, Hunan Agricultural University Changsha 410128 People's Republic of China

## Abstract

An efficient domino annulation between sulfur ylides and salicyl *N-tert*-butylsulfinyl imines was developed. The reaction proceeds with a diastereodivergent process, the configuration of the sulfinyl group determining the stereochemical course of the reaction. The method allows the synthesis of a highly substituted *trans*-2,3-dihydrobenzofuran skeleton with high yield and good chemo- and diastereoselectivity.

## Abstract 2

The diastereospecific formation of *trans*-2,3-dihydrobenzofurans has been achieved *via* a domino annulation between sulfur ylides and salicyl *N-tert*-butylsulfinyl imines with a diastereodivergent process.

## Graphical abstract

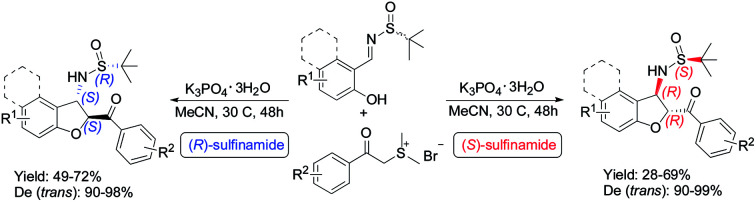

## Introduction

**Fig. 1:**
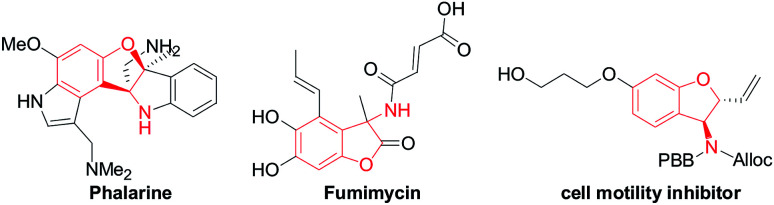
Representative structures containing the 2,3-dihydrobenzofuran motif.

Functionalized chiral benzofuran bearing three carbon stereocenters are the core of many bioactive natural products and pharmaceuticals.^1^ Such cores also offer valuable chiral building blocks for the enantioselective synthesis of biologically active compounds.^2^ Especially, the benzofurans with the amino group at the 3-position are vital in drug discovery and considered important for the bioactivity of these molecules.^3^ For example, phalarine, isolated from *Phalaris coerulescens*, is a type of important natural product, and has exhibited high structural diversity and a broad bioactivity profile.^4^ Fumimycin has demonstrated anti-bacterial properties while the synthetic cell motility inhibitor shows good biological effects in cell-based assays (Fig. 1).^5^

**Scheme 1:**
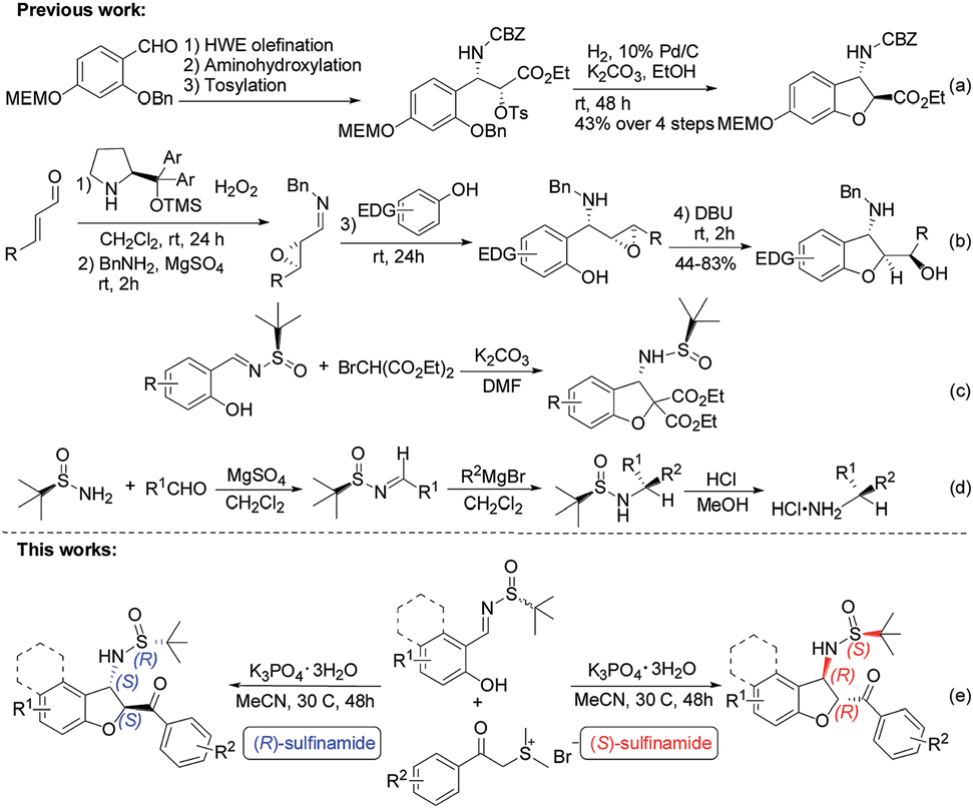
Previous and proposed work.

Diastereoselective synthesis of such significant chiral benzofurans remains a considerable challenge. 3-Substituted 2,3-dihydrobenzofurans have been synthesized by a variety of methods such as radical cyclizations,^6^ electrocyclizations,^7^ anionic cyclizations,^8^ biomimetic couplings and cycloaddition,^9^ Lewis acid promoted reactions,^10^ transition-metal-catalyzed processes,^11^ and so on.^12^ In 2008, Sabourin, Arya and co-workers have developed a practical, enantioselective route to access 3-amino-2,3-dihydrobenzofurans (Scheme 1a).^13^ In 2011, Jørgenson and co-workers developed a highly efficient asymmetric organocatalytic cascade reaction to access 3-amino-2,3-dihydrobenzofurans through four sequential synthetic steps in one pot (Scheme 1b).^14^ In 2015, Zhao and co-workers reported an efficient domino reaction to chiral synthesize different substituted 2,3-dihydrobenzofuran-derived β-amino esters from *ortho*-hydroxyl aromatic *N-tert*-butylsulfinyl imines and diethyl bromomalonate promoted by K_2_CO_3_ (Scheme 1c).^15^ Despite many advances, the development of novel asymmetric catalytic methods to access this class of useful molecules to be highly appealing in modern asymmetric synthesis.

Chiral *N-tert*-butanesulfinyl imines, one class of the most efficient auxiliaries pioneered by Ellman, have been extensively used for the preparation of various chiral amines including variously substituted benzofuran-derived β-amino esters.^16^ Enantiomerically pure aldimines and ketoimines generated from an aldehyde or ketone with an alkyl or aryl sulfinamide are versatile building blocks in the construction of chiral amines, and their application has attracted a large amount of interest.^17^ In 1997, Ellman reported the preparation of *tert*-butanesulfinimines by direct condensation of a sulfinamide with aldehydes in the presence of MgSO_4_. Furthermore, *tert*-butanesulfinimines reacted with Grignard reagents to provide *tert*-butane sulfinamides in high yields with high diastereoselectivity. Removal of the sulfinyl group is achieved with stoichiometric HCl in methanol to afford the desired α-branched amines (Scheme 1d).^18^

In spite of the obvious interesting properties of Ellman's chiral auxiliary such as the availability of both enantiomers and the mild conditions required for its cleavage, we speculated that the chirality of the auxiliary of the imine play an important role in the reaction stereoselectivity. Based on our previous study on the chemistry of chiral auxiliary,^19^ we report the domino annulation of salicyl *N-tert*-butylsulfinyl imines with sulfur ylides, which allows efficient asymmetric synthesis of *trans*-2,3-disubstituted 2,3-dihydrobenzofurans with high chemo- and stereoselectivity (Scheme 1e). The anticipated diastereodivergent process was investigated by combining both enantiomeric sulfinylimine auxiliaries employed.

## Results and discussion

We initiated our investigation by subjecting sulfur ylide precursor 2a (0.50 mmol, 2.5 equiv.)^20^ to salicyl *N-tert*-butylsulfinyl imine 1a (0.20 mmol)^21^ in the presence of K_2_CO_3_ (0.50 mmol, 2.5 equiv.) in CH_3_CN at room temperature. To our delight, the domino reaction proceeded smoothly to provide *trans*-3-(2-methylpropane-2-sulfinamide)-2,3-dihydrobenzofuran 3a in 78% yield and 72 : 28 diastereoselectivity with *trans*-3a being the major diastereomer (Table 1, entry 1). In accordance with the previous reports on base promoted domino reactions, it was found that bases strongly influenced the yield.^22^ When KO^*t*^Bu was used, the reaction became complicated and the product 3a was isolated in low yield (Table 1, entry 2). Similarly, when the reaction was promoted with the weak base KOAc, the reaction rate was very slow and a low yield was obtained after 48 hours (Table 1, entry 3). To our great delight, in the presence of 0.50 mmol K_3_PO_4_·3H_2_O in CH_3_CN at room temperature, after 48 h, the desired product 3a was isolated in 87% yield (Table 1, entry 4). However, when the organic base DABCO (1,4-diazabicyclo [2.2.2] octane) was employed, and the yield of the desired product was higher than those using K_2_CO_3_ and KOAc but still lower than that K_3_PO_4_·3H_2_O (Table 1, entries 5). The base amount was also examined as shown in Table 1. Increasing or decreasing the amount of base resulted in decrease of the efficiency of this reaction (Table 1, entries 6–7).

**Table 1:** Optimization of reaction conditions^a^

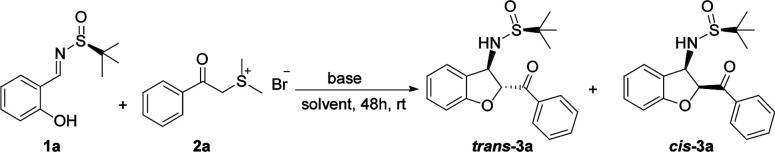				
Entry	Base	Solvent	*trans* : *cis*^b^	Combined yield (%)^c^
1	K_2_CO_3_	CH_3_CN	72 : 28	78
2	KO^*t*^Bu	CH_3_CN	70 : 30	16
3	KOAc	CH_3_CN	73 : 27	34
4	K_3_PO_4_·3H_2_O	CH_3_CN	71 : 29	87
5	DABCO	CH_3_CN	72 : 28	44
6^d^	K_3_PO_4_·3H_2_O	CH_3_CN	71 : 29	46
7^e^	K_3_PO_4_·3H_2_O	CH_3_CN	70 : 30	57
8	K_3_PO_4_·3H_2_O	Toluene	54 : 46	6
9	K_3_PO_4_·3H_2_O	CH_2_Cl_2_	58 : 42	33
10	K_3_PO_4_·3H_2_O	ClCH_2_CH_2_Cl	59 : 41	40
11	K_3_PO_4_·3H_2_O	THF	80 : 20	54
12	K_3_PO_4_·3H_2_O	Acetone	61 : 39	11
13	K_3_PO_4_·3H_2_O	AcOEt	56 : 44	44
14	K_3_PO_4_·3H_2_O	CHCl_3_	76 : 24	4
15^f^	K_3_PO_4_·3H_2_O	CH_3_CN	72 : 28	93

a Unless otherwise specified, all reactions were carried out using *N-tert*-butylsulfinyl imine 1a (0.20 mmol) and sulfur ylide precursor 2a (0.50 mmol, 2.5 equiv.) in 2 mL solvent with 0.50 mmol of base at room temperature.

b Determined by ^1^H NMR (crude reaction mixture).

c Combined yield of isolated products of *trans*-3a and *cis*-3a after column chromatography.

d K_3_PO_4_·3H_2_O loading is 0.10 mmol.

e K_3_PO_4_·3H_2_O loading is 0.40 mmol.

f The reaction temperature is 30 °C.

Subsequently, we investigated the effects of solvent on the reactivity, when K_3_PO_4_·3H_2_O was used as the base. A rather low yield and selectivity was obtained when toluene was used as the solvent (Table 1, entry 8). Among the solvents examined, the use of CH_3_CN gave the best result (Table 1, entries 9–14). Higher reactivity was observed at elevated a temperature of 30 °C, and the product was obtained in 93% yield and 72 : 28 diastereoselectivity (Table 1, entry 15). On the basis of the above experimental results, the optimal reaction conditions for this transformation were determined to be *N-tert*-butylsulfinyl imine 1a (0.20 mmol), sulfur ylide precursor 2a (0.50 mmol), and K_3_PO_4_·3H_2_O (2.5 equiv.) in CH_3_CN as solvent at 30 °C.

Having established the optimal reaction conditions for the synthesis of chiral sulfonamide 2,3-dihydrobenzofuran-2-yl-phenyl methanone, we next focused on the substrate scope of this transformation and the results are shown in Table 2. Representative *N-tert*-butylsulfinyl imines 1 with various substitutions were easily prepared by the condensation of either enantiomer of *tert*-butanesulfinamide with the corresponding aldehydes according to the reported procedure.^21^ In all instances, imines were prepared in high yield and as single enantiomers. Generally, the domino reactions between a range of readily available *ortho*-hydroxyl aromatic *N-tert*-butylsulfinyl imines *S*-1 and sulfur ylide precursor 2a provided *trans*-3-(2-methylpropane-2-sulfinamide)-2,3-dihydrobenzofuran 3 in moderate yields with moderate diastereoselectivities (Table 2, entries 1–5). The electronic property of the aromatic substituent has little effect on the yield and stereoselectivity. Even with a sterically hindered substrate, the reaction proceeded smoothly to give the desired product in moderate yield. We were delighted to find that the *N-tert*-butyl sulfinyl imine of β-naphthylaldehyde *S*-1e, underwent smooth sequential annulation with 2a, to give the corresponding product *trans*-(*Ss*,2*R*,3*R*)-3e in moderate yield with moderate diastereoselective (Table 2, entry 5).

**Table 2:** Scope of the reaction^a^^,^^b^

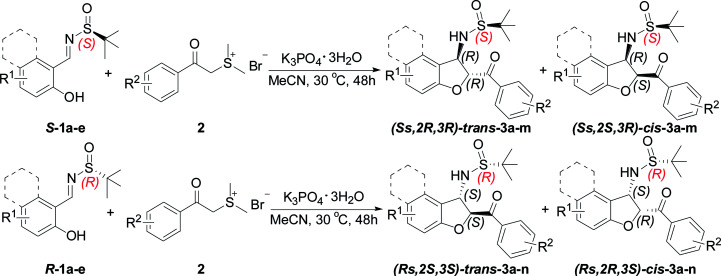						
Entry	Major product	R^1^	R^2^	Yield (%)^c^	*trans* : *cis*^d^	De (*trans*) (%)^e^
1	*trans*-(*Ss*,2*R*,3*R*)-3a	H (*S*-1a)	H (2a)	67	72 : 28	>99
2	*trans*-(*Ss*,2*R*,3*R*)-3b	5-Cl (*S*-1b)	H (2a)	40	64 : 36	98
3	*trans*-(*Ss*,2*R*,3*R*)-3c	5-Br (*S*-1c)	H (2a)	65	71 : 29	90
4	*trans*-(*Ss*,2*R*,3*R*)-3d	3,5-Cl (*S*-1d)	H (2a)	47	63 : 37	98
5^f^	*trans*-(*Ss*,2*R*,3*R*)-3e	H (*S*-1e)	H (2a)	41	72 : 28	98
6	*trans*-(*Ss*,2*R*,3*R*)-3f	5-Cl (*S*-1b)	4-Cl (2b)	54	77 : 23	98
7	*trans*-(*Ss*,2*R*,3*R*)-3g	5-Br (*S*-1c)	4-Cl (2b)	41	77 : 23	98
8	*trans*-(*Ss*,2*R*,3*R*)-3h	3,5-Cl (*S*-1d)	4-Cl (2b)	28	56 : 44	90
9^f^	*trans*-(*Ss*,2*R*,3*R*)-3i	H (*S*-1e)	4-Cl (2b)	57	88 : 12	98
10^f^	*trans*-(*Ss*,2*R*,3*R*)-3j	H (*S*-1e)	4-Me (2c)	53	56 : 44	98
11	*trans*-(*Ss*,2*R*,3*R*)-3k	5-Br (*S*-1c)	4-Me (2c)	53	75 : 25	98
12	*trans*-(*Ss*,2*R*,3*R*)-3l	5-Cl (*S*-1b)	4-Me (2c)	69	70 : 30	98
13	*trans*-(*Ss*,2*R*,3*R*)-3m	5-Br (*S*-1c)	4-NO_2_ (2d)	—	—	—
14	*trans*-(*Rs*,2*S*,3*S*)-3a	H (*R*-1a)	H (2a)	65	72 : 28	90
15	*trans*-(*Rs*,2*S*,3*S*)-3b	5-Cl (*R*-1b)	H (2a)	69	75 : 25	98
16	*trans*-(*Rs*,2*S*,3*S*)-3c	5-Br (*R*-1c)	H (2a)	60	63 : 37	98
17^f^	*trans*-(*Rs*,2*S*,3*S*)-3d	3,5-Cl (*R*-1d)	H (2a)	55	68 : 32	98
18	*trans*-(*Rs*,2*S*,3*S*)-3e	H (*R*-1e)	H (2a)	55	56 : 44	98
19	*trans*-(*Rs*,2*S*,3*S*)-3f	5-Cl (*R*-1b)	4-Cl (2b)	69	74 : 26	98
20	*trans*-(*Rs*,2*S*,3*S*)-3g	5-Br (*R*-1c)	4-Cl (2b)	55	70 : 30	98
21	*trans*-(*Rs*,2*S*,3*S*)-3h	3,5-Cl (*R*-1d)	4-Cl (2b)	51	61 : 39	92
22^f^	*trans*-(*Rs*,2*S*,3*S*)-3i	H (*R*-1e)	4-Cl (2b)	49	63 : 37	90
23^f^	*trans*-(*Rs*,2*S*,3*S*)-3j	H (*R*-1e)	4-Me (2c)	72	73 : 27	98
24	*trans*-(*Rs*,2*S*,3*S*)-3l	5-Cl (*R*-1b)	4-Me (2c)	62	72 : 28	98
25	*trans*-(*Rs*,2*S*,3*S*)-3n	5-Br (*R*-1c)	4-Me (2c)	63	64 : 36	98

a Reaction conditions: *N-tert*-butylsulfinyl imines 1 (0.20 mmol), sulfur ylide 2 (0.50 mmol), in 2 mL of MeCN at 30 °C in the presence of 250 mol % of K_3_PO_4_·3H_2_O.

b The suffix *R* or *S* in the numbering refers to the absolute configuration of the sulfinylimine auxiliary.

c Isolated yield after silica gel chromatography of 3. *trans* and *cis* adducts have been separated by column chromatography and that only the major *trans*-adducts are described.

d *Cis*/*trans* ratio determined from the ^1^H NMR of the crude reaction mixture.

e De determined from ^1^H NMR of the crude reaction mixture.

f *N-tert*-Butyl sulfinyl imine of β-naphthylaldehyde was used.

Further investigations using other sulfur ylide precursors and salicyl *N-tert*-butyl sulfinyl imines were performed under the optimized conditions. A series of sulfur ylide precursor proved to be suitable for this reaction. Aryl units containing electron-donating or electron-withdrawing substituents in sulfur ylides were readily tolerated, thus giving preferentially the corresponding *trans*-3-(2-methylpropane-2-sulfinamide)-2,3-dihydrobenzofuran 3 in moderate yields with moderate diastereoselectivities (Table 2, entries 6–12). Notably, when the substituent was at the 4-position with an electron-withdrawing group of the benzene ring of sulfur ylide precursor 2d, the reaction cannot work and no desired product 3m observed (Table 2, entry 13). In contrast, when the enantiomeric (*R*)-chiral auxiliary (1a–e) was used, the diastereoselectivity was the same as the (*S*)-configured chiral auxiliary (Table 2, entries 14–25).

**Fig. 2:**
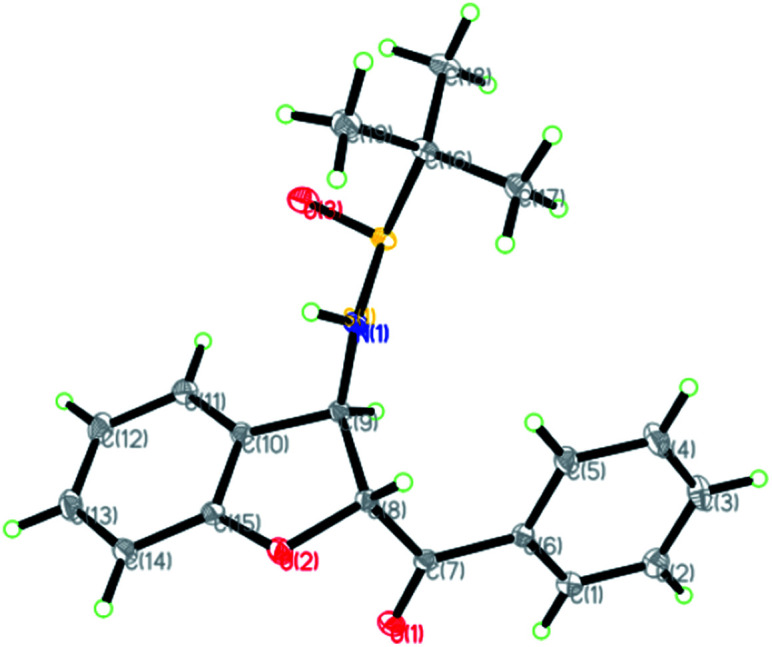
X-ray crystal structure of *trans*-(*Ss*,2*R*,3*R*)-3a.

**Fig. 3:**
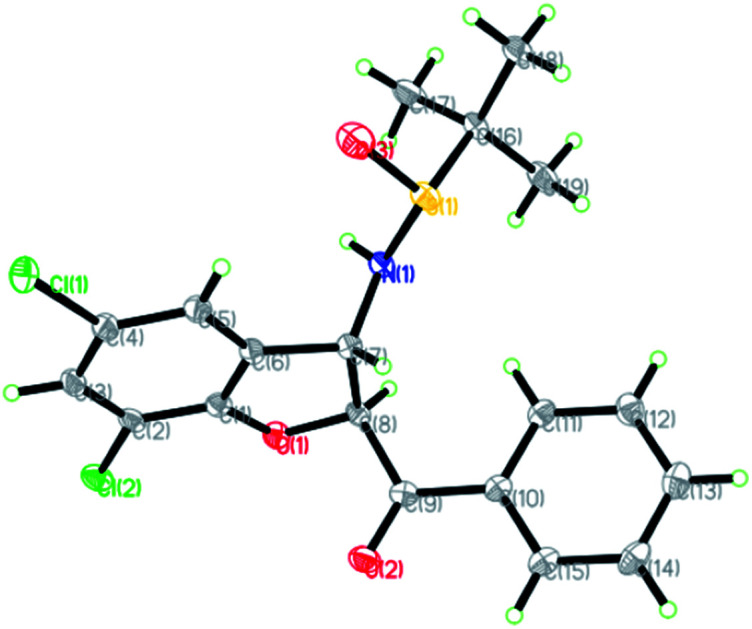
X-ray crystal structure of *trans*-(*Rs*,2*S*,3*S*)-3d.

The relative configuration of the major diastereomers obtained from the reaction of sulfur ylide precursor 2a and salicyl *N-tert*-butylsulfinyl imines *S*-1a and *R*-1d could be determined by X-ray crystallographic analysis (Fig. 2 and 3).^23^ Both products *trans*-(*Ss*,2*R*,3*R*)-3a and *trans*-(*Rs*,2*S*,3*S*)-3d are derived from the same *N-tert*-butyl sulfinylimine enantiomer but with differently configured chiral auxiliaries, and the different configuration of the formed amine stereocenter clearly proved that the diastereoselection was determined by the configuration of the auxiliary. On that basis, the stereochemistry of the other major and minor isomers was assigned.

**Scheme 2:**
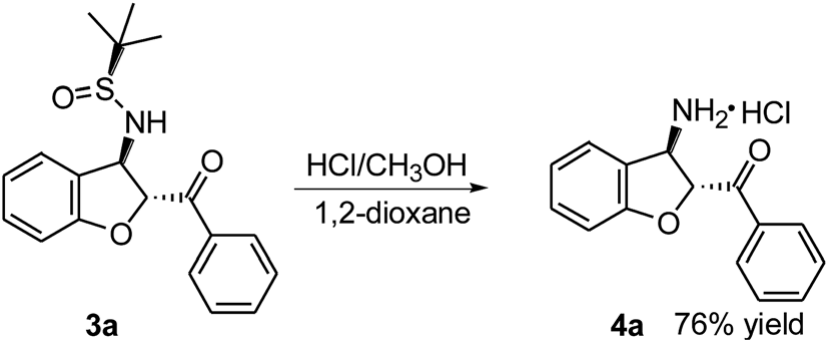
Desulfinylation of *trans*-(*Ss*,2*R*,3*R*)-3a.

To demonstrate further the synthetic utility of these findings, (2*R*,3*R*)-(3-amino-2,3-dihydro-benzofuran-2-yl)-phenyl-methanone 4a was readily synthesized from *trans*-(*Ss*,2*R*,3*R*)-3a (Scheme 2). The deprotection and hydrolysis of 3a in HCl (12 M) at room temperature gave the desired optically active 4a with anti-relative configuration in 76% yield as the hydrochloride salt.

**Scheme 3:**
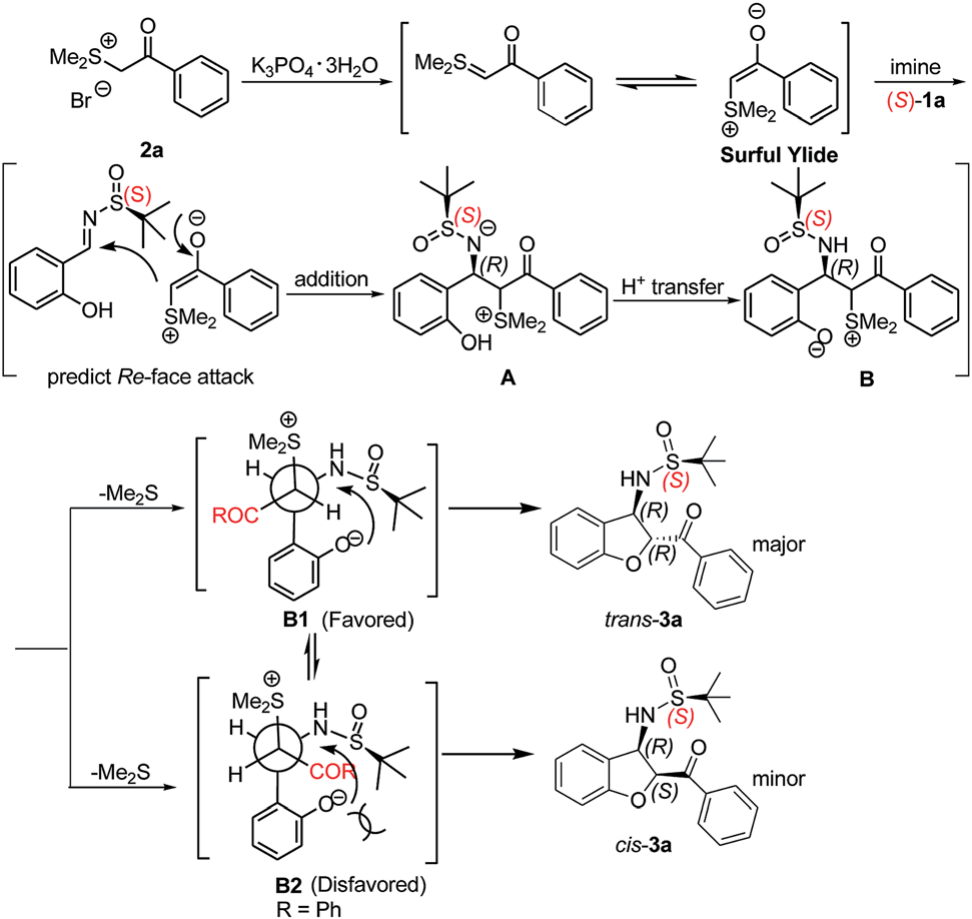
Explanation of stereochemical outcomes.

A mechanism for this domino reaction is proposed on the basis of previous literature reports and is shown in Scheme 3.^24^ The reaction might be initiated by the formation of the surfur ylide *via* the deprotonation of 2a. Subsequent Re-face attack (for an (*S*)-configured sulfinylimine) nucleophilic addition of the surfur ylide to the electron-deficient imine 1a yielded the intermediate A. Intermediate A will transform into intermediate B under proton transfer. As shown in Newman projections B1 and B2, intermediate B1 is the favoured one, followed by S_N_2 substitution to give the *trans*-configured 2,3-disubstituted dihydrobenzofuran 3a.

## Conclusions

In conclusion, we have developed a simple, convenient, and stereoselective domino reaction between salicyl *N-tert*-butylsulfinyl imines and sulfonium salts that provides a new method for the construction of *trans*-2,3-disubstituted 2,3-dihydrobenzofurans in good yields and good chemo- and diastereoselectivities. The domino reaction of the former proceeds with diastereodivergent process, with the configuration of the chiral auxiliary determining the stereo induction. Exploration of the scope and limitations of this reaction and the use of such dihydrobenzofurans to provide concise routes to more complex structures are ongoing and will be reported in due course.

## Experimental

### General methods

All reactions were performed under N_2_ atmosphere in oven-dried glassware with magnetic stirring. Solvents were dried and distilled prior to use according to the standard methods. Unless otherwise indicated, all materials were obtained from commercial sources, and used as purchased without dehydration. Column chromatography was performed on silica gel 200–300 mesh. Nitrogen gas (99.999%) was purchased from Boc Gas Inc. ^1^H and ^13^C NMR spectra were measured at 400 and 101 MHz, respectively. The solvents used for NMR spectroscopy were CDCl_3_ and CD_3_OD, using tetramethylsilane as the internal reference. Chemical shifts were recorded in parts per million (ppm). Coupling constants were given in Hz. The crystal structure was determined on a Bruker SMART 1000 CCD diffractometer. Mass spectra were obtained using an electrospray ionization (ESI-TOF) mass spectrometer. Melting points were determined on a T-4 melting point apparatus (uncorrected).

### Preparation of salicyl *N-tert*-butylsulfinyl imines 1^15^

Under a nitrogen atmosphere, to a mixture of salicylaldehyde (7.2 mmol, 1.2 equiv.) and sulfinamide (6 mmol, 1.0 equiv.) in CH_2_Cl_2_ was added Ti(O*i*-Pr)_4_ (3–5 equiv.). After the mixture was stirred at room temperature for 48 h, 15 mL saturated solution of sodium bicarbonate was added. Stirring for a further 15 min was followed by filtration over a pad of MgSO_4_ and Celite. The filter cake was washed with EtOAc and the filtrate concentrated under reduced pressure. The residue was purified by flash column chromatograph (eluted with 20 : 1 petroleum ether/EtOAc) to afford pure salicyl *N-tert*-butylsulfinyl imine.

### General procedure for synthesis of *trans*-2,3-disubstituted 2,3-dihydrobenzofurans 3

Under a nitrogen atmosphere, to a mixture of sulfur ylide precursor 2 (0.5 mmol, 2.5 equiv.), and K_3_PO_4_·3H_2_O (133 mg, 0.5 mmol, 100 mmol %) was added CH_3_CN (1 mL) *via* a syringe and allowed to stir for 5 min at room temperature. Salicyl *N-tert*-butylsulfinyl imine 1 (0.2 mmol, 1.0 equiv.) in CH_3_CN (1 mL) was added and the reaction was allowed to stir for 48 h at 30 °C. The reaction was monitored by TLC spectroscopy. After the reaction was completed, the reaction mixture was directly purified by flash column chromatograph (eluted with 3 : 1 *n*-heptane/EtOAc) to afford the product 3.

#### *trans*-(*S*)-*N*-(2*R*,3*R*)-2-Benzoyl-2,3-dihydrobenzofuran-3-yl-2-methylpropane-2-sulfinamide (3a)

White solid. Mp: 68–70 °C; [α]^20^_D_ = −78.67 [*c* = 0.95, CHCl_3_]; ^1^H NMR (400 MHz, CDCl_3_) *δ* 8.02 (d, *J* = 7.1 Hz, 2H), 7.57–7.46 (m, 4H), 7.19 (s, 1H), 6.99–6.88 (m, 1H), 6.86 (d, *J* = 7.9 Hz, 1H), 5.75 (d, *J* = 4.0 Hz, 1H), 5.42 (t, *J* = 8.6 Hz, 1H), 3.76 (d, *J* = 7.1 Hz, 1H), 1.19 (s, 9H); ^13^C NMR (101 MHz, CDCl_3_) *δ* 193.9, 159.1, 134.2, 134.1, 130.6, 129.4, 128.8, 126.5, 125.6, 122.1, 110.5, 89.3, 61.7, 56.3, 22.7; HRMS (ESI) calcd for C_19_H_22_NO_3_S [M + H]^+^: 344.1315, found 344.1312.

#### *trans*-(*S*)-*N*-(2*R*,3*R*)-5-Chloro-2-benzoyl-2,3-dihydrobenzofuran-3-yl-2-methylpropane-2-sulfinamide (3b)

White solid. Mp: 56–58 °C; [α]^20^_D_ = −52.7 [*c* = 0.74, CHCl_3_]; ^1^H NMR (400 MHz, CDCl_3_) *δ* 8.07–7.95 (m, 2H), 7.58 (t, *J* = 7.4 Hz, 1H), 7.49–7.40 (m, 3H), 7.14 (dd, *J* = 8.6, 2.2 Hz, 1H), 6.78 (d, *J* = 8.6 Hz, 1H), 5.81 (d, *J* = 5.0 Hz, 1H), 5.41 (dd, *J* = 8.0, 5.0 Hz, 1H), 3.84 (d, *J* = 8.0 Hz, 1H), 1.19 (s, 9H); ^13^C NMR (101 MHz, CDCl_3_) *δ* 192.4, 156.6, 133.2, 133.0, 129.7, 128.3, 127.9, 126.3, 125.9, 125.4, 110.6, 88.6, 60.2, 55.4, 21.6; HRMS (ESI) calcd for C_19_H_21_ClNO_3_S [M + H]^+^: 378.0925, found 378.0923.

#### *trans*-(*S*)-*N*-((2*R*,3*R*)-2-Benzoyl-5-bromo-2,3-dihydrobenzofuran-3-yl)-2-methylpropane-2-sulfinamide (3c)

White solid. Mp 60–62 °C; [α]^20^_D_ = −37.22 [*c* = 0.72, CHCl_3_]; ^1^H NMR (400 MHz, CDCl_3_) *δ* 8.00 (dd, *J* = 4.3, 7.5 Hz, 2H), 7.52 (d, *J* = 9.3 Hz, 2H), 7.41 (q, *J* = 7.6 Hz, 2H), 7.28–7.17 (m, 1H), 6.69 (t, *J* = 10.0 Hz, 1H), 6.02–5.72 (m, 1H), 5.41–5.26 (m, 1H), 3.76 (dd, *J* = 52.1, 9.3 Hz, 1H), 1.14 (s, 9H); ^13^C NMR (101 MHz, CDCl_3_) *δ* 192.4, 192.2, 157.2, 157.2, 133.2, 132.9, 132.5, 128.5, 128.3, 127.9, 126.9, 112.8, 112.5, 111.3, 111.11, 88.4, 87.8, 60.2, 59.8, 55.6, 55.4, 21.6, 21.6; HRMS (ESI) calcd for C_19_H_21_BrNO_3_S [M + H]^+^: 422.0420, found 422.0420.

#### *trans*-(*S*)-*N*-((2*R*,3*R*)-2-Benzoyl-5,7-dichloro-2,3-dihydrobenzofuran-3-yl)-2-methylpropane-2-sulfinamide (3d)

White solid. Mp: 140–142 °C; [α]^20^_D_ = −55.82 [*c* = 0.67, CHCl_3_]; ^1^H NMR (400 MHz, CDCl_3_) *δ* 8.02 (d, *J* = 7.7 Hz, 2H), 7.58 (d, *J* = 6.7 Hz, 1H), 7.47 (t, *J* = 7.2 Hz, 2H), 7.36 (s, 1H), 7.20 (s, 1H), 5.84 (d, *J* = 4.3 Hz, 1H), 5.70–5.37 (m, 1H), 3.83 (d, *J* = 8.6 Hz, 1H), 1.19 (s, 9H); ^13^C NMR (101 MHz, CDCl_3_) *δ* 191.6, 152.8, 133.4, 132.8, 129.4, 128.4, 127.9, 127.6, 126.2, 124.0, 115.4, 88.7, 61.0, 55.5, 21.6; HRMS (ESI) calcd for C_19_H_20_Cl_2_NO_3_S [M + H]^+^: 412.0535, found 412.0530.

#### *cis*-(*S*)-*N*-((2*S*,3*R*)-2-Benzoyl-5,7-dichloro-2,3-dihydrobenzofuran-3-yl)-2-methylpropane-2-sulfinamide (3d)

White solid. Mp: 133–134 °C; [α]^20^_D_ = +65.62 [*c* = 0.77, CHCl_3_]; ^1^H NMR (400 MHz, CDCl_3_) *δ* 8.20 (d, *J* = 8.2 Hz, 2H), 7.66 (t, *J* = 6.9 Hz, 1H), 7.56 (t, *J* = 7.7 Hz, 2H), 7.29 (d, *J* = 4.5 Hz, 1H), 7.19 (s, 1H), 6.19 (d, *J* = 3.3 Hz, 1H), 5.46 (dd, *J* = 11.0, 2.8 Hz, 1H), 3.93 (d, *J* = 11.0 Hz, 1H), 1.27 (s, 9H); ^13^C NMR (101 MHz, CDCl_3_) *δ* 191.5, 153.1, 133.3, 132.8, 129.5, 128.5, 127.7, 127.5, 125.8, 123.2, 115.7, 88.1, 60.5, 55.6, 21.5; HRMS (ESI) calcd for C_19_H_20_Cl_2_NO_3_S [M + H]^+^: 412.0535, found 412.0530.

#### *trans*-(*S*)-*N*-(1*R*,2*R*)-2-Benzoyl-1,2-dihydronaphtho[2,1-*b*]furan-1-yl-2-methylpropane-2-sulfinamide (3e)

White solid. Mp: 132–134 °C; [α]^20^_D_ = −173.33 [*c* = 0.09, CHCl_3_]; ^1^H NMR (400 MHz, CDCl_3_) *δ* 8.11 (d, *J* = 7.9 Hz, 2H), 7.99 (d, *J* = 8.4 Hz, 1H), 7.83 (d, *J* = 8.6 Hz, 2H), 7.65 (t, *J* = 7.1 Hz, 1H), 7.54 (t, *J* = 7.5 Hz, 3H), 7.36 (d, *J* = 8.6 Hz, 1H), 7.22 (d, *J* = 8.9 Hz, 1H), 6.18 (d, *J* = 2.9 Hz, 1H), 5.93 (s, 1H), 3.91 (d, *J* = 3.4 Hz, 1H), 1.23 (d, *J* = 20.1 Hz, 9H); ^13^C NMR (101 MHz, CDCl_3_) *δ* 193.2, 157.1, 133.0, 131.4, 129.0, 128.4, 127.8, 126.6, 122.8, 121.6, 114.7, 111.3, 89.5, 58.9, 55.2, 21.6; HRMS (ESI) calcd for C_23_H_24_NO_3_S [M + H]^+^: 394.1471, found 394.1472.

#### *trans*-(*S*)-*N*-(2*R*,3*R*)-5-Chloro-2-(4-chlorobenzoyl)-2,3-dihydrobenzofuran-3-yl-2-methylpropane-2-sulfinamide (3f)

White solid. Mp: 62–64 °C; [α]^20^_D_ = −61.72 [*c* = 0.58, CHCl_3_]; ^1^H NMR (400 MHz, CDCl_3_) *δ* 7.95 (d, *J* = 8.5 Hz, 2H), 7.42 (d, *J* = 8.5 Hz, 3H), 7.13 (dd, *J* = 8.6, 1.8 Hz, 1H), 6.75 (d, *J* = 8.6 Hz, 1H), 5.74 (d, *J* = 5.2 Hz, 1H), 5.44 (dd, *J* = 7.8, 5.2 Hz, 1H), 3.87 (d, *J* = 7.8 Hz, 1H), 1.18 (s, 9H); ^13^C NMR (101 MHz, CDCl_3_) *δ* 191.4, 156.4, 139.8, 131.4, 129.8, 129.7, 128.2, 126.2, 126.0, 125.4, 110.6, 88.5, 59.9, 55.5, 21.6; HRMS (ESI) calcd for C_19_H_20_Cl_2_NO_3_S [M + H]^+^: 412.0535, found 412.0530.

#### *trans*-(*S*)-*N*-((2*R*,3*R*)-5-Bromo-2-(4-chlorobenzoyl)-2,3-dihydrobenzofuran-3-yl)-2-methylpropane-2-sulfinamide (3g)

White solid. Mp: 63–65 °C; [α]^20^_D_ = −77.19 [*c* = 0.57, CHCl_3_]; ^1^H NMR (400 MHz, CDCl_3_) *δ* 8.02 (d, *J* = 8.5 Hz, 2H), 7.62 (s, 1H), 7.49 (d, *J* = 8.5 Hz, 2H), 7.35 (dd, *J* = 8.6, 1.9 Hz, 1H), 6.78 (d, *J* = 8.6 Hz, 1H), 5.80 (d, *J* = 5.2 Hz, 1H), 5.52 (dd, *J* = 7.8, 5.2 Hz, 1H), 3.88 (d, *J* = 7.8 Hz, 1H), 1.25 (s, 9H); ^13^C NMR (101 MHz, CDCl_3_) *δ* 191.3, 157.0, 139.8, 132.6, 131.4, 129.8, 128.3, 128.2, 126.8, 113.1, 111.2, 88.5, 59.8, 55.5, 21.6; HRMS (ESI) calcd for C_19_H_20_BrClNO_3_S [M + H]^+^: 456.0030, found 456.0026.

#### *trans*-(*S*)-*N*-((2*R*,3*R*)-2-Benzoyl-5,7-dichloro-2,3-dihydrobenzofuran-3-yl)-2-methylpropane-2-sulfinamide (3h)

Light yellow solid. Mp: 74–76 °C; [α]^20^_D_ = −137.93 [*c* = 0.29, CHCl_3_]; ^1^H NMR (400 MHz, CDCl_3_) *δ* 8.12–7.95 (m, 2H), 7.45 (dd, *J* = 8.5, 6.2 Hz, 2H), 7.35 (d, *J* = 1.2 Hz, 0.5H), 7.22 (dd, *J* = 10.1, 1.8 Hz, 1H), 7.11 (d, *J* = 1.4 Hz, 0.5H), 5.91 (dd, *J* = 11.3, 4.4 Hz, 2H), 5.46 (ddd, *J* = 14.8, 9.8, 4.4 Hz, 1H), 3.78 (d, *J* = 12.0 Hz, 1H), 1.19 (s, 9H); ^13^C NMR (101 MHz, CDCl_3_) *δ* 190.7, 190.5, 152.9, 152.6, 140.0, 139.9, 131.2, 123.0, 129.8, 129.6, 129.5, 128.3, 128.2, 127.5, 127.4, 126.4, 126.0, 123.9, 123.2, 115.7, 115.5, 88.7, 88.0, 60.6, 60.5, 55.7, 55.6, 21.6, 21.5; HRMS (ESI) calcd for C_19_H_20_Cl_2_NO_3_S [M + H]^+^: 412.0535, found 412.0536.

#### *trans*-(*S*)-*N*-((1*R*,2*R*)-2-(4-Chlorobenzoyl)-1,2-dihydronaphtho[2,1-*b*]furan-1-yl)-2-methylpropane-2-sulfinamide (3i)

Light yellow solid. Mp: 75–77 °C; [α]^20^_D_ = −47.58 [*c* = 1.91, CHCl_3_]; ^1^H NMR (400 MHz, CDCl_3_) *δ* 8.20 (d, *J* = 8.5 Hz, 2H), 7.89–7.79 (m, 2H), 7.73 (d, *J* = 8.3 Hz, 1H), 7.54 (d, *J* = 8.5 Hz, 2H), 7.48 (t, *J* = 7.5 Hz, 1H), 7.35 (t, *J* = 7.5 Hz, 1H), 7.23 (d, *J* = 8.9 Hz, 1H), 6.31 (d, *J* = 2.0 Hz, 1H), 5.77 (dd, *J* = 11.3, 2.0 Hz, 1H), 3.79 (d, *J* = 11.5 Hz, 1H), 1.26 (s, 9H); ^13^C NMR (101 MHz, CDCl_3_) *δ* 192.3, 156.8, 139.5, 131.6, 131.4, 129.9, 129.0, 128.9, 128.1, 127.9, 126.7, 122.9, 121.6, 114.6, 111.2, 89.5, 58.6, 55.2, 21.5; HRMS (ESI) calcd for C_23_H_23_ClNO_3_S [M + H]^+^: 428.1082, found 428.1079.

#### *trans*-(*S*)-2-Methyl-*N*-((1*R*,2*R*)-2-(4-methylbenzoyl)-1,2-dihydronaphtho[2,1-*b*]furan-1-yl)propane-2-sulfinamide (3j)

Light yellow solid. Mp: 133–135 °C; [α]^20^_D_ = −75.16 [*c* = 0.91, CHCl_3_]; ^1^H NMR (400 MHz, CDCl_3_) *δ* 8.18 (d, *J* = 8.2 Hz, 2H), 7.88–7.81 (m, 2H), 7.73 (d, *J* = 8.4 Hz, 1H), 7.54–7.45 (m, 1H), 7.43–7.33 (m, 3H), 7.29 (s, 1H), 6.38 (d, *J* = 2.1 Hz, 1H), 5.77 (dd, *J* = 11.4, 2.1 Hz, 1H), 3.81 (d, *J* = 11.4 Hz, 1H), 2.48 (s, 3H), 1.27 (s, 9H); ^13^C NMR (101 MHz, CDCl_3_) *δ* 192.2, 156.9, 144.0, 131.2, 130.5, 129.3, 129.0, 128.6, 128.6, 127.9, 126.3, 122.6, 121.4, 115.5, 111.4, 88.0, 60.6, 55.7, 21.6, 20.8; HRMS (ESI) calcd for C_24_H_26_NO_3_S [M + H]^+^: 408.1628, found 408.1628.

#### *trans*-(*S*)-*N*-((2*R*,3*R*)-5-Bromo-2-(4-methylbenzoyl)-2,3-dihydrobenzofuran-3-yl)-2-methylpropane-2-sulfinamide (3k)

Light yellow solid. Mp: 147–149 °C; [α]^20^_D_ = −40.96 [*c* = 0.83, CHCl_3_]; ^1^H NMR of *trans* (400 MHz, CDCl_3_) *δ* 7.95 (d, *J* = 8.2 Hz, 2H), 7.61 (d, *J* = 1.5 Hz, 1H), 7.37–7.28 (m, 3H), 6.79 (d, *J* = 8.6 Hz, 1H), 5.84 (d, *J* = 5.0 Hz, 1H), 5.44 (dd, *J* = 8.0, 5.0 Hz, 1H), 3.93 (d, *J* = 8.0 Hz, 1H), 2.44 (s, 3H), 1.25 (s, 9H); ^13^C NMR (101 MHz, CDCl_3_) *δ* 191.9, 157.2, 144.3, 132.4, 130.4, 128.6, 128.4, 128.3, 126.9, 112.8, 111.1, 88.4, 60.3, 55.4, 21.6, 20.8; HRMS (ESI) calcd for C_20_H_23_BrNO_3_S [M + H]^+^: 436.0577, found 436.0571.

#### *trans*-(*S*)-*N*-((2*R*,3*R*)-5-Chloro-2-(4-methylbenzoyl)-2,3-dihydrobenzofuran-3-yl)-2-methylpropane-2-sulfinamide (3l)

Light yellow solid. Mp: 146–148 °C; [α]^20^_D_ = −51.45 [*c* = 1.47, CHCl_3_]; ^1^H NMR (400 MHz, CDCl_3_) *δ* 7.95 (d, *J* = 8.1 Hz, 2H), 7.48 (d, *J* = 1.5 Hz, 1H), 7.30 (d, *J* = 8.0 Hz, 2H), 7.18 (dd, *J* = 8.6, 2.0 Hz, 1H), 6.82 (d, *J* = 8.6 Hz, 1H), 5.84 (d, *J* = 5.0 Hz, 1H), 5.43 (dd, *J* = 8.0, 5.0 Hz, 1H), 3.95 (d, *J* = 8.0 Hz, 1H), 2.44 (s, 3H), 1.25 (s, 9H); ^13^C NMR (101 MHz, CDCl_3_) *δ* 192.0, 156.7, 144.3, 130.4, 129.5, 128.5, 128.4, 126.4, 125.7, 125.4, 110.5, 88.4, 60.4, 55.4, 21.6, 20.8; HRMS (ESI) calcd for C_20_H_23_ClNO_3_S [M + H]^+^: 392.1082, found 392.1077.

#### *trans*-(*R*)-*N*-((2*S*,3*S*)-2-Benzoyl-2,3-dihydrobenzofuran-3-yl)-2-methylpropane-2-sulfinamide (3a)

White solid. Mp: 55–57 °C. [α]^20^_D_ = +68.7 [*c* = 0.88, CHCl_3_]; ^1^H NMR (400 MHz, CDCl_3_) *δ* 8.21 (d, *J* = 8.0 Hz, 1H), 8.10 (d, *J* = 8.0 Hz, 1H), 7.66–7.53 (m, 4H), 7.29–7.25 (m, 1H), 7.07–6.90 (m, 2H), 6.17–5.80 (m, 1H), 5.48 (ddd, *J* = 14.2, 9.5, 4.1 Hz, 1H), 3.87 (dd, *J* = 8.0, 8.0 Hz, 1H), 1.27 (d, *J* = 3.7 Hz, 9H); ^13^C NMR (101 MHz, CDCl_3_) *δ* 192.9, 158.2, 133.2, 133.0, 129.7, 128.5, 127.8, 125.7, 124.8, 121.1, 109.7, 88.3, 60.6, 55.5, 21.7; HRMS (ESI) calcd for C_19_H_22_NO_3_S [M + H]^+^: 344.1315, found 344.1311.

#### *trans*-(*R*)-*N*-((2*S*,3*S*)-2-Benzoyl-5-chloro-2,3-dihydrobenzofuran-3-yl)-2-methylpropane-2-sulfinamide (3b)

White solid. Mp: 59–61 °C; [α]^20^_D_ = +55.17 [*c* = 0.95, CHCl_3_]; ^1^H NMR (400 MHz, CDCl_3_) *δ* 8.20 (d, *J* = 7.7 Hz, 2H), 7.67 (t, *J* = 7.2 Hz, 1H), 7.57 (t, *J* = 7.5 Hz, 2H), 7.30–7.23 (m, 2H), 6.91 (d, *J* = 8.5 Hz, 1H), 6.14 (d, *J* = 3.0 Hz, 1H), 5.44 (dd, *J* = 11.0, 3.0 Hz, 1H), 3.79 (d, *J* = 11.0 Hz, 1H), 1.28 (s, 9H); ^13^C NMR (101 MHz, CDCl_3_) *δ* 192.2, 156.8, 133.2, 133.0, 129.7, 128.5, 127.9, 126.4, 125.5, 124.8, 110.8, 87.8, 59.9, 55.6, 21.5; HRMS (ESI) calcd for C_19_H_21_ClNO_3_S [M + H]^+^: 378.0925, found 378.0928.

#### *trans*-(*R*)-*N*-((2*S*,3*S*)-2-Benzoyl-5-bromo-2,3-dihydrobenzofuran-3-yl)-2-methylpropane-2-sulfinamide (3c)

White solid. Mp: 61–63 °C; [α]^20^_D_ = +42.78 [*c* = 0.72, CHCl_3_]; ^1^H NMR (400 MHz, CDCl_3_) *δ* 8.02–7.97 (m, 2H), 7.66–7.49 (m, 2H), 7.44 (t, *J* = 7.7 Hz, 2H), 7.27 (dd, *J* = 8.6, 1.9 Hz, 1H), 6.72 (d, *J* = 8.6 Hz, 1H), 5.79 (d, *J* = 5.1 Hz, 1H), 5.39 (dd, *J* = 8.2, 5.1 Hz, 1H), 3.89 (d, *J* = 8.2 Hz, 1H), 1.18 (s, 9H); ^13^C NMR (101 MHz, CDCl_3_) *δ* 193.4, 158.2, 134.3, 134.0, 133.5, 129.4, 129.4, 128.9, 127.9, 113.9, 112.2, 89.5, 61.2, 56.5, 22.7; HRMS (ESI) calcd for C_19_H_21_BrNO_3_S [M + H]^+^: 422.0420, found 422.0412.

#### *trans*-(*R*)-*N*-((2*S*,3*S*)-2-Benzoyl-5,7-dichloro-2,3-dihydrobenzofuran-3-yl)-2-methylpropane-2-sulfinamide (3d)

White solid. Mp: 142–144 °C; [α]^20^_D_ = +51.02 [*c* = 0.66, CHCl_3_]; ^1^H NMR (400 MHz, CDCl_3_) *δ* 8.01 (d, *J* = 7.7 Hz, 2H), 7.58 (t, *J* = 7.4 Hz, 1H), 7.45 (t, *J* = 7.7 Hz, 2H), 7.35 (s, 1H), 7.20–7.17 (m, 1H), 5.82 (d, *J* = 5.2 Hz, 1H), 5.46 (dd, *J* = 8.4, 5.2 Hz, 1H), 3.88 (d, *J* = 8.4 Hz, 1H), 1.18 (s, 9H); ^13^C NMR (101 MHz, CDCl_3_) *δ* 153.8, 134.4, 133.8, 130.5, 129.4, 129.0, 128.6, 127.3, 125.0, 116.5, 89.8, 62.0, 56.6, 22.7; HRMS (ESI) calcd for C_19_H_20_Cl_2_NO_3_S [M + H]^+^: 412.0535, found 412.0539.

#### *trans*-(*R*)-*N*-((1*S*,2*S*)-2-Benzoyl-1,2-dihydronaphtho[2,1-*b*]furan-1-yl)-2-methylpropane-2-sulfinamide (3e)

White solid. Mp: 133–135 °C; [α]^20^_D_ = +122.2 [*c* = 0.36, CHCl_3_]; ^1^H NMR (400 MHz, CDCl_3_) *δ* 8.25 (d, *J* = 7.4 Hz, 2H), 7.86–7.80 (m, 2H), 7.73 (d, *J* = 8.3 Hz, 1H), 7.65 (t, *J* = 7.3 Hz, 1H), 7.57 (t, *J* = 7.6 Hz, 2H), 7.47 (t, *J* = 7.3 Hz, 1H), 7.35 (t, *J* = 7.3 Hz, 1H), 7.24 (d, *J* = 9.0 Hz, 1H), 6.38 (d, *J* = 2.1 Hz, 1H), 5.82–5.74 (m, 1H), 3.79 (dd, *J* = 16.4, 8.4 Hz, 1H), 1.25 (s, 9H); ^13^C NMR (101 MHz, CDCl_3_) *δ* 192.7, 156.8, 133.1, 133.0, 131.2, 129.3, 129.0, 128.6, 128.0, 126.3, 122.7, 121.4, 115.5, 111.4, 88.1, 60.5, 55.7, 21.6; HRMS (ESI) calcd for C_23_H_24_NO_3_S [M + H]^+^: 394.1471, found 394.1465.

#### *trans*-(*R*)-*N*-((2*S*,3*S*)-5-Chloro-2-(4-chlorobenzoyl)-2,3-dihydrobenzofuran-3-yl)-2-methylpropane-2-sulfinamide (3f)

White solid. Mp: 67–69 °C; [α]^20^_D_ = +95.63 [*c* = 0.87, CHCl_3_]; ^1^H NMR (400 MHz, CDCl_3_) *δ* 7.96 (d, *J* = 8.5 Hz, 2H), 7.43 (d, *J* = 8.5 Hz, 3H), 7.14 (dd, *J* = 8.6, 1.9 Hz, 1H), 6.76 (d, *J* = 8.6 Hz, 1H), 5.74 (d, *J* = 5.1 Hz, 1H), 5.50–5.41 (m, 1H), 3.79 (d, *J* = 7.9 Hz, 1H), 1.19 (s, 9H); ^13^C NMR (101 MHz, CDCl_3_) *δ* 192.4, 157.5, 140.8, 132.5, 130.8, 130.7, 129.2, 127.3, 127.1, 126.4, 111.6, 89.6, 60.9, 56.5, 22.7; HRMS (ESI) calcd for C_19_H_20_Cl_2_NO_3_S [M + H]^+^: 412.0535, found 412.0534.

#### *trans*-(*R*)-*N*-((2*S*,3*S*)-5-Bromo-2-(4-chlorobenzoyl)-2,3-dihydrobenzofuran-3-yl)-2-methylpropane-2-sulfinamide (3g)

White solid. Mp: 55–57 °C; [α]^20^_D_ = +57.78 [*c* = 1.17, CHCl_3_]; ^1^H NMR (400 MHz, CDCl_3_) *δ* 7.94 (d, *J* = 8.5 Hz, 2H), 7.55 (s, 1H), 7.41 (d, *J* = 8.5 Hz, 2H), 7.27 (dd, *J* = 8.6, 1.7 Hz, 1H), 6.70 (d, *J* = 8.6 Hz, 1H), 5.72 (d, *J* = 5.2 Hz, 1H), 5.42 (dd, *J* = 8.0, 5.2 Hz, 1H), 3.91 (d, *J* = 8.0 Hz, 1H), 1.18 (s, 9H); ^13^C NMR (101 MHz, CDCl_3_) *δ* 191.3, 156.9, 139.8, 132.5, 131.4, 129.8, 128.3, 128.2, 126.8, 113.0, 111.1, 88.4, 59.8, 55.5, 21.6; HRMS (ESI) calcd for C_19_H_20_BrClNO_3_S [M + H]^+^: 456.0030, found 456.0030.

#### *trans*-(*R*)-*N*-((2*S*,3*S*)-5,7-Dichloro-2-(4-chlorobenzoyl)-2,3-dihydrobenzofuran-3-yl)-2-methylpropane-2-sulfinamide (3h)

White solid. Mp: 58–60 °C; [α]^20^_D_ = +43.98 [*c* = 0.39, CHCl_3_]; ^1^H NMR (400 MHz, CDCl_3_) *δ* 8.18–8.01 (m, 2H), 7.59–7.47 (m, 2H), 7.42–7.27 (m, 1H), 7.26–7.16 (m, 1H), 6.15–5.80 (m, 1H), 5.63–5.39 (m, 1H), 3.91 (dd, *J* = 48.6, 9.9 Hz, 1H), 1.25 (d, *J* = 1.0 Hz, 9H); ^13^C NMR (101 MHz, CDCl_3_) *δ* 190.7, 190.5, 152.9, 152.6, 140.0, 139.9, 131.2, 131.1, 123.0, 129.8, 129.6, 129.5, 128.3, 128.3, 127.5, 127.4, 126.3, 126.0, 123.9, 123.2, 115.8, 115.5, 88.8, 88.0, 60.6, 60.5, 55.7, 55.5, 21.6, 21.5; HRMS (ESI) calcd for C_19_H_19_Cl_3_NO_3_S [M + H]^+^: 446.0146, found 446.0140.

#### *trans*-(*R*)-*N*-((1*S*,2*S*)-2-(4-Chlorobenzoyl)-1,2-dihydronaphtho[2,1-*b*]furan-1-yl)-2-methylpropane-2-sulfinamide (3i)

White solid. Mp: 67–69 °C; [α]^20^_D_ = +43.98 [*c* = 0.64, CHCl_3_]; ^1^H NMR (400 MHz, CDCl_3_) *δ* 8.13 (dd, *J* = 56.2, 8.6 Hz, 2H), 7.90 (dd, *J* = 67.2, 8.6 Hz, 3H), 7.59–7.45 (m, 3H), 7.36 (t, *J* = 7.2 Hz, 1H), 7.20 (dd, *J* = 13.2, 8.9 Hz, 1H), 6.22 (dd, *J* = 75.4, 2.6 Hz, 1H), 6.01–5.71 (m, 1H), 3.92 (dd, *J* = 57.1, 7.9 Hz, 1H), 1.22 (d, *J* = 24.2 Hz, 9H); ^13^C NMR (101 MHz, CDCl_3_) *δ* 192.3, 156.8, 139.5, 131.6, 131.4, 129.9, 129.0, 128.9, 128.1, 127.9, 126.7, 122.9, 121.6, 114.6, 111.2, 89.5, 58.6, 55.2, 21.5; HRMS (ESI) calcd for C_23_H_23_ClNO_3_S [M + H]^+^: 428.1082, found 428.1080.

#### *trans*-(*R*)-2-Methyl-*N*-((1*S*,2*S*)-2-(4-methylbenzoyl)-1,2-dihydronaphtho[2,1-*b*]furan-1-yl)propane-2-sulfinamide (3j)

White solid. Mp: 142–144 °C; [α]^20^_D_ = +70.47 [*c* = 0.92, CHCl_3_]; ^1^H NMR (400 MHz, CDCl_3_) *δ* 8.18 (d, *J* = 8.2 Hz, 2H), 7.88–7.82 (m, 2H), 7.73 (d, *J* = 8.4 Hz, 1H), 7.48 (t, *J* = 7.3 Hz, 1H), 7.37 (dd, *J* = 14.5, 7.5 Hz, 3H), 7.28 (s, 1H), 6.38 (d, *J* = 2.0 Hz, 1H), 5.77 (dd, *J* = 11.3, 2.0 Hz, 1H), 3.82 (d, *J* = 11.3 Hz, 1H), 2.48 (s, 3H), 1.27 (s, 9H); ^13^C NMR (101 MHz, CDCl_3_) *δ* 192.2, 156.9, 144.0, 131.2, 130.5, 129.3, 129.0, 128.7, 128.6, 127.9, 126.3, 122.6, 121.4, 115.6, 111.4, 88.0, 60.6, 55.7, 21.6, 20.8; HRMS (ESI) calcd for C_24_H_26_NO_3_S [M + H]^+^: 408.1628, found 408.1622.

#### *trans*-(*R*)-*N*-((2*S*,3*S*)-5-Chloro-2-(4-methylbenzoyl)-2,3-dihydrobenzofuran-3-yl)-2-methylpropane-2-sulfinamide (3l)

Light yellow solid. Mp: 147–149 °C; [α]^20^_D_ = +54.67 [*c* = 1.20, CHCl_3_]; ^1^H NMR (400 MHz, CDCl_3_) *δ* 7.96 (d, *J* = 8.2 Hz, 2H), 7.48 (d, *J* = 1.7 Hz, 1H), 7.31 (d, *J* = 8.0 Hz, 2H), 7.19 (dd, *J* = 8.6, 2.1 Hz, 1H), 6.83 (d, *J* = 8.6 Hz, 1H), 5.84 (d, *J* = 5.0 Hz, 1H), 5.44 (dd, *J* = 8.1, 5.0 Hz, 1H), 3.91 (d, *J* = 8.1 Hz, 1H), 2.44 (s, 3H), 1.25 (s, 9H); ^13^C NMR (101 MHz, CDCl_3_) *δ* 191.9, 156.7, 144.3, 130.5, 129.5, 128.6, 128.4, 126.4, 125.8, 125.4, 110.5, 88.5, 60.4, 55.4, 21.6, 20.8; HRMS (ESI) calcd for C_20_H_23_ClNO_3_S [M + H]^+^: 392.1082, found 392.1076.

#### *trans*-(*R*)-*N*-((2*S*,3*S*)-5-Bromo-2-(4-methylbenzoyl)-2,3-dihydrobenzofuran-3-yl)-2-methylpropane-2-sulfinamide (3n)

White solid. Mp: 146–148 °C; [α]^20^_D_ = +37.88 [*c* = 1.16, CHCl_3_]; ^1^H NMR (400 MHz, CDCl_3_) *δ* 7.88 (d, *J* = 8.1 Hz, 2H), 7.55 (s, 1H), 7.25 (dd, *J* = 16.3, 4.8 Hz, 3H), 6.71 (d, *J* = 8.6 Hz, 1H), 5.76 (d, *J* = 5.1 Hz, 1H), 5.38 (dd, *J* = 8.0, 5.1 Hz, 1H), 3.83 (d, *J* = 8.0 Hz, 1H), 2.37 (s, 3H), 1.18 (s, 9H); ^13^C NMR (101 MHz, CDCl_3_) *δ* 192.9, 158.3, 145.4, 133.5, 131.4, 129.6, 129.5, 129.4, 128.0, 113.8, 112.1, 89.4, 61.4, 56.4, 29.7, 22.7; HRMS (ESI) calcd for C_20_H_23_BrNO_3_S [M + H]^+^: 436.0577, found 436.0573.

### General procedure for synthesis of 3-amino-2,3-dihydrobenzofuran-2-yl(aryl)methanone hydrochlorides 5

2The synthesis of 3-amino-2,3-dihydrobenzofuran-2-yl(phenyl)methanone hydrochloride 5a is representative. To a solution of *trans*-3-(2-methylpropane-2-sulfinamide)-2,3-dihydrobenzofuran 3a (68.7 mg, 0.20 mmol) in dioxane (10 mL) was added dropwise freshly prepared saturated dioxane/HCl (15 mL, ∼20 equiv. HCl). The mixture was allowed to stir for 1 h. Then the reaction mixture was concentrated *in vacuo*. Precipitation in diethyl ether afforded 42.1 mg (0.15 mmol) of pure 3-amino-2,3-dihydrobenzofuran-2-yl-(phenyl)methanone hydrochloride 5a.

#### (2*S*,3*R*)-(3-Amino-2,3-dihydro-benzofuran-2-yl)-phenyl-methanone (5a)

White solid. Mp: 161–163 °C; [α]^20^_D_ = −137.93 [*c* = 0.29 g/100 mL, CH_3_OH]; ^1^H NMR (400 MHz, CD_3_OD) *δ* 8.20–8.14 (m, 2H), 7.74 (t, *J* = 7.5 Hz, 1H), 7.61 (dd, *J* = 12.3, 4.7 Hz, 3H), 7.45–7.35 (m, 1H), 7.16–7.08 (m, 1H), 6.97 (t, *J* = 8.9 Hz, 1H), 6.13 (d, *J* = 3.0 Hz, 1H), 5.63 (d, *J* = 3.0 Hz, 1H); ^13^C NMR (101 MHz, MeOD) *δ* 193.2, 160.7, 135.6, 135.5, 133.2, 130.7, 130.0, 127.1, 123.5, 122.9, 112.0, 86.4, 54.5; HRMS (ESI) calcd for C_15_H_14_ClNO_2_ [M + H]^+^: 240.1019, found 240.1012.

## Conflicts of interest

There are no conflicts to declare.

## Supplementary Material

RA-009-C9RA00309F-s001

RA-009-C9RA00309F-s002
