# Supplementary material for: K3PO4-promoted domino reactions: diastereoselective synthesis of trans-2,3-dihydrobenzofurans from salicyl N-tert-butanesulfinyl imines and sulfur ylides
Source: RSC Adv. 2019 Apr 16;9(21):11978–85. doi: 10.1039/c9ra00309f (PMC9063524; doi:10.1039/c9ra00309f)
Supplement: RA-009-C9RA00309F-s001 [file RA-009-C9RA00309F-s001.pdf]

Electronic Supplementary Material (ESI) for RSC Advances.
This journal is © The Royal Society of Chemistry 2019

Supporting Information

K_3PO_4-Promoted Domino Reactions: Distereoselective Synthesis of *trans*-2,3-Dihydrobenzofurans from Salicyl *N*-*tert*-Butanesulfinyl imines and Sulfur Ylides

Minxuan Zhang, [a] Tianyu Lu, [a] Yun Zhao, [a] Guixian Xie, *[c] and Zhiwei Miao *[a,b]

[a] State Key Laboratory and Institute of Elemento-Organic Chemistry, College of Chemistry, Nankai University, Tianjin 300071, People's Republic of China.

[b] Collaborative Innovation Center of Chemical Science and Engineering, Nankai University, Tianjin 300071, People's Republic of China.

[c] College of Resources and Environment, Hunan Agricultural University, Changsha 410128, People's Republic of China.

Fax: +86-22-23502351; Tel: +86-22-23504783

E-mail: miaozhiwei@nankai.edu.cn

Table of Contents

Copies of ^{1}H NMR and ^{13}C NMR spectra of ***trans-(Ss,2R,3R)*-3a-3l**………………S2

Copies of ^{1}H NMR and ^{13}C NMR spectra of ***trans-(Rs,2S,3S)*-3a-3n**……..……..S14

Copies of ^{1}H NMR and ^{13}C NMR spectra of ***cis-(Ss,2R,3R)*-3a**…………….……S26

Copy of ^{1}H NMR and ^{13}C NMR spectra of **4a**…………….………..………...S27

X-ray crystallographic data of compound ***trans*-3a**……………………..………..…S28

X-ray crystallographic data of compound ***trans*-3d**……………………...…...……S30

Copies of ^{1}H NMR and ^{13}C NMR spectra of ***(Ss,2R,3R)-trans*-3a-3l**:

Trans-(S)-N-((2R,3R)-2-Benzoyl-2,3-dihydrobenzofuran-3-yl-2-methylpropane-2-sulfinamide (3a).

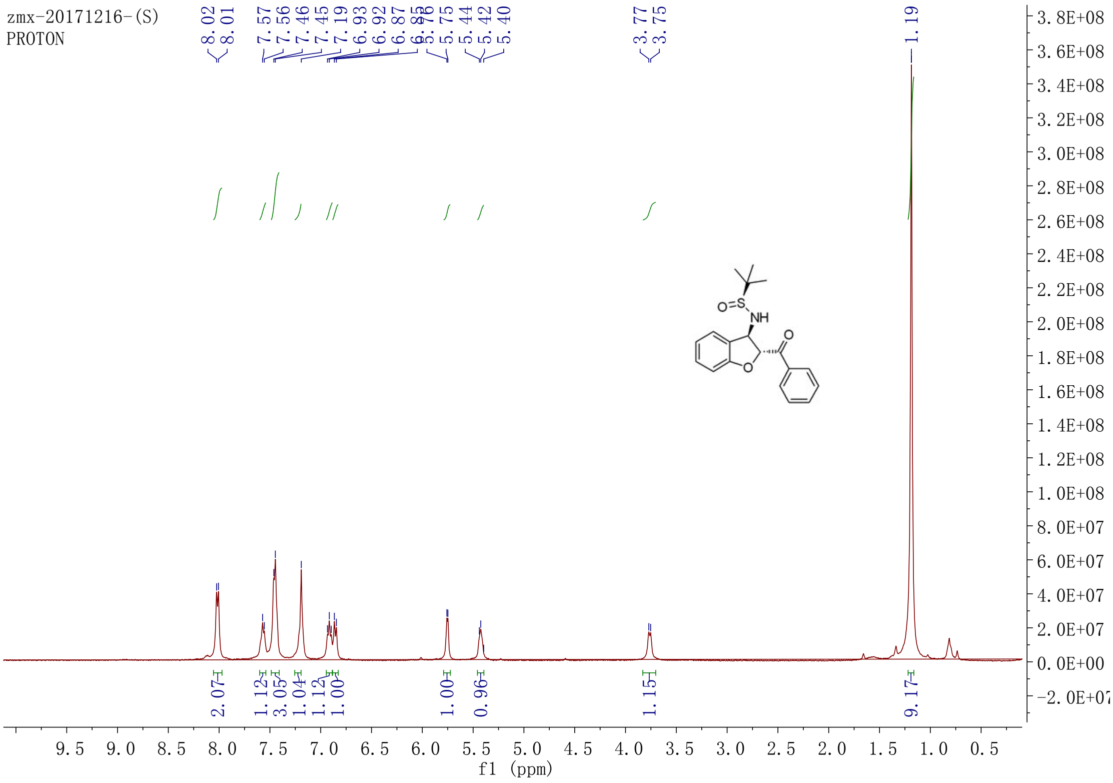

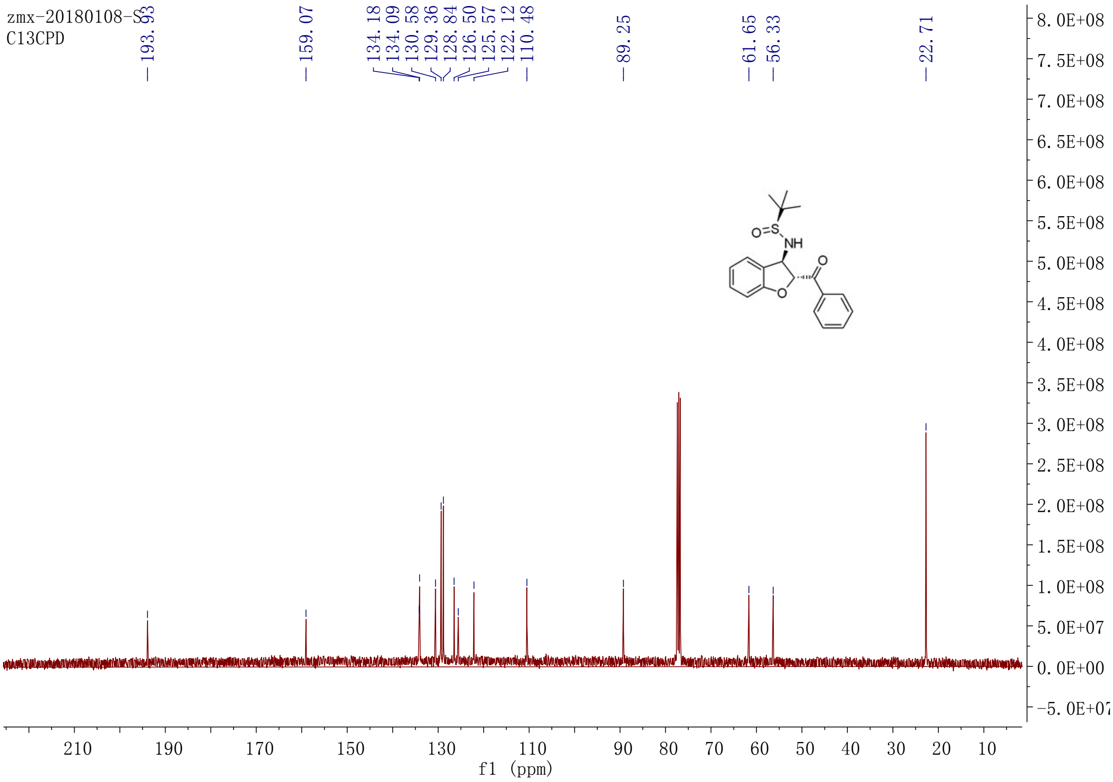

Trans-(S)-N-((2R,3R)-2-benzoyl-5-chloro-2,3-dihydrobenzofuran-3-yl)-2-methylpropane-2-sulfinamide (3b)

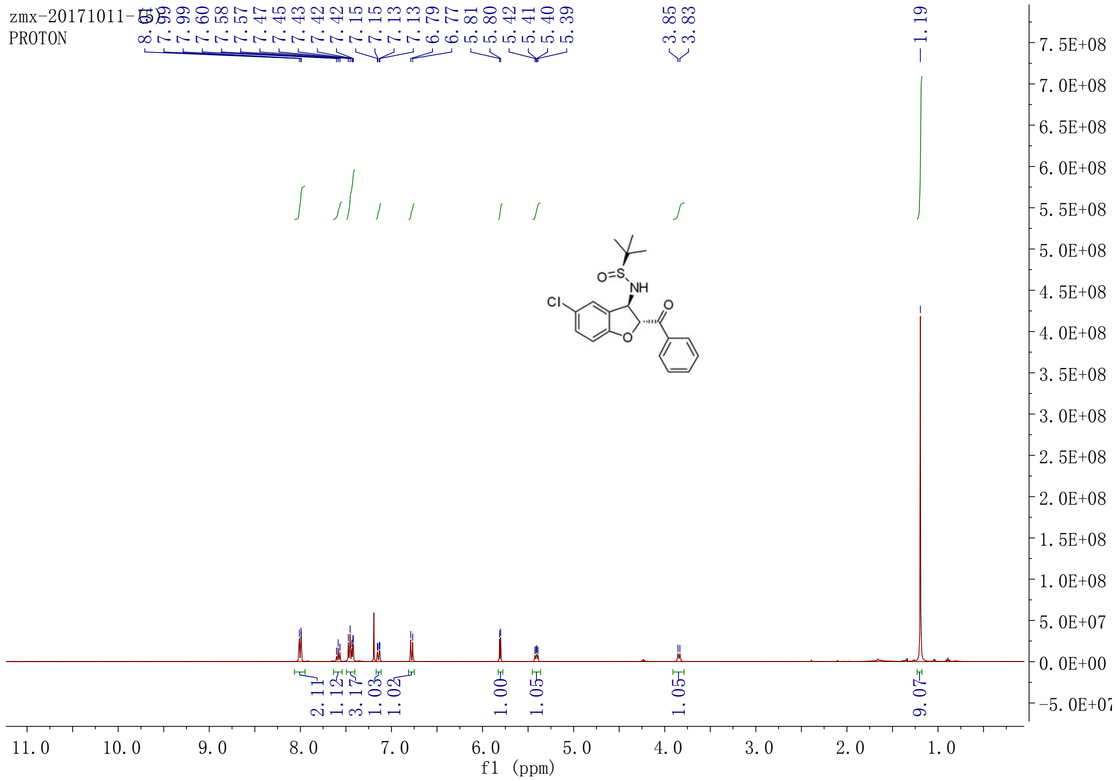

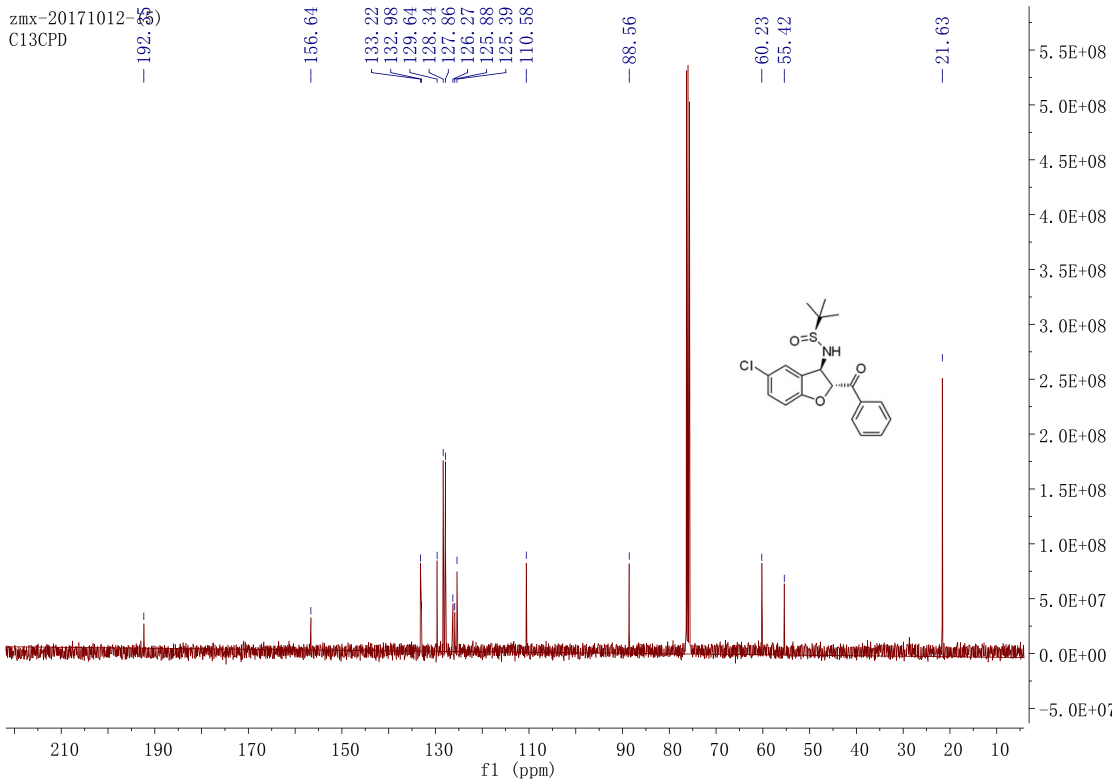

Trans-(S)-N-((2R,3R)-2-Benzoyl-5-bromo-2,3-dihydrobenzofuran-3-yl)-2-methylpropane-2-sulfinamide (3c)

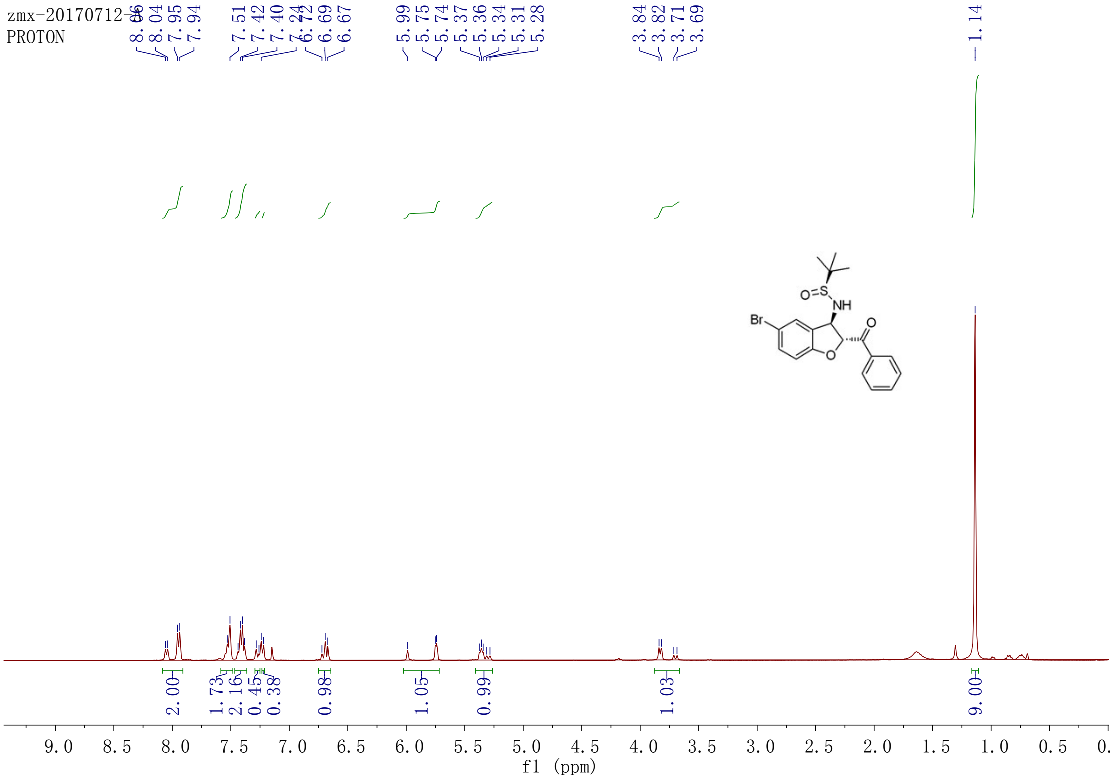

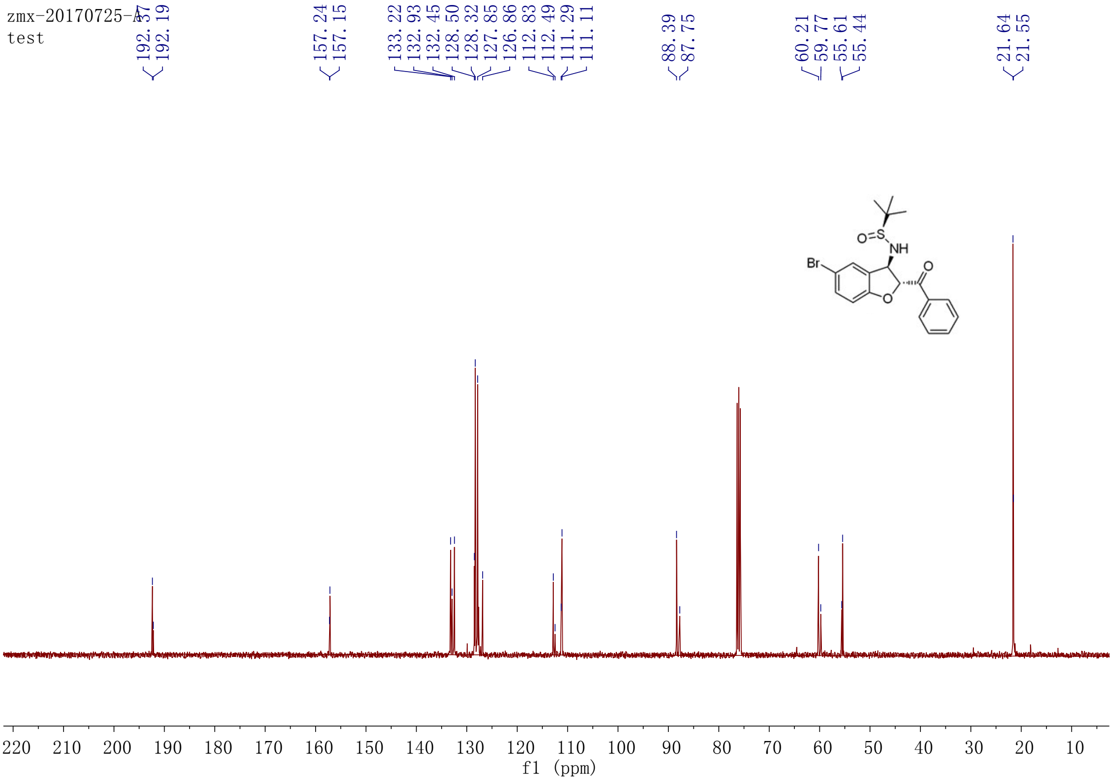

Trans-(S)-N-((2R,3R)-2-Benzoyl-5,7-dichloro-2,3-dihydrobenzofuran-3-yl)-2-methylpropane-2-sulfinamide (3d)

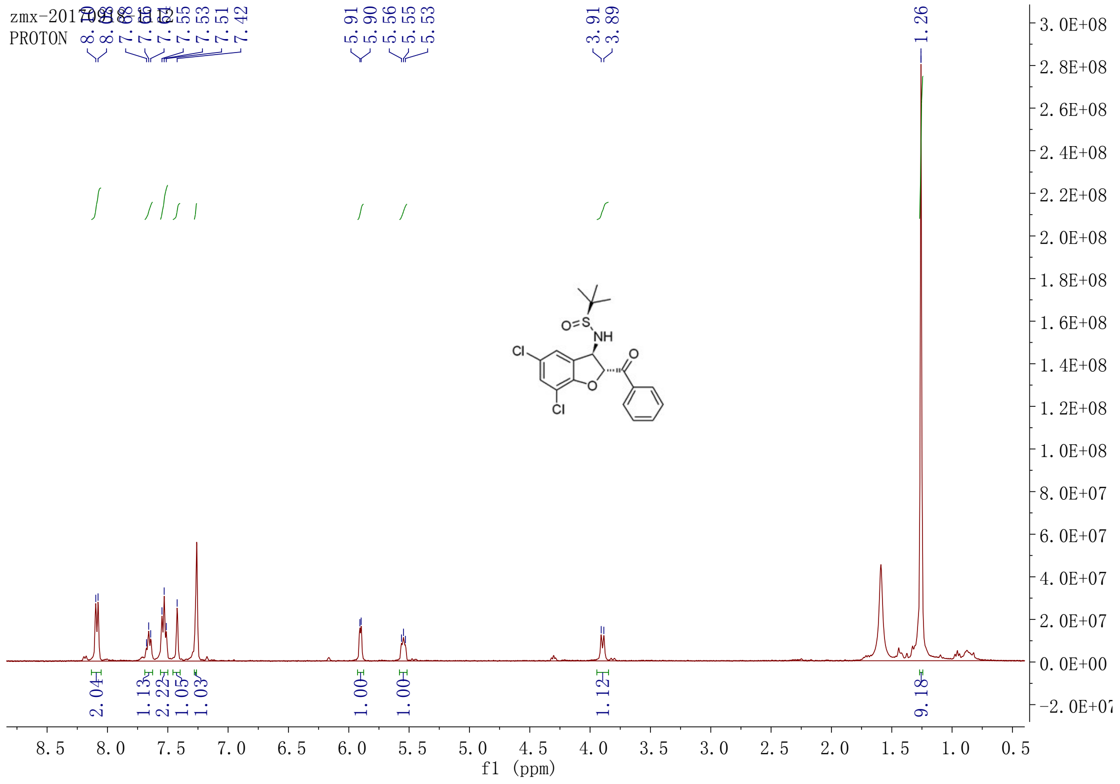

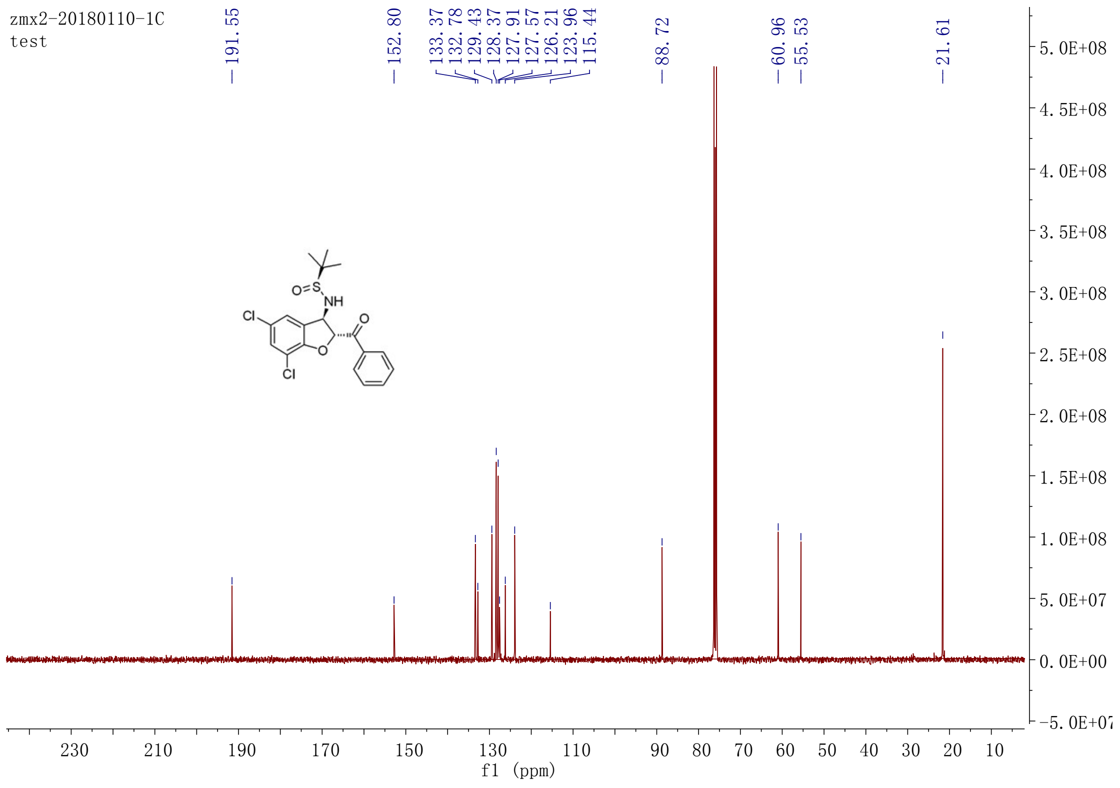

Trans-(S)-N-((1R,2R)-2-Benzoyl-1,2-dihydronaphtho[2,1-b]furan-1-yl-2-methylpropane-2-sulfinamide (3e)

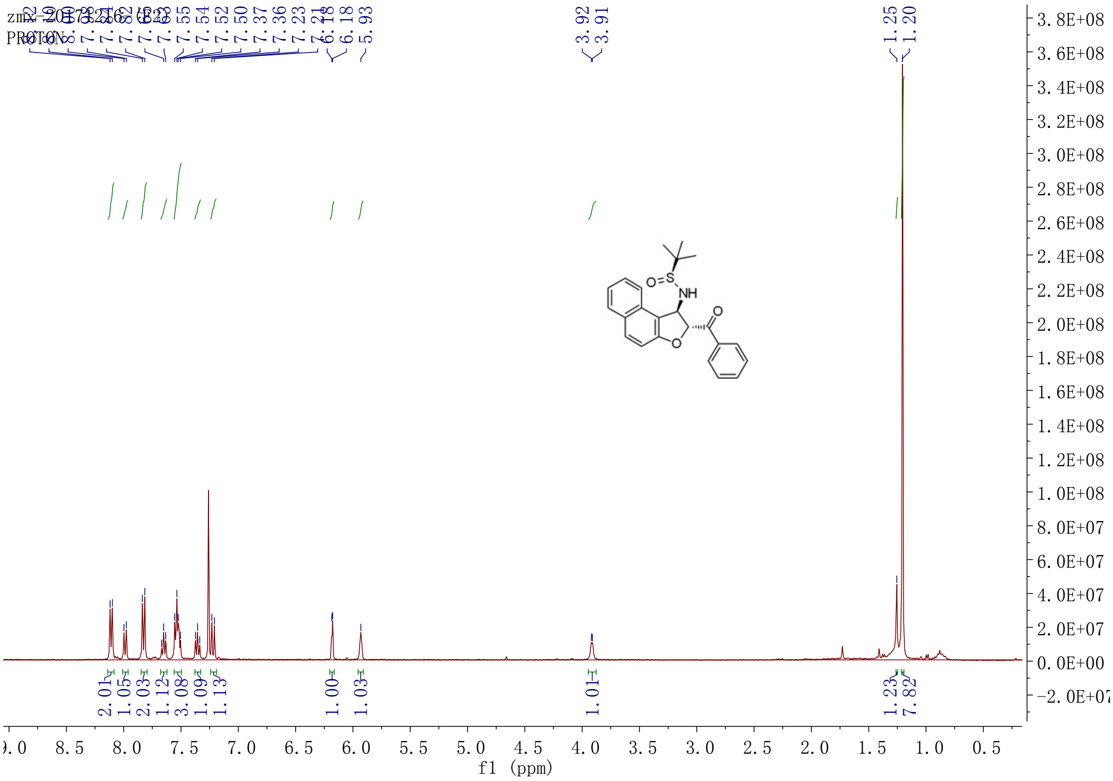

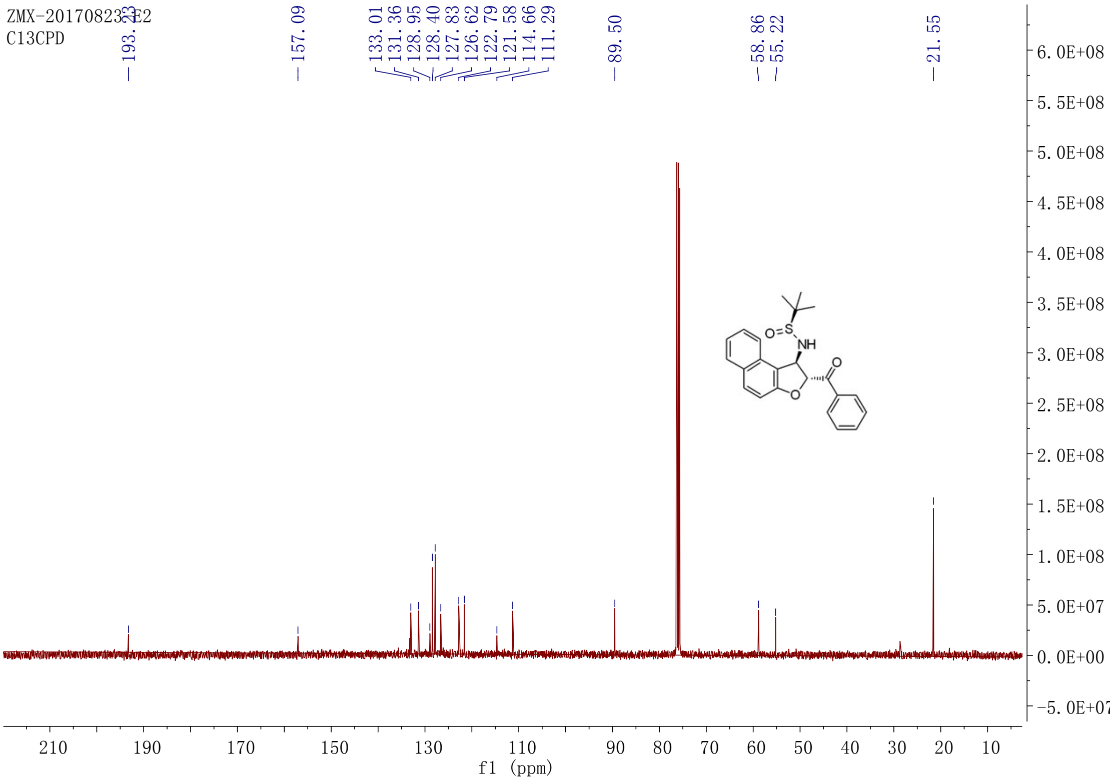

Trans-(S)-N-((2R,3R)-5-Chloro-2-(4-chlorobenzoyl)-2,3-dihydrobenzofuran-3-yl-2-methylpropane-2-sulfinamide (3f)

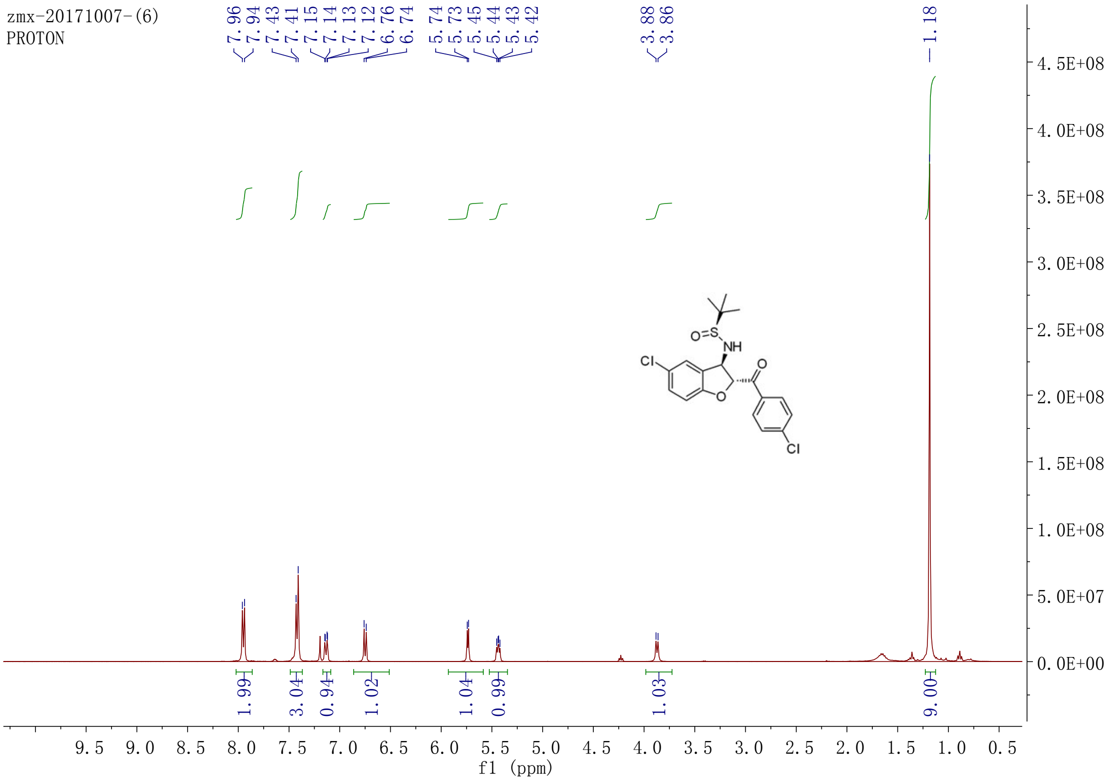

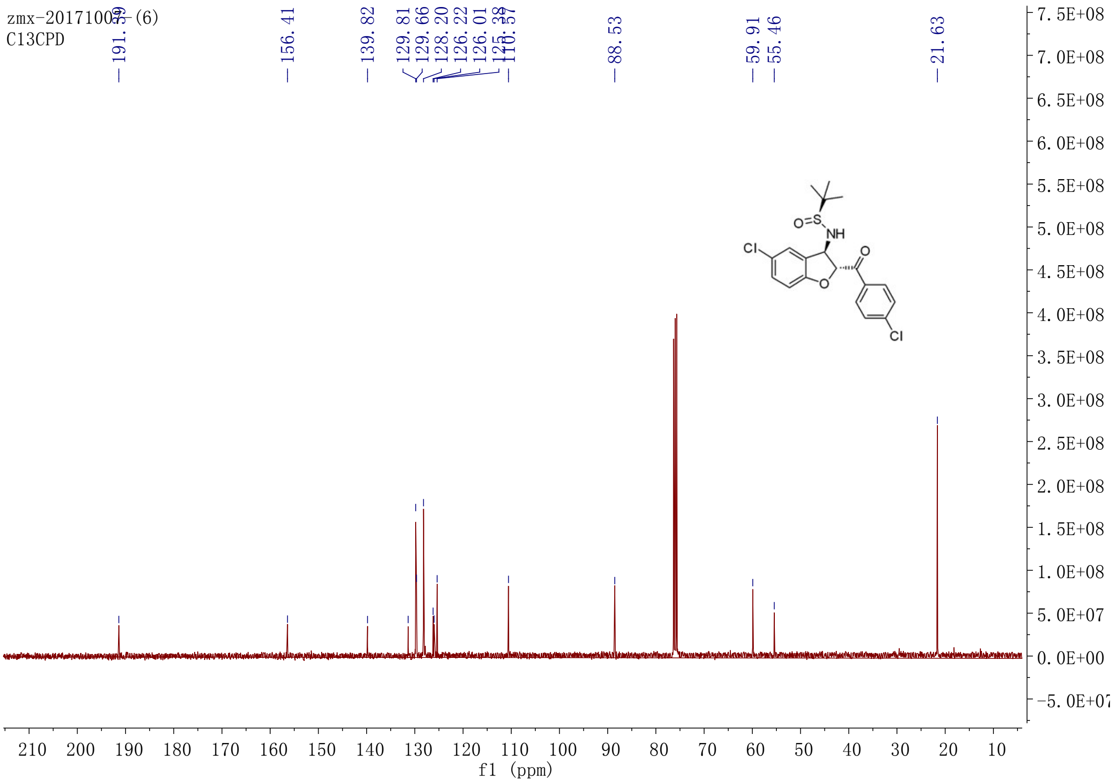

Trans-(S)-N-((2R,3R)-5-Bromo-2-(4-chlorobenzoyl)-2,3-dihydrobenzofuran-3-yl)-2-methylpropane-2-sulfinamide (3g)

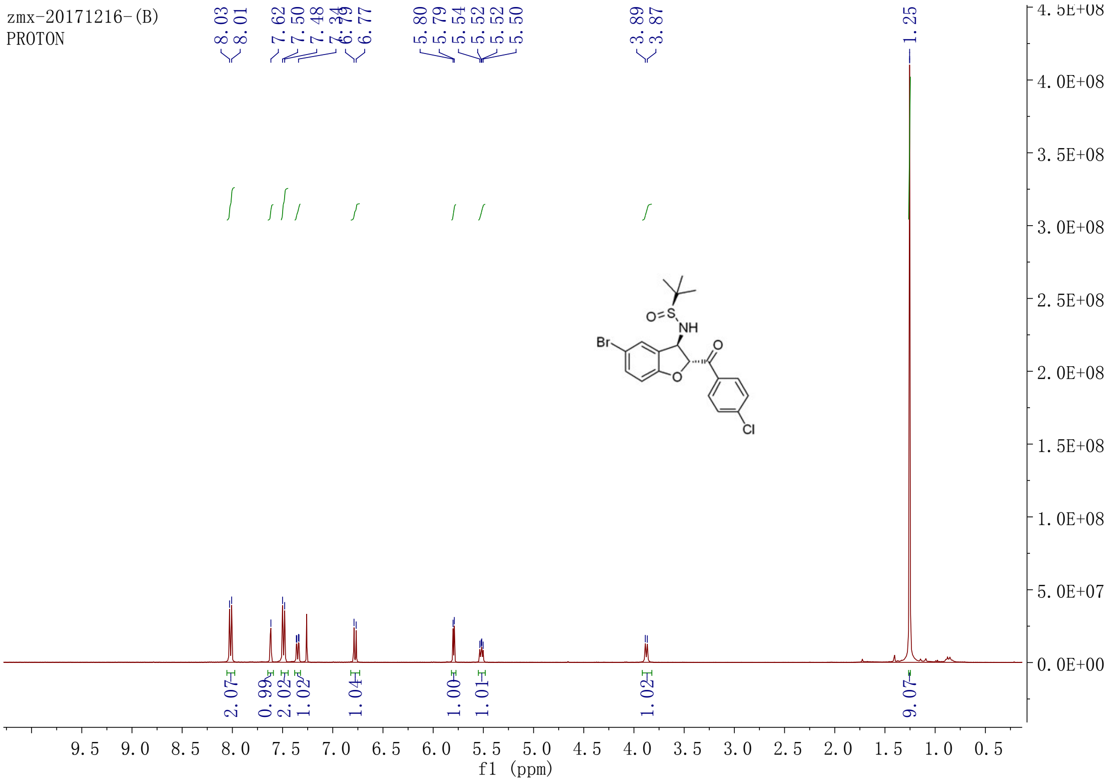

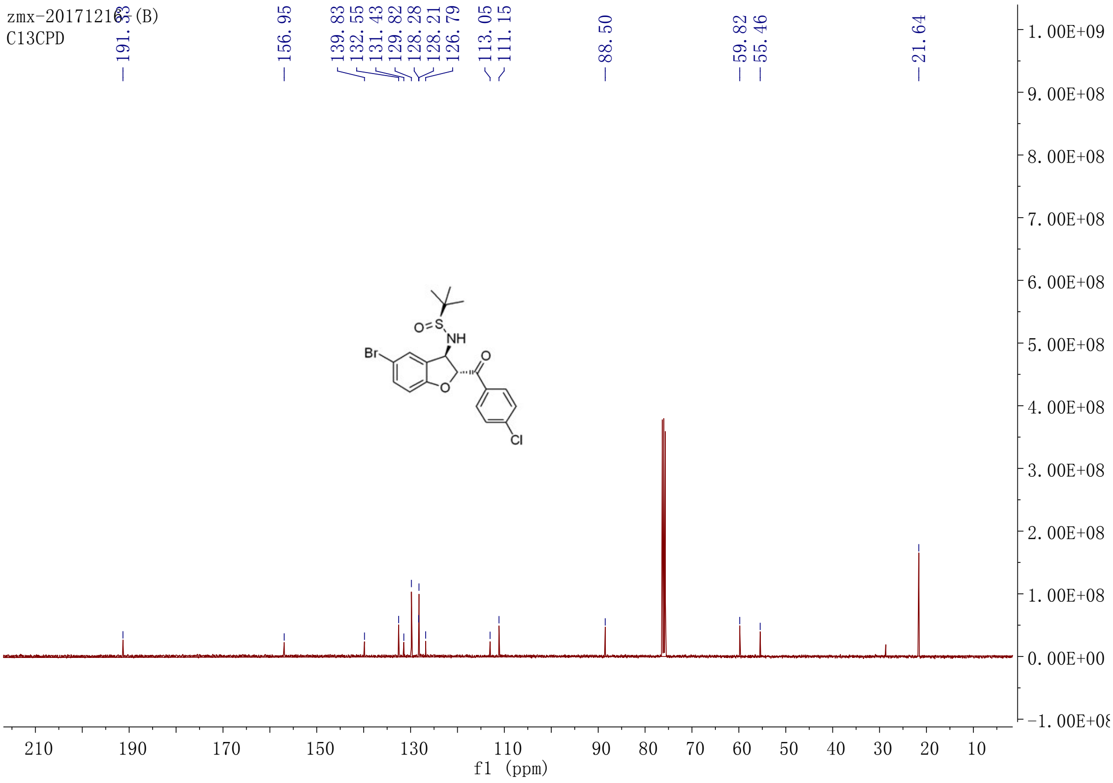

Trans-(S)-N-((2R,3R)-5,7-dichloro-2-(4-chlorobenzoyl)-2,3-dihydrobenzofuran-3-yl)-2-methylpropane-2-sulfinamide (3h)

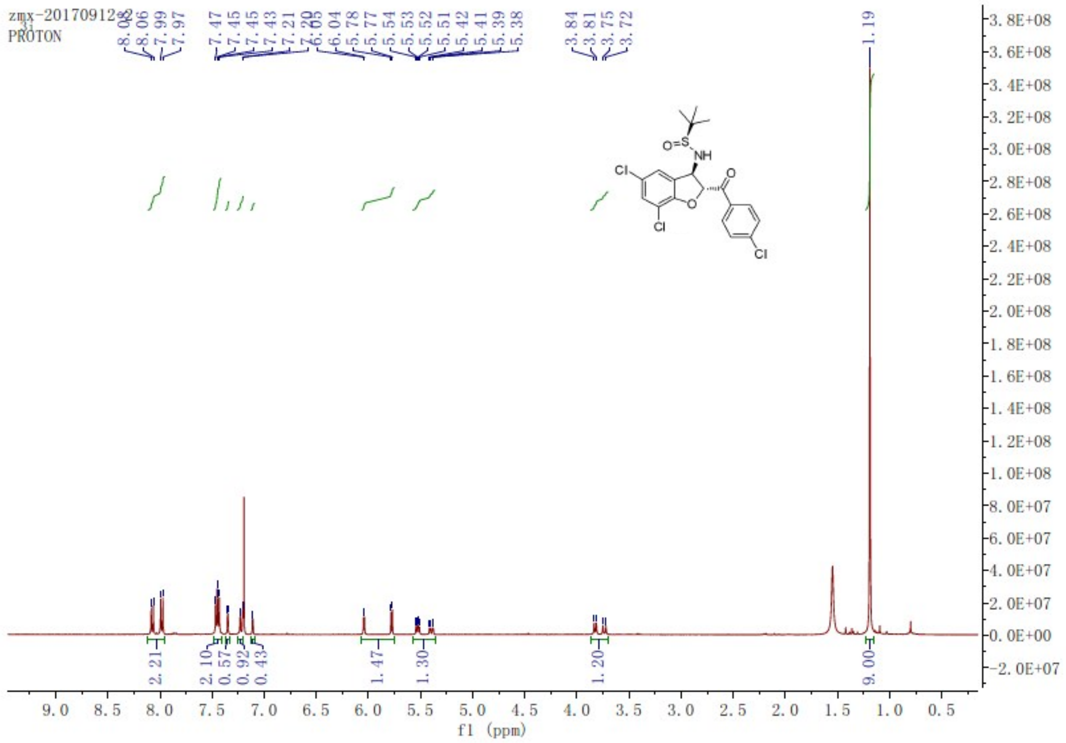

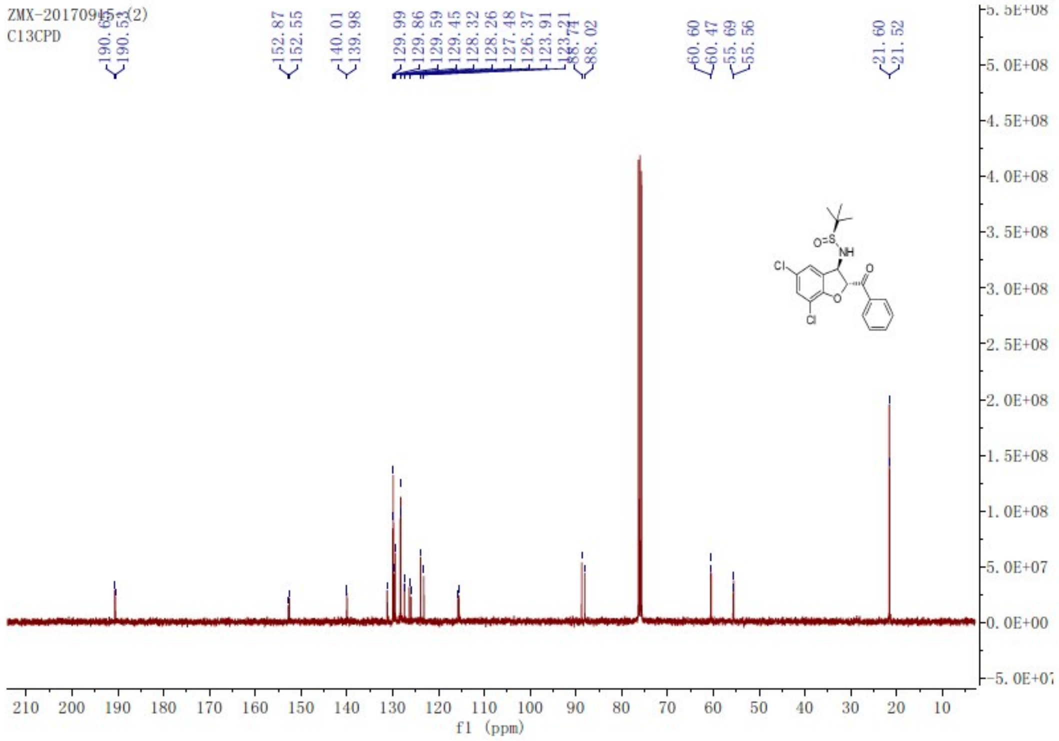

Trans-(S)-N-((1R,2R)-2-(4-Chlorobenzoyl)-1,2-dihydronaphtho[2,1-b]furan-1-yl)-2-methylpropane-2-sulfinamide (3i)

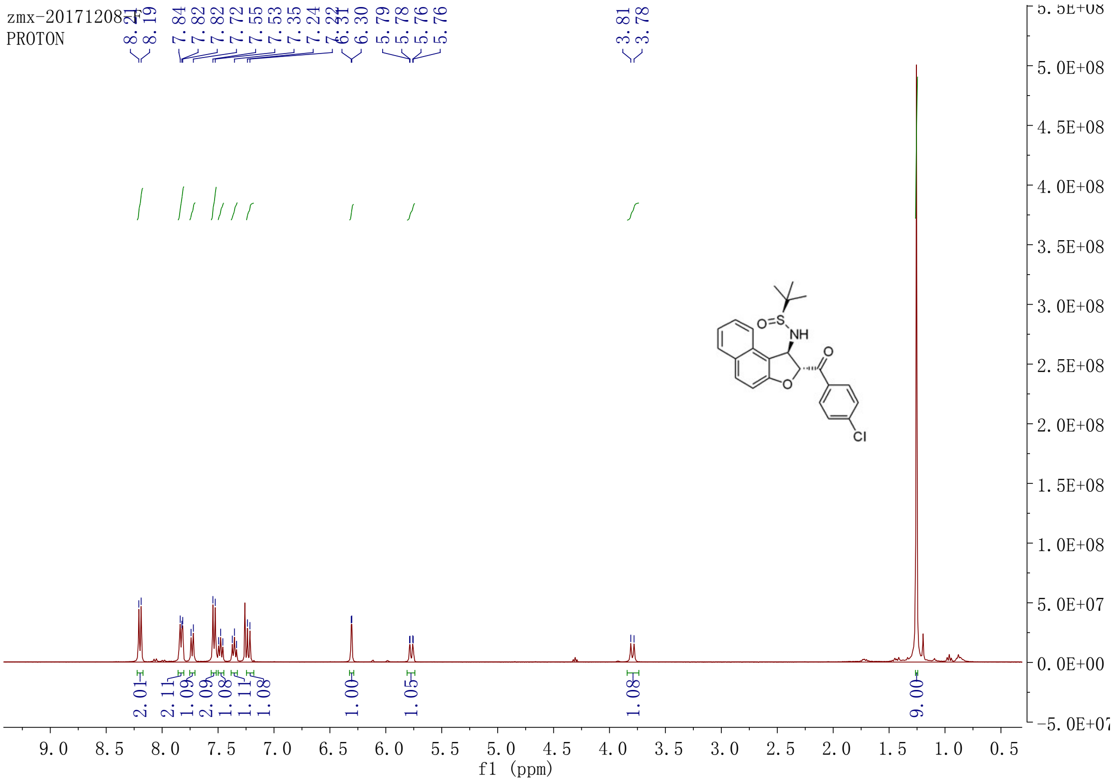

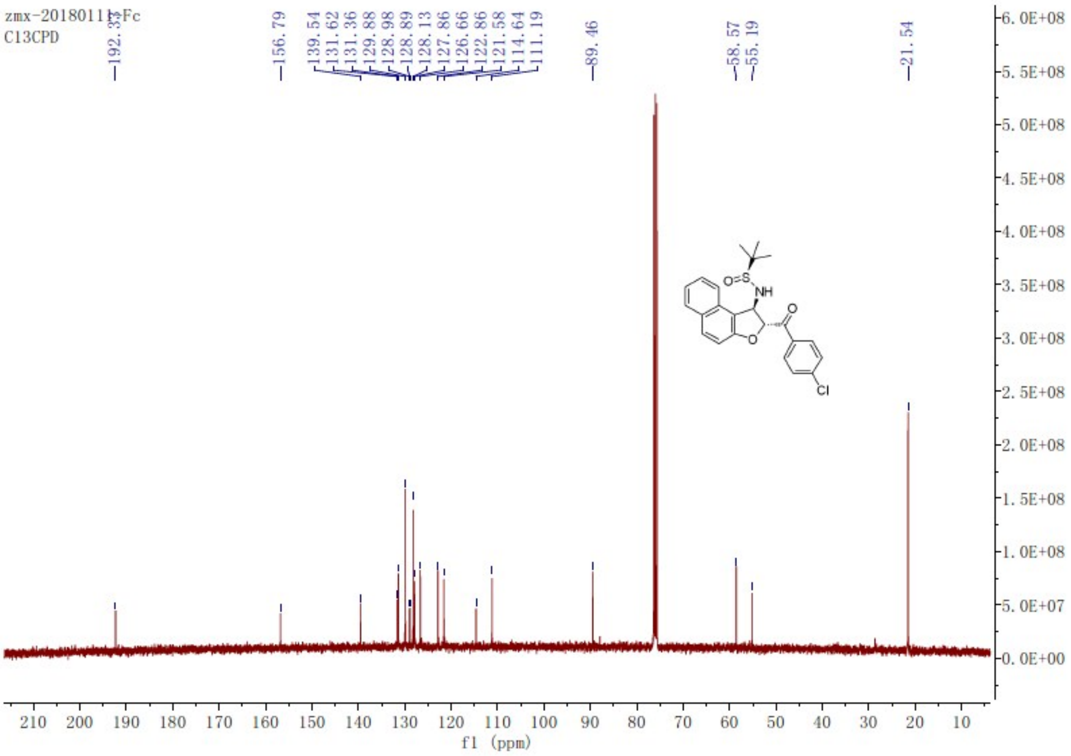

Trans-(S)-2-Methyl-N-((1R,2R)-2-(4-methylbenzoyl)-1,2-dihydronaphtho[2,1-b]furan-1-yl)propane-2-sulfinamide (3j)

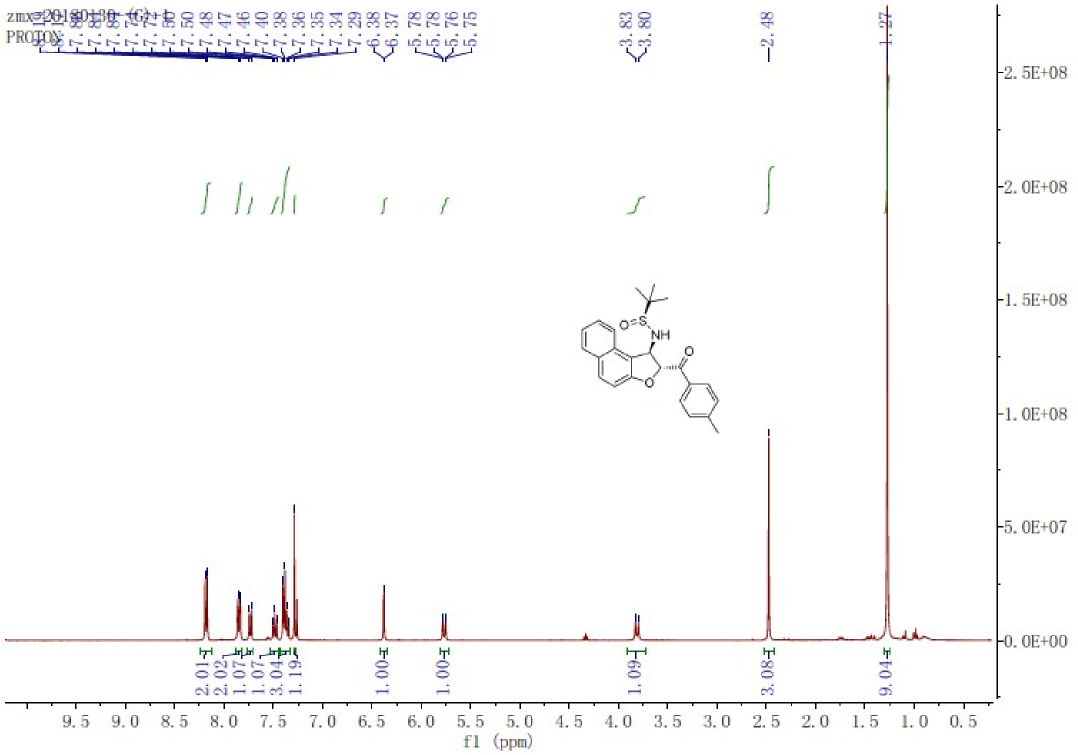

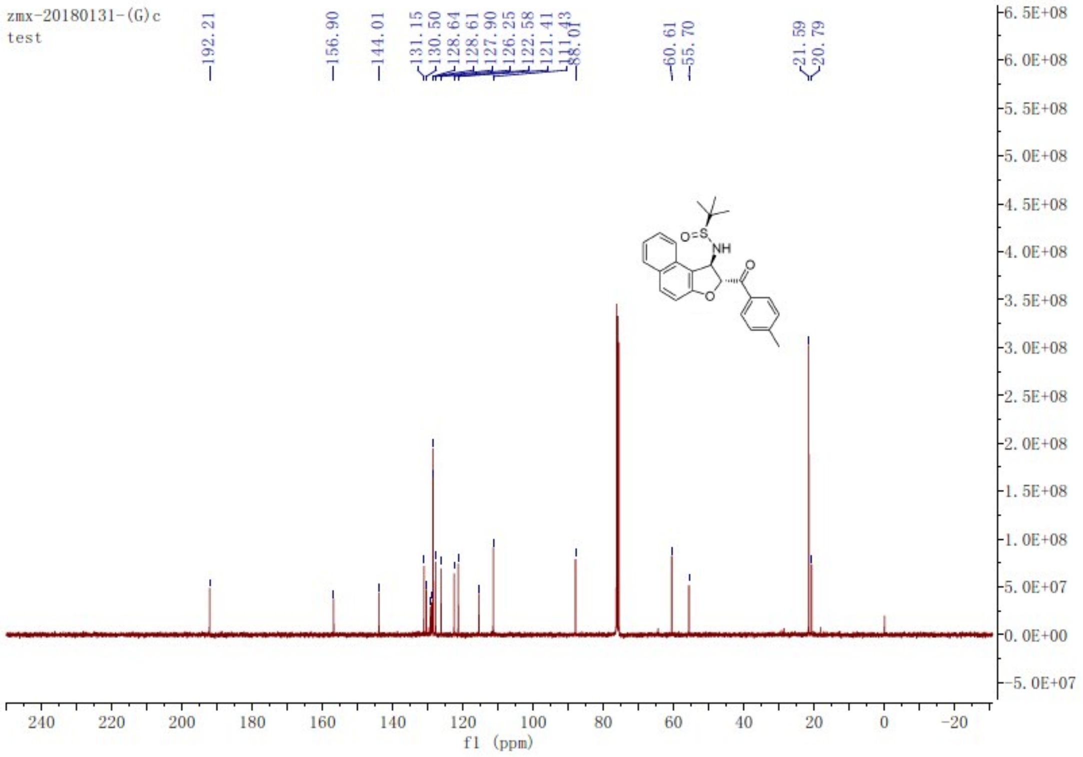

Trans-(S)-N-((2R,3R)-5-Bromo-2-(4-methylbenzoyl)-2,3-dihydrobenzofuran-3-yl)-2-methylpropane-2-sulfinamide (3k)

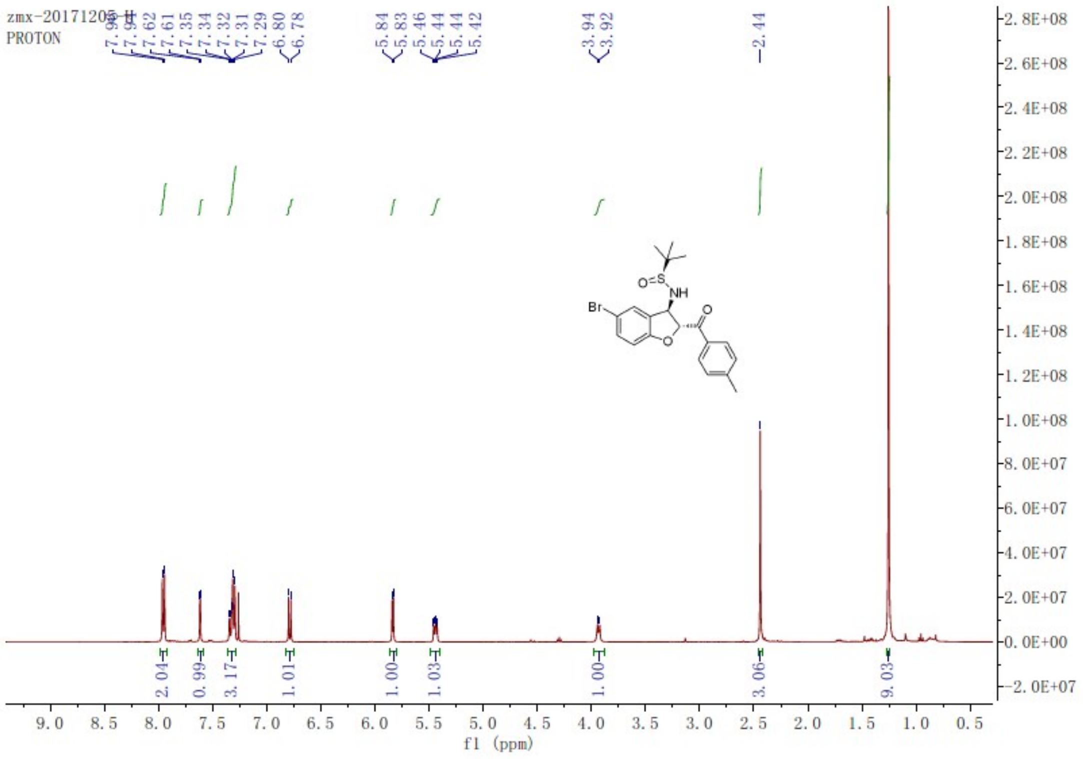

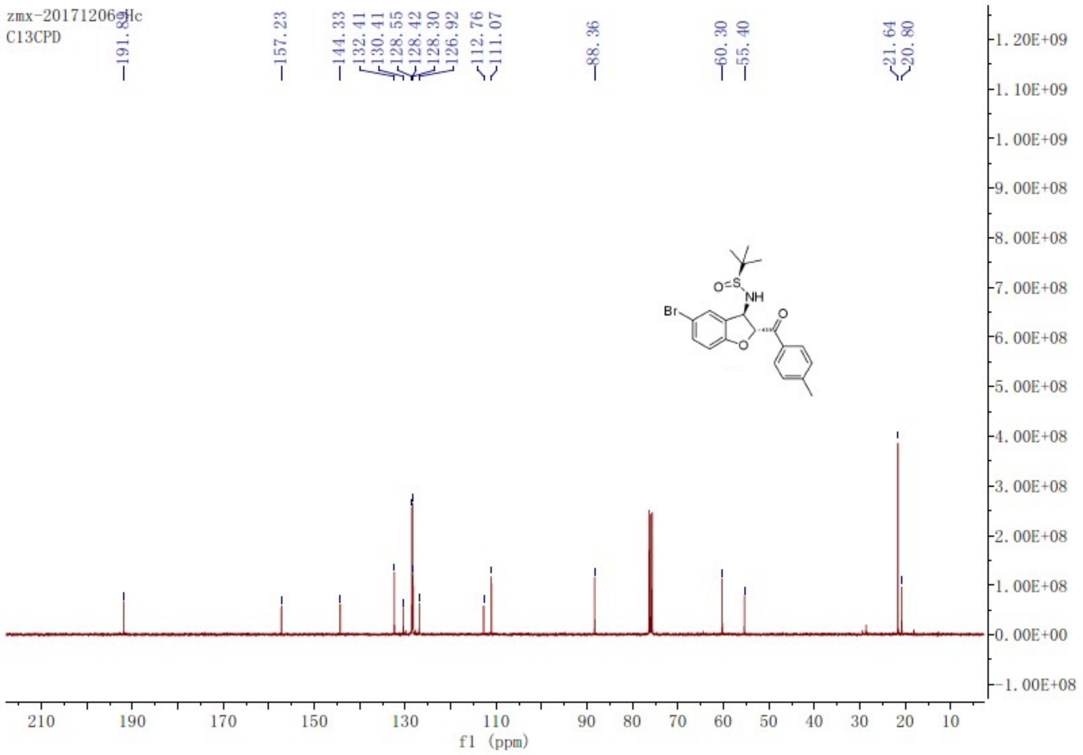

Trans-(S)-N-((2R,3R)-5-Chloro-2-(4-methylbenzoyl)-2,3-dihydrobenzofuran-3-yl)-2-methylpropane-2-sulfinamide (3l)

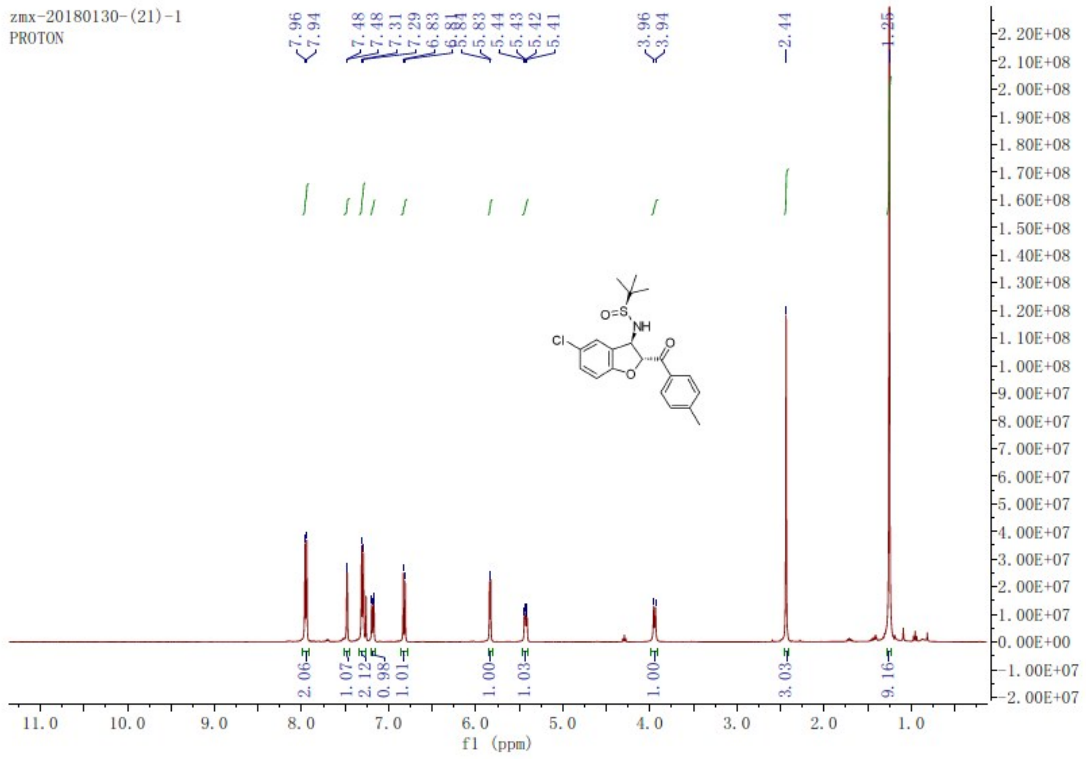

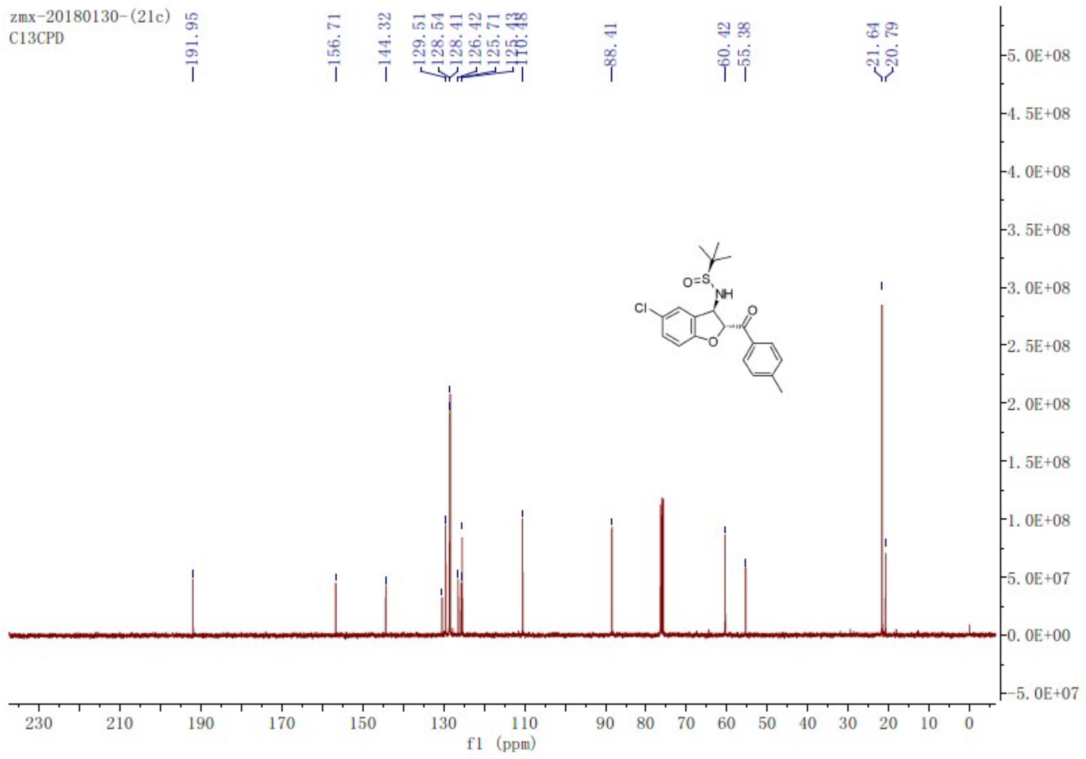

Copies of ^{1}H NMR and ^{13}C NMR spectra of ***(Rs,2S,3S)-trans*-3a-3n**:

Trans-(R)-N-((2S,3S)-2-Benzoyl-2,3-dihydrobenzofuran-3-yl)-2-methylpropane-2-sulfinamide (3a)

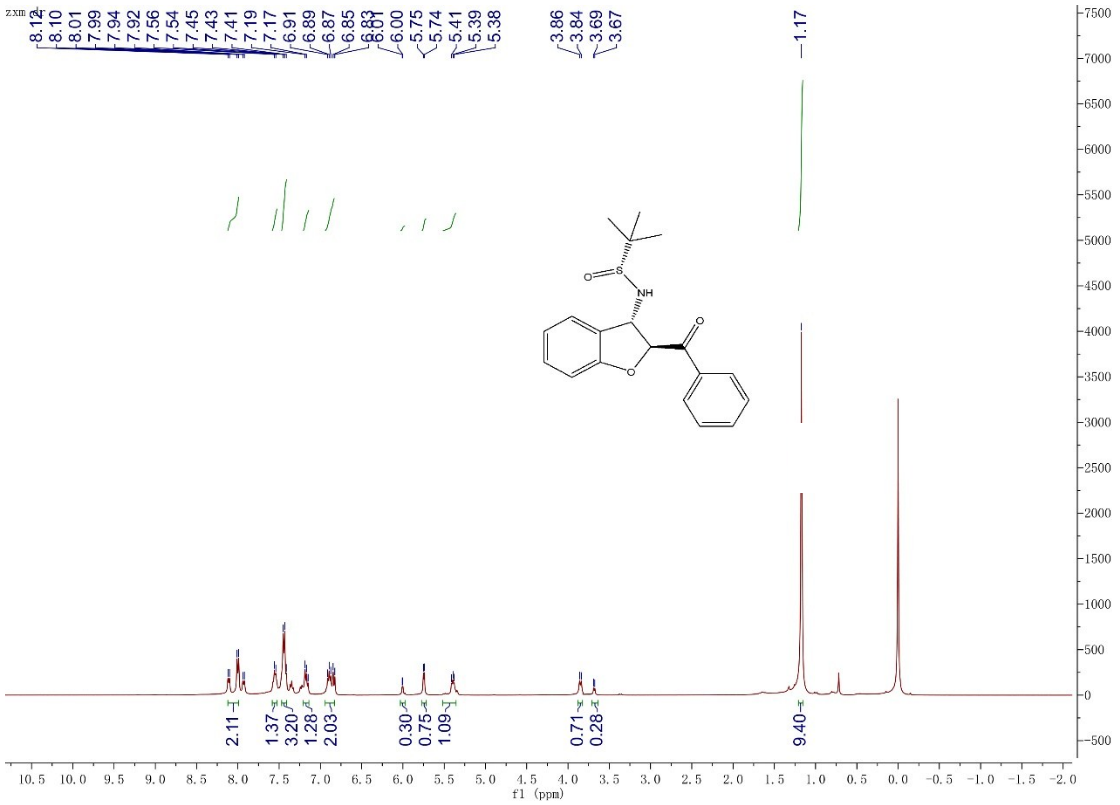

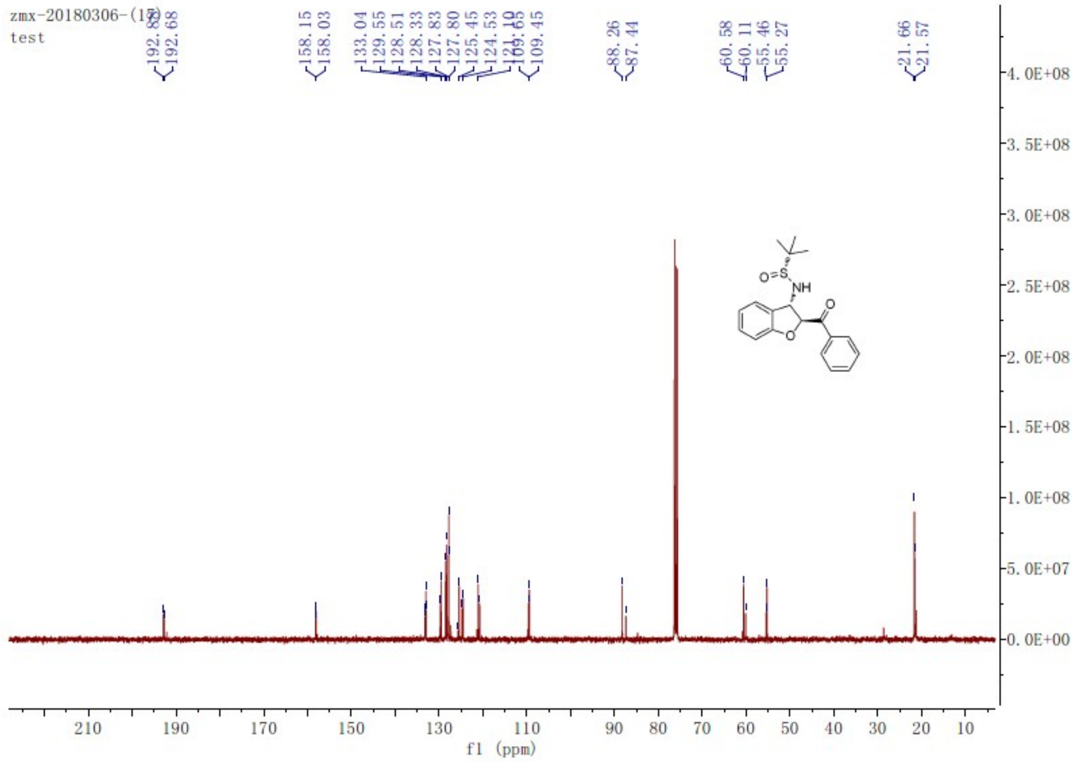

Trans-(R)-N-((2S,3S)-2-Benzoyl-5-chloro-2,3-dihydrobenzofuran-3-yl)-2-methylpropane-2-sulfinamide (3b)

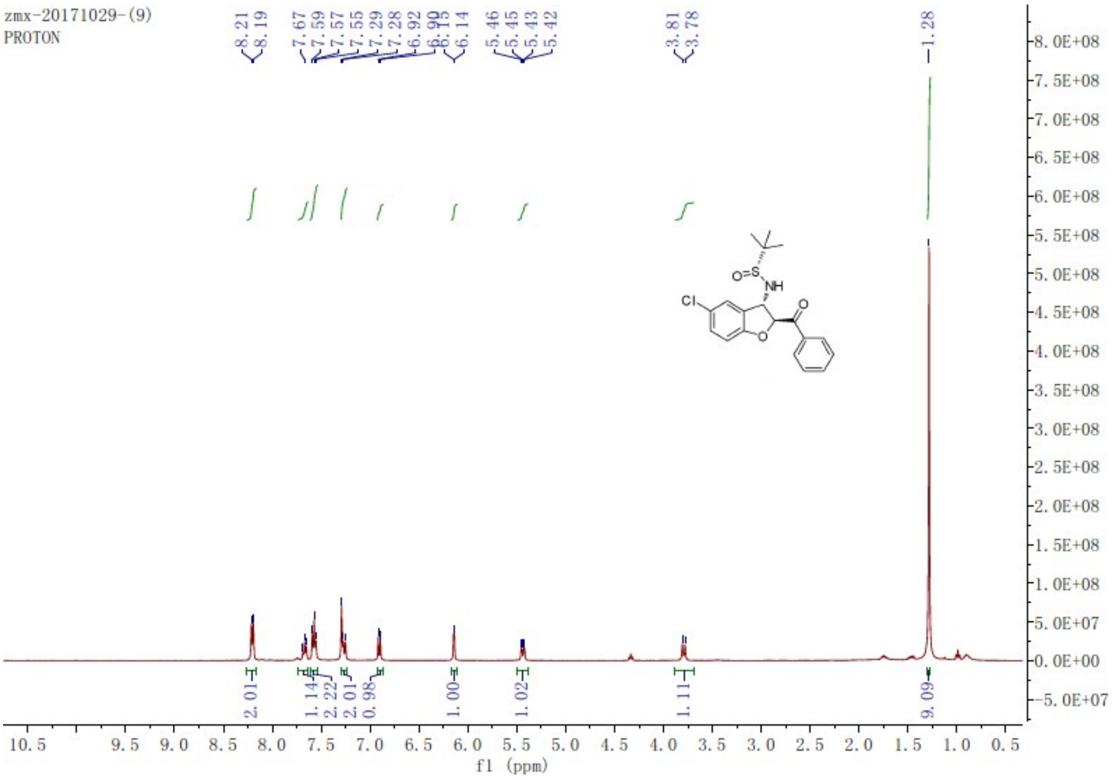

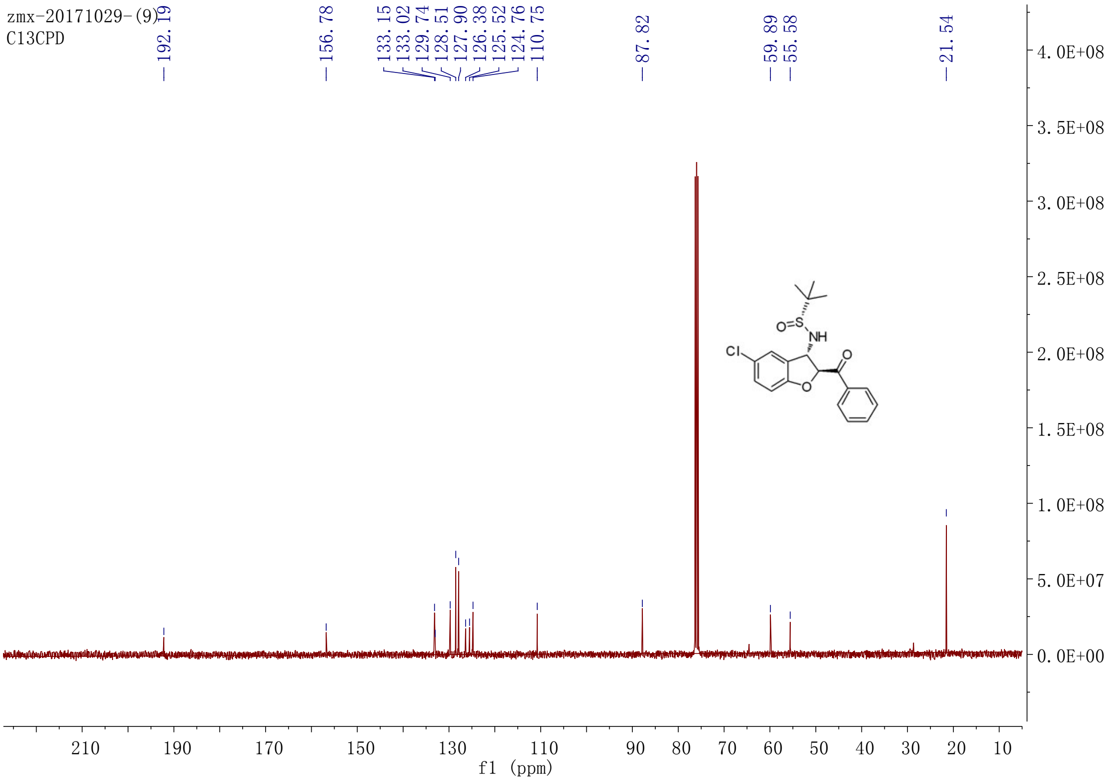

Trans-(R)-N-((2S,3S)-2-Benzoyl-5-bromo-2,3-dihydrobenzofuran-3-yl)-2-methylpropane-2-sulfinamide (3c)

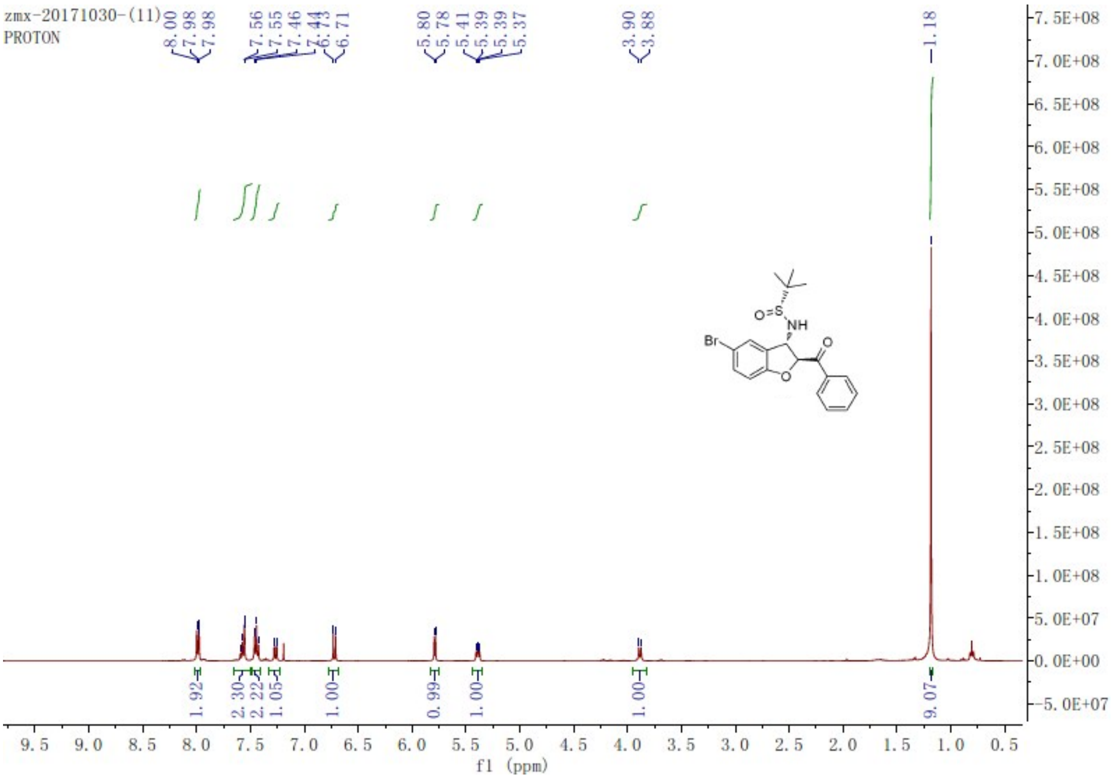

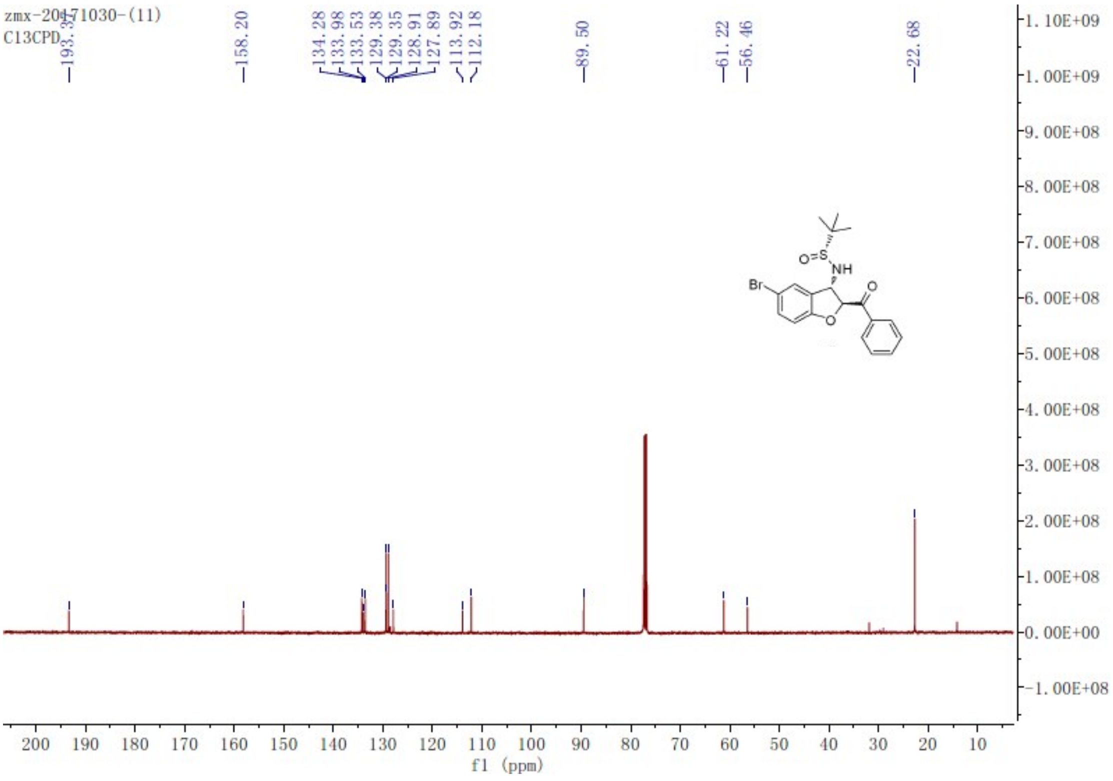

Trans-(R)-N-((2S,3S)-2-Benzoyl-5,7-dichloro-2,3-dihydrobenzofuran-3-yl)-2-methylpropane-2-sulfinamide (3d)

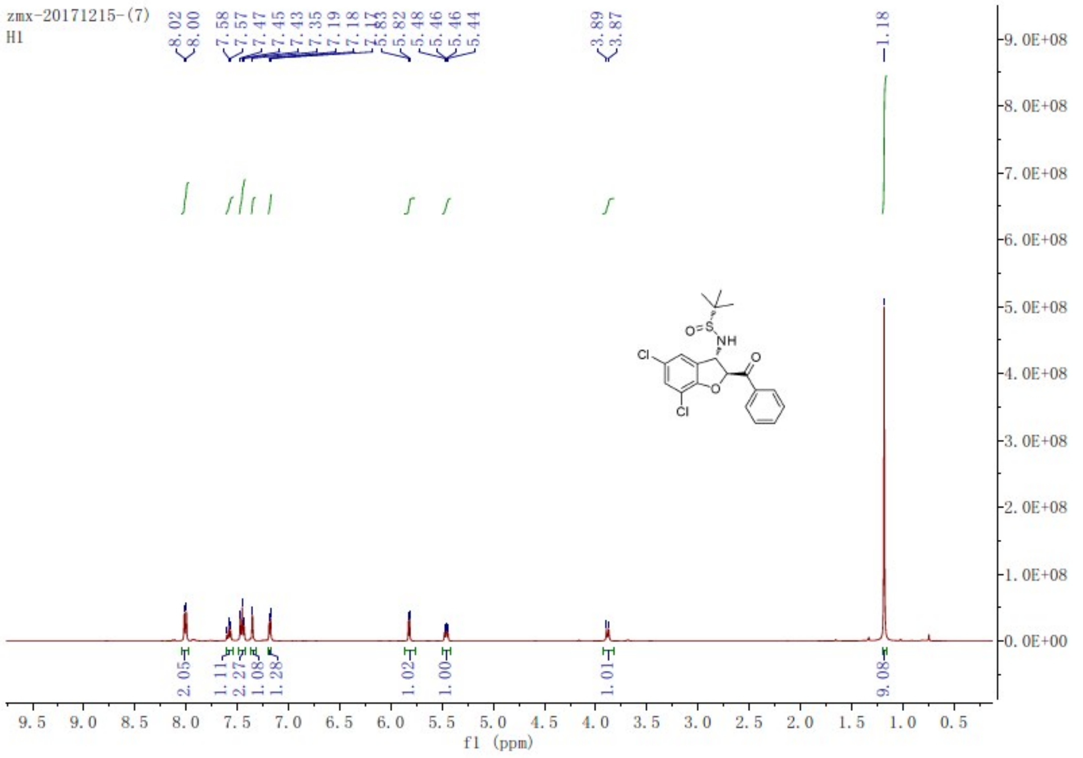

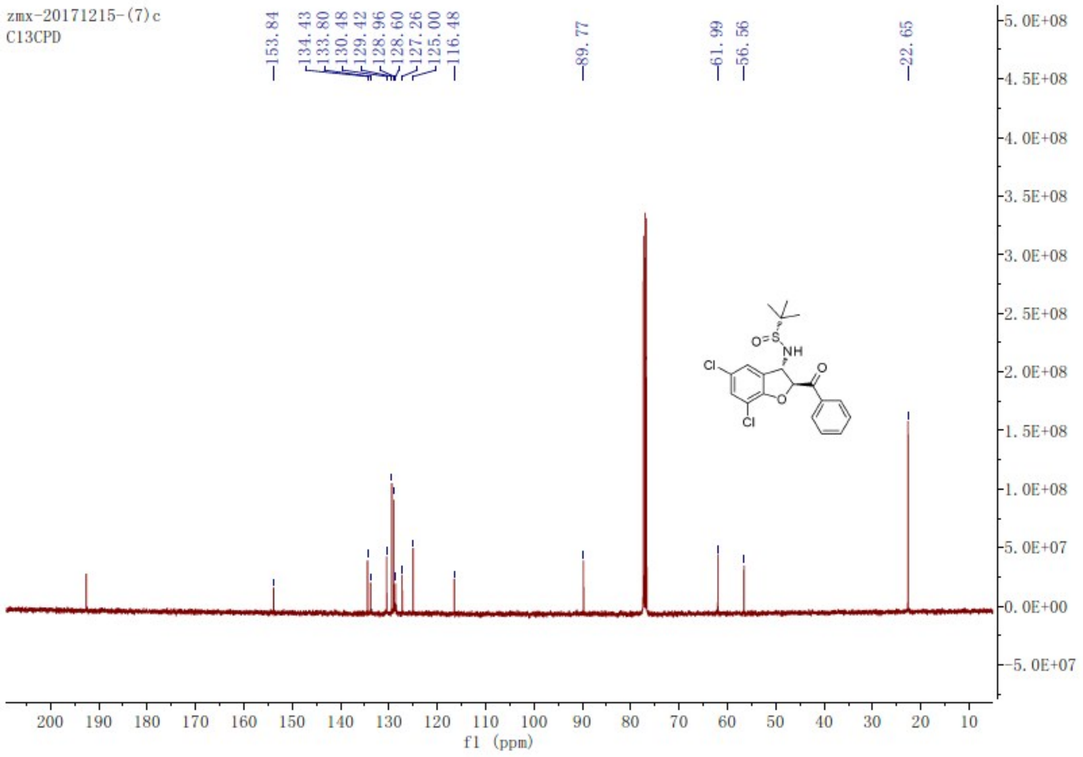

Trans-(R)-N-((1S,2S)-2-benzoyl-1,2-dihydronaphtho[2,1-b]furan-1-yl)-2-methylpropane-2-sulfinamide (3e)

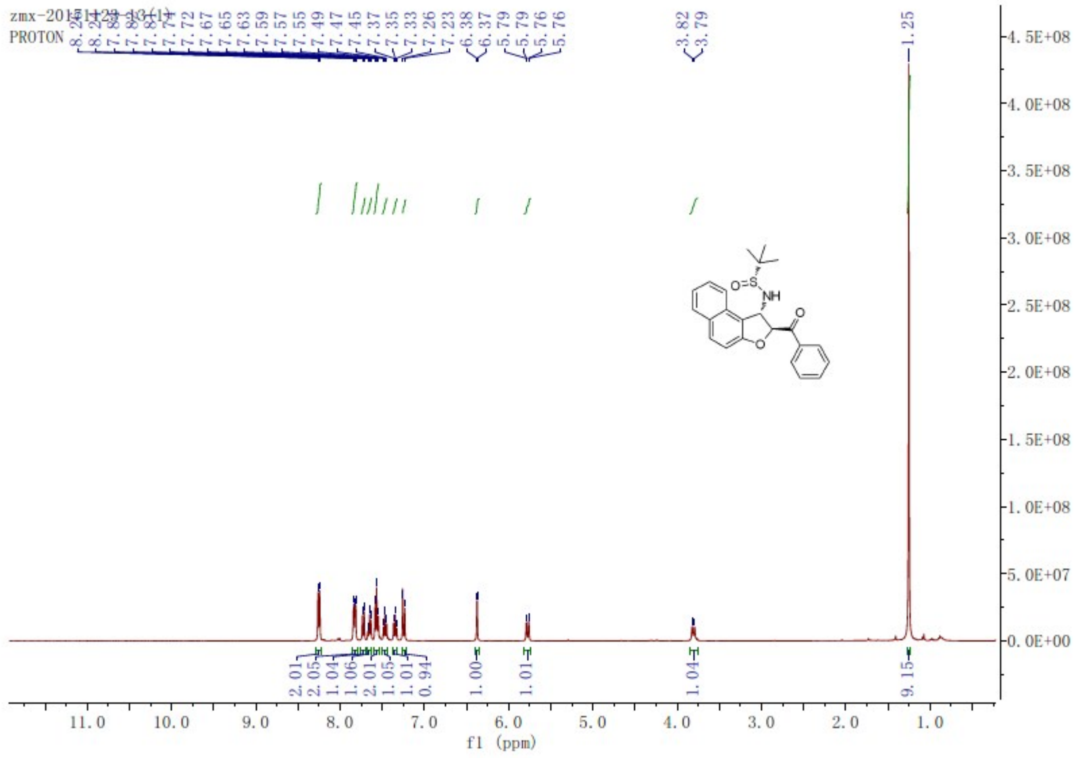

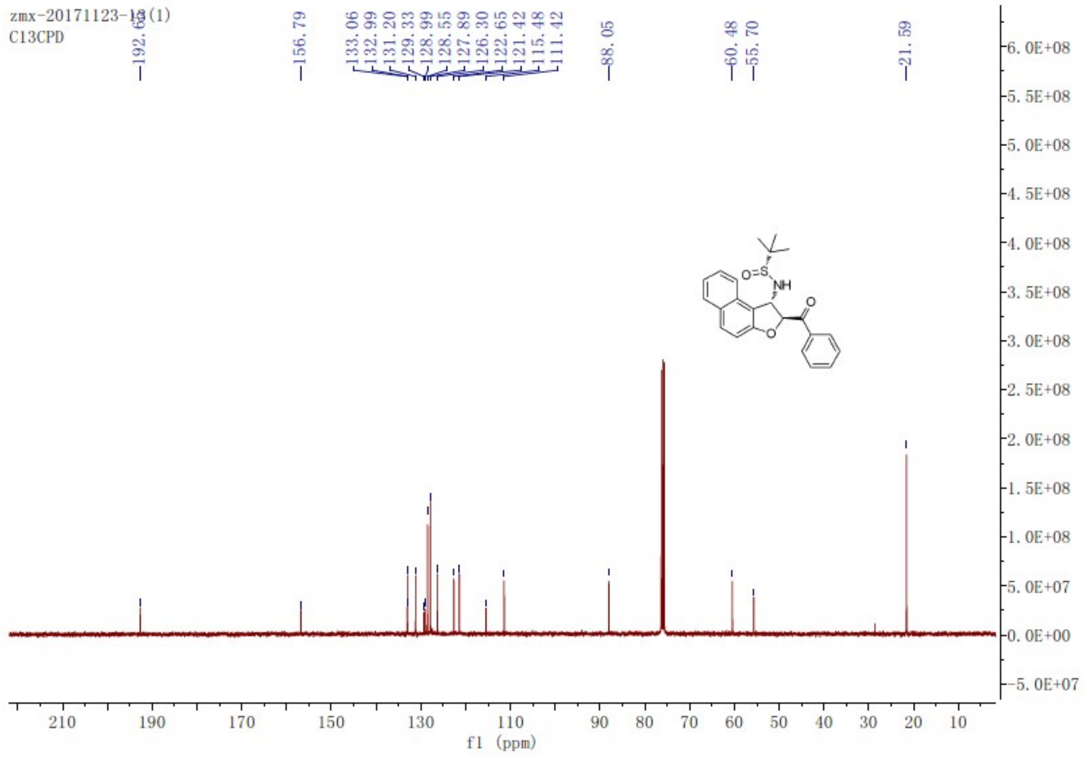

Trans-(R)-N-((2S,3S)-5-chloro-2-(4-chlorobenzoyl)-2,3-dihydrobenzofuran-3-yl)-2-methylpropane-2-sulfinamide (3f)

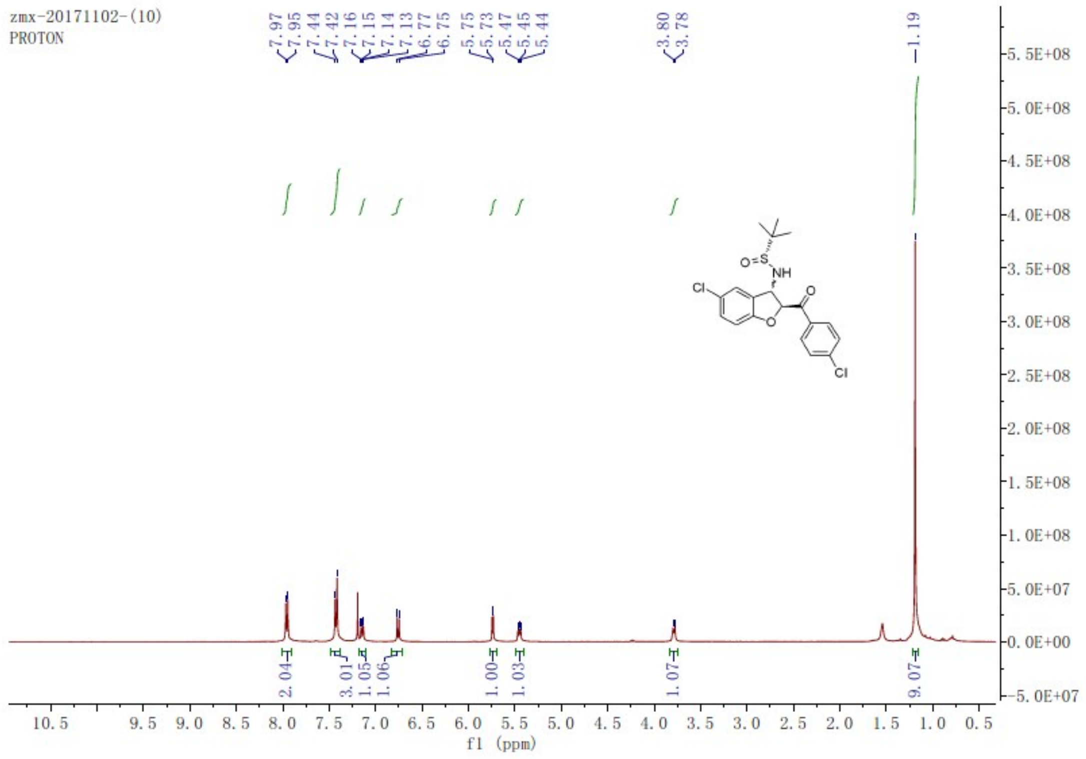

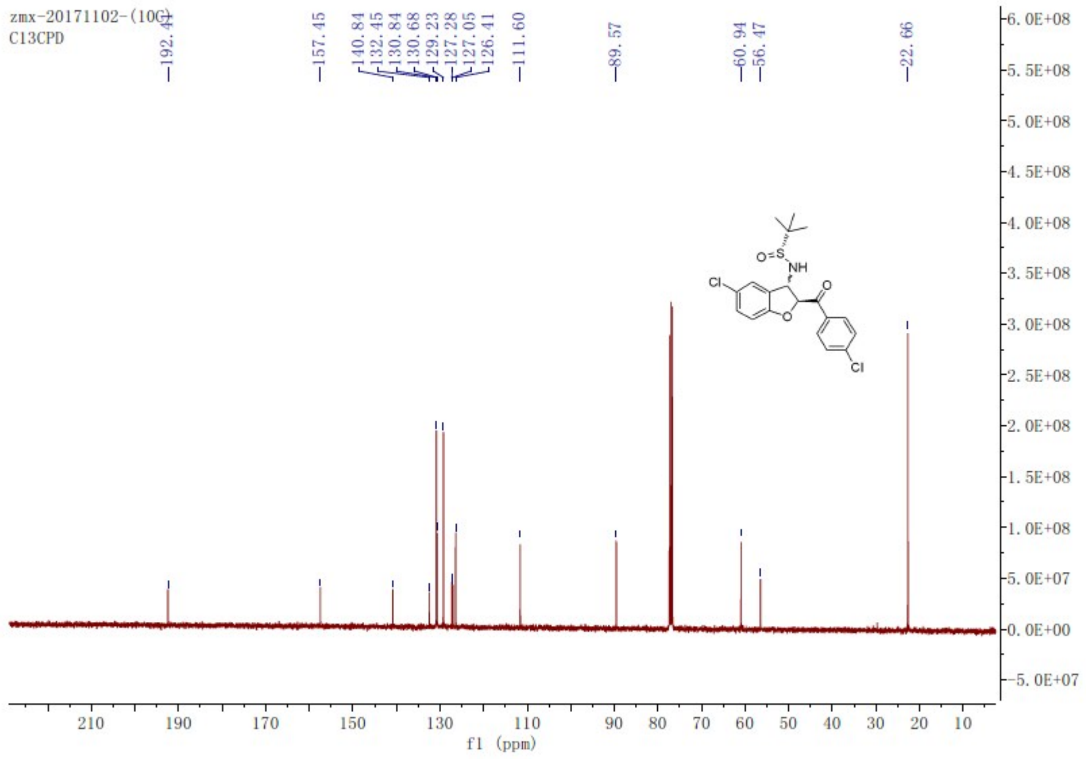

Trans-(R)-N-((2S,3S)-5-Bromo-2-(4-chlorobenzoyl)-2,3-dihydrobenzofuran-3-yl)-2-methylpropane-2-sulfinamide (3g)

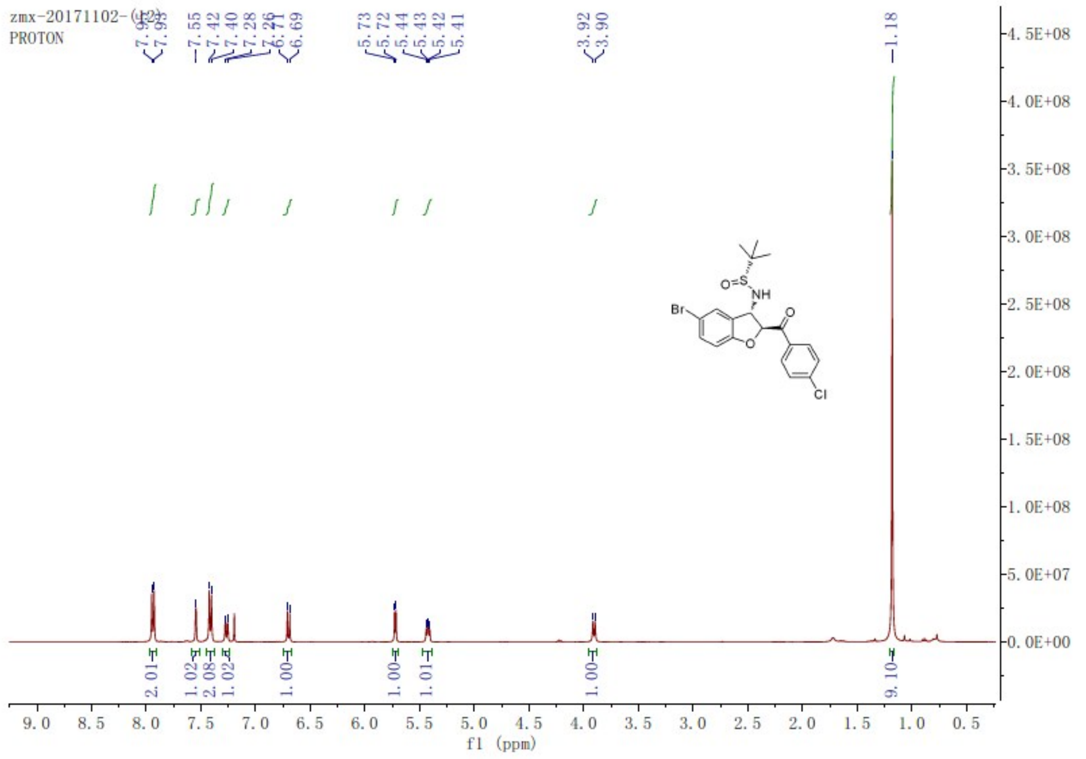

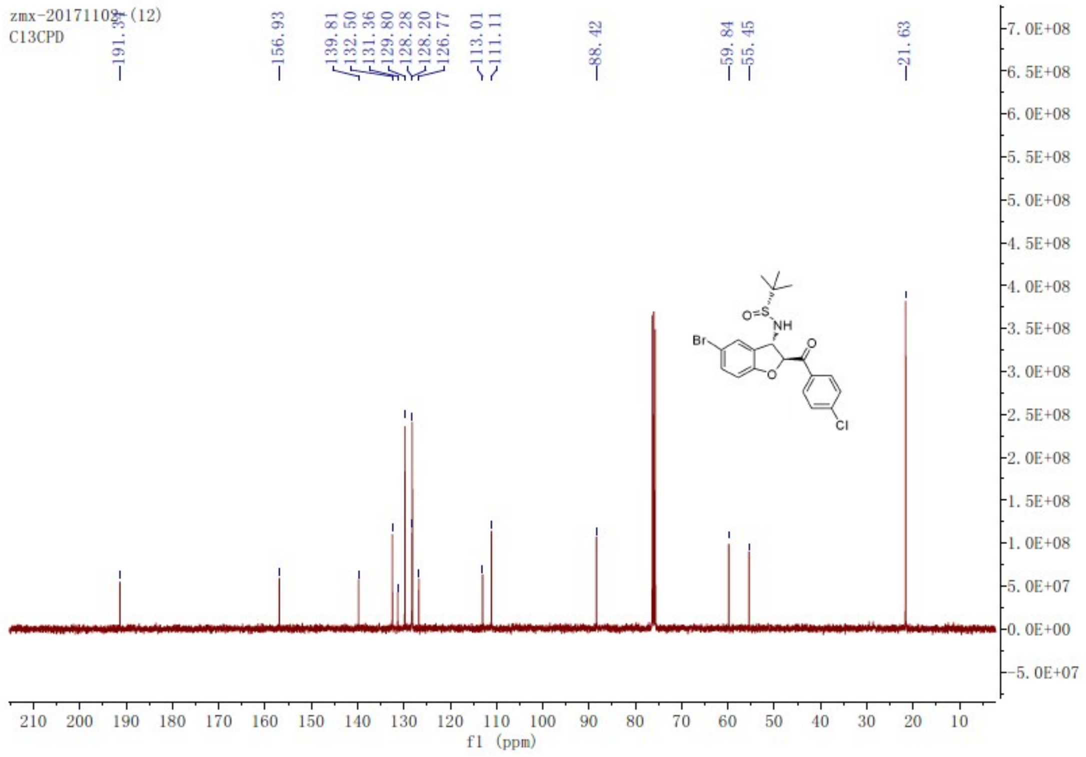

Trans-(R)-N-((2S,3S)-5,7-Dichloro-2-(4-chlorobenzoyl)-2,3-dihydrobenzofuran-3-yl)-2-methylpropane-2-sulfinamide (3h)

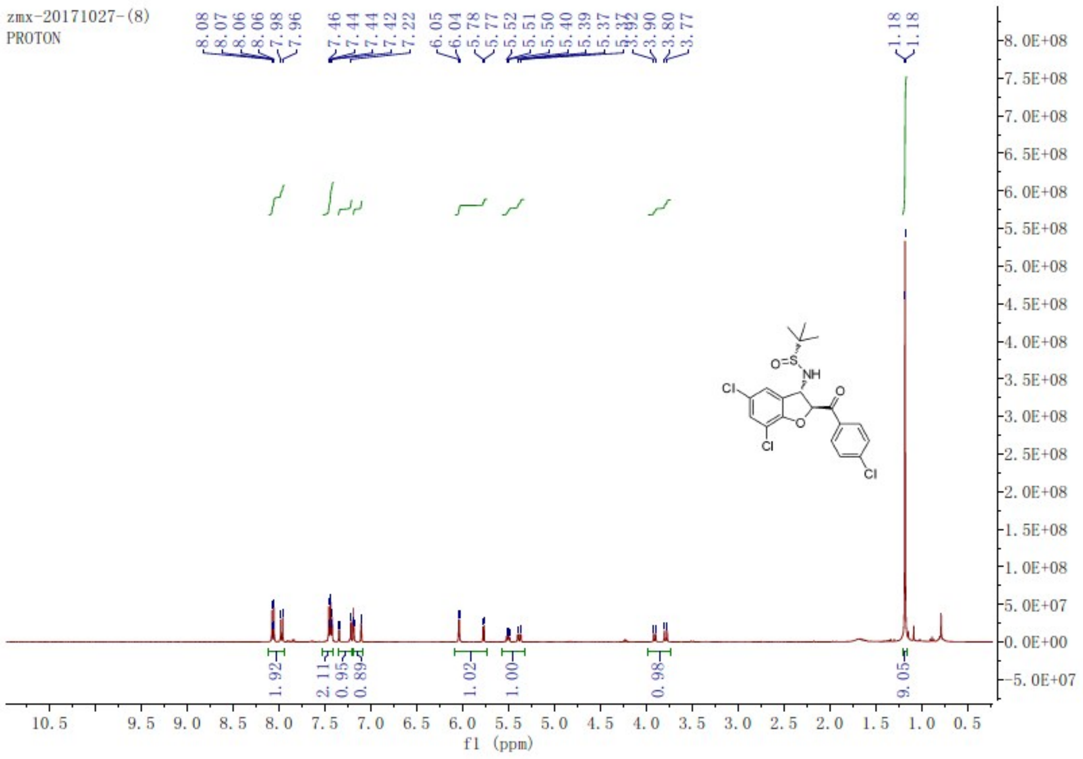

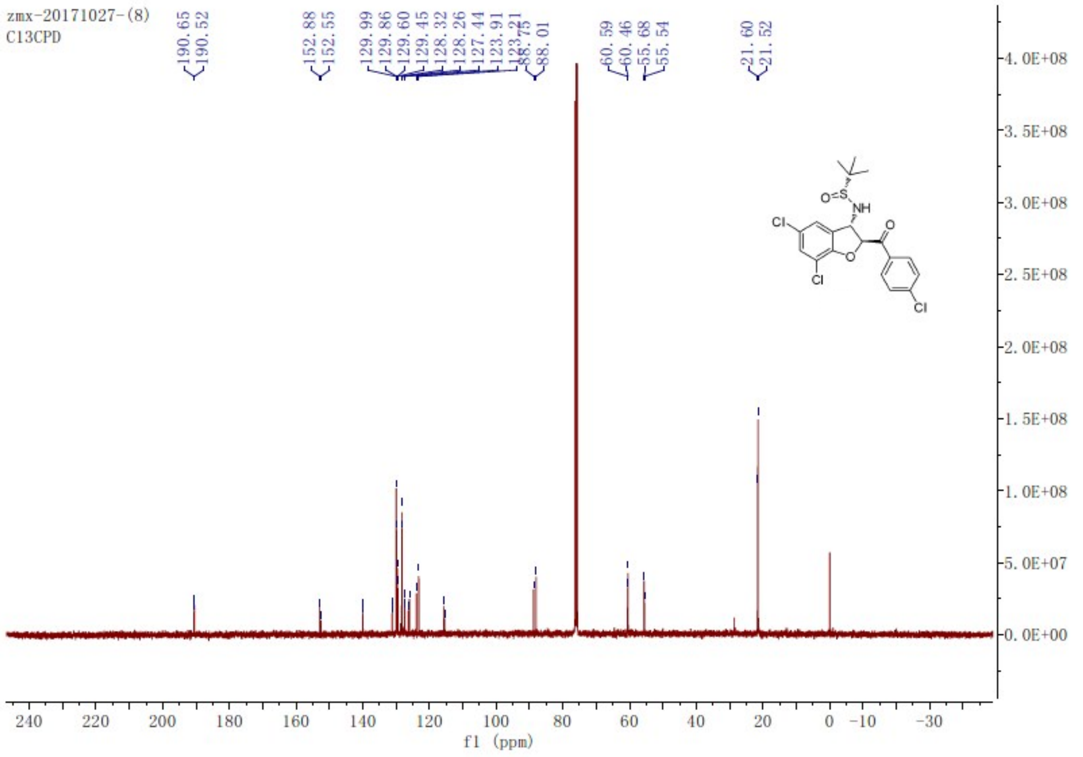

Trans-(R)-N-((1S,2S)-2-(4-Chlorobenzoyl)-1,2-dihydronaphtho[2,1-b]furan-1-yl)-2-methylpropane-2-sulfinamide (3i)

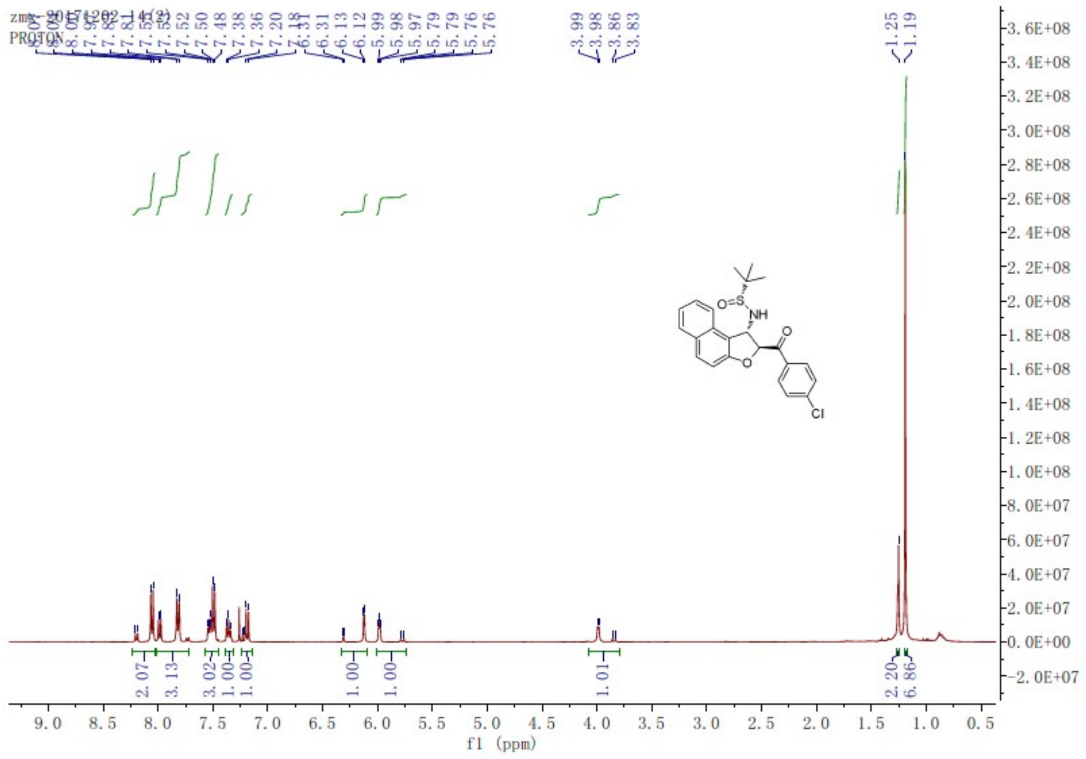

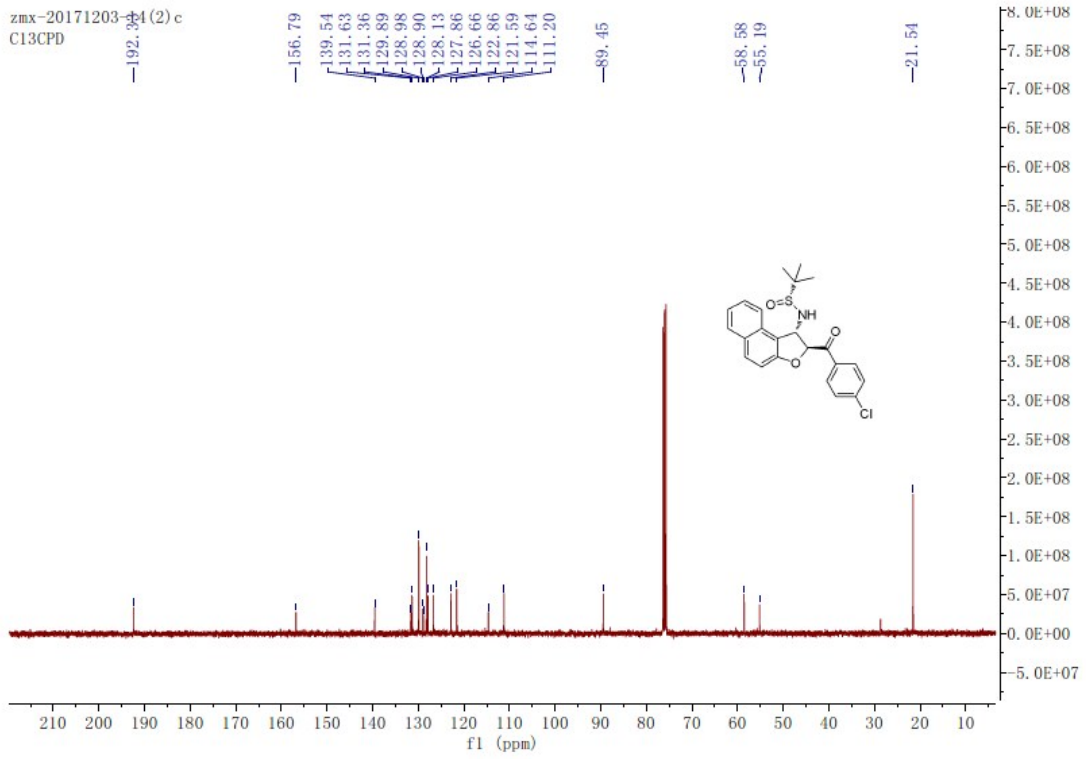

Trans-(R)-2-Methyl-N-((1S,2S)-2-(4-methylbenzoyl)-1,2-dihydronaphtho[2,1-b]furan-1-yl)propane-2-sulfinamide (3j)

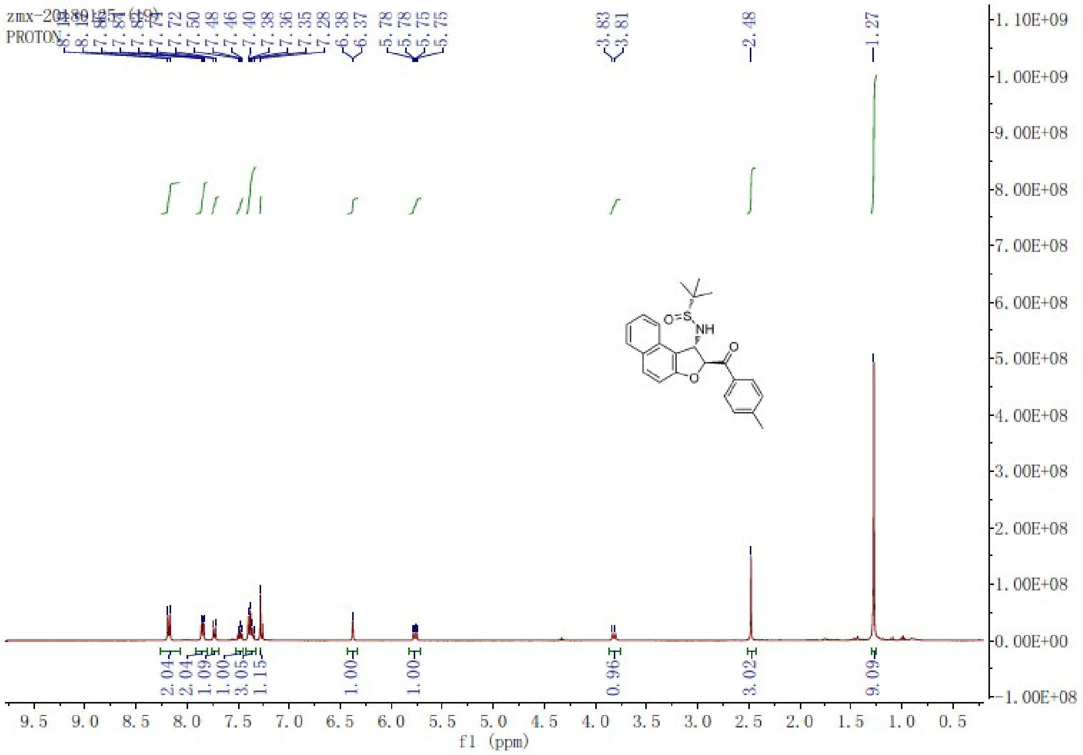

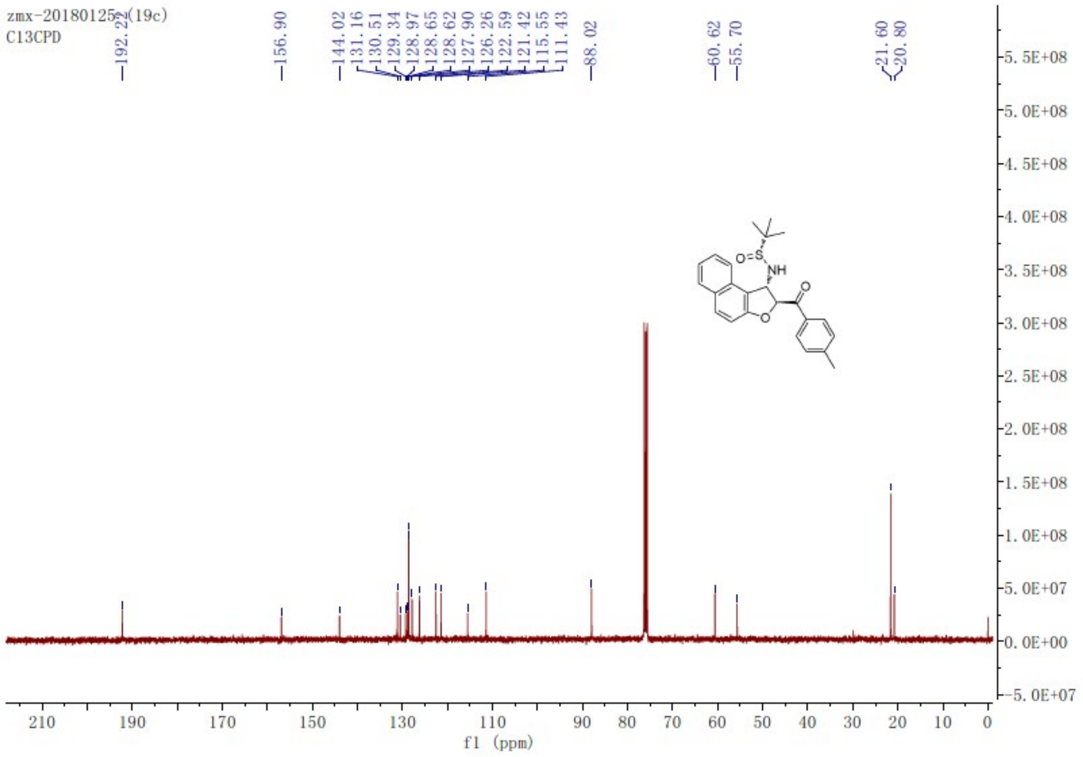

Trans-(R)-N-((2S,3S)-5-Chloro-2-(4-methylbenzoyl)-2,3-dihydrobenzofuran-3-yl)-2-methylpropane-2-sulfinamide (3l)

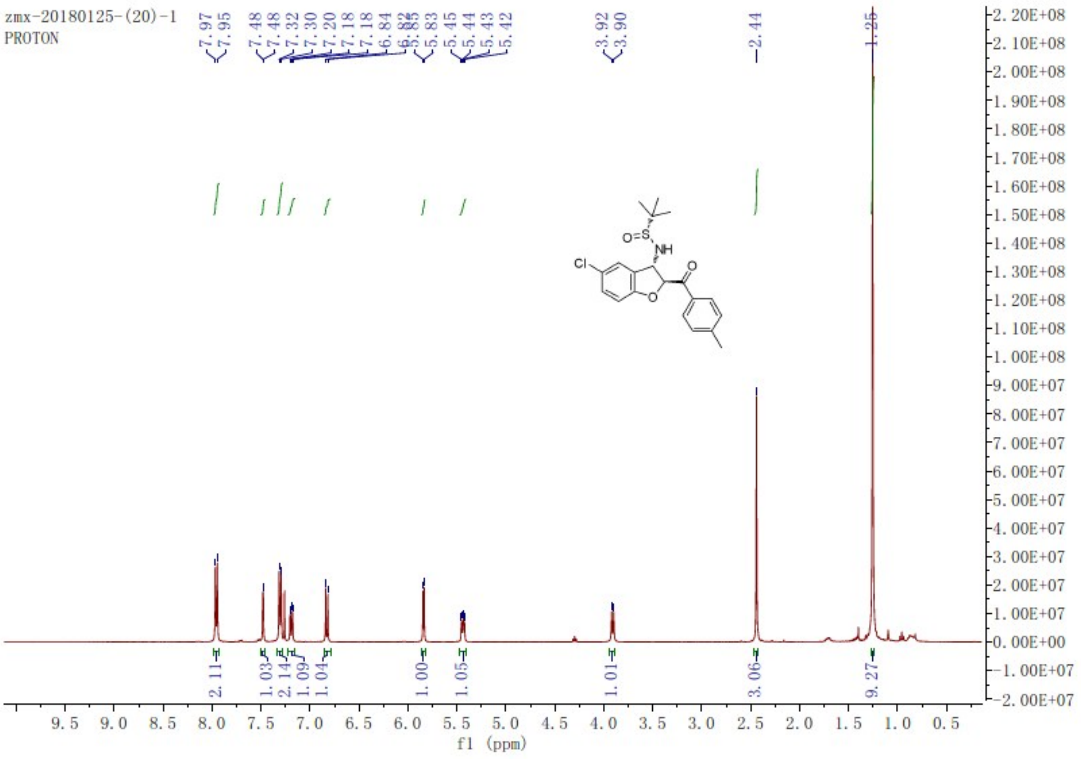

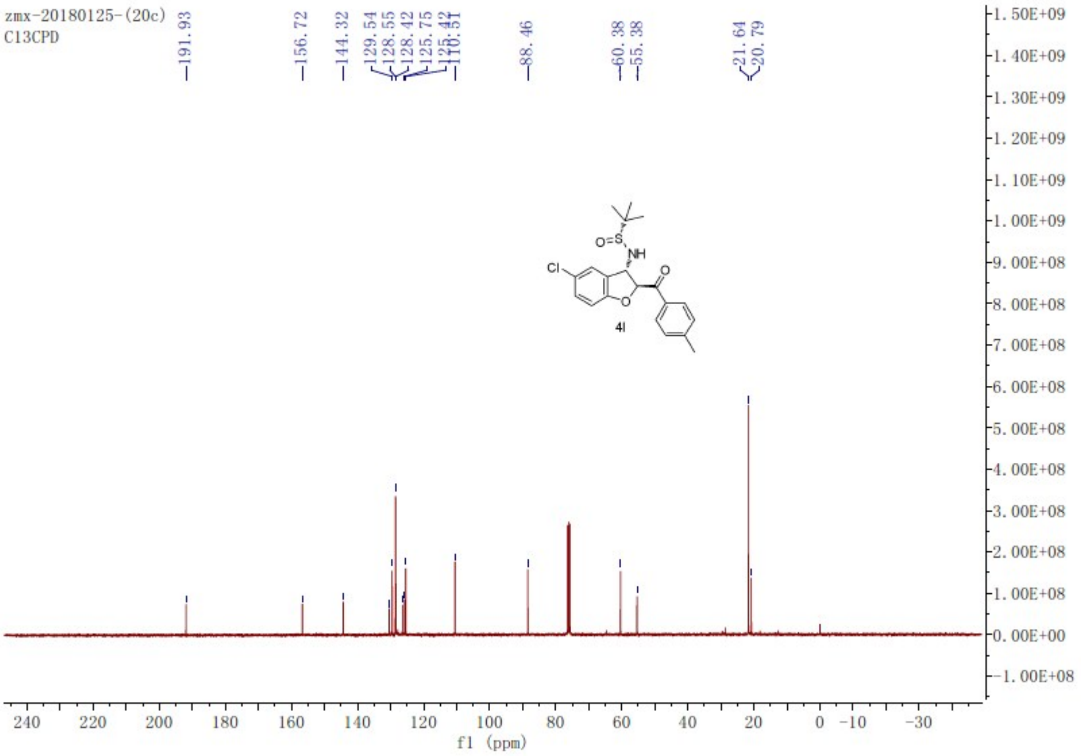

Trans-(R)-N-((2S,3S)-5-Bromo-2-(4-methylbenzoyl)-2,3-dihydrobenzofuran-3-yl)-2-methylpropane-2-sulfinamide (3n)

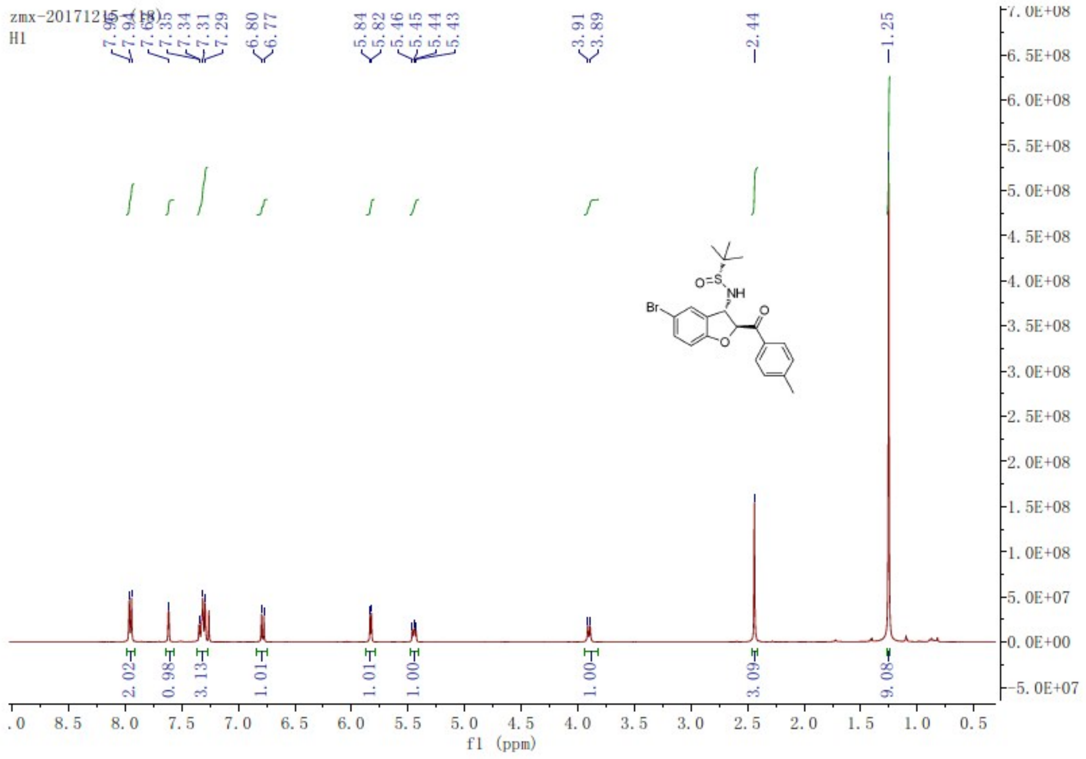

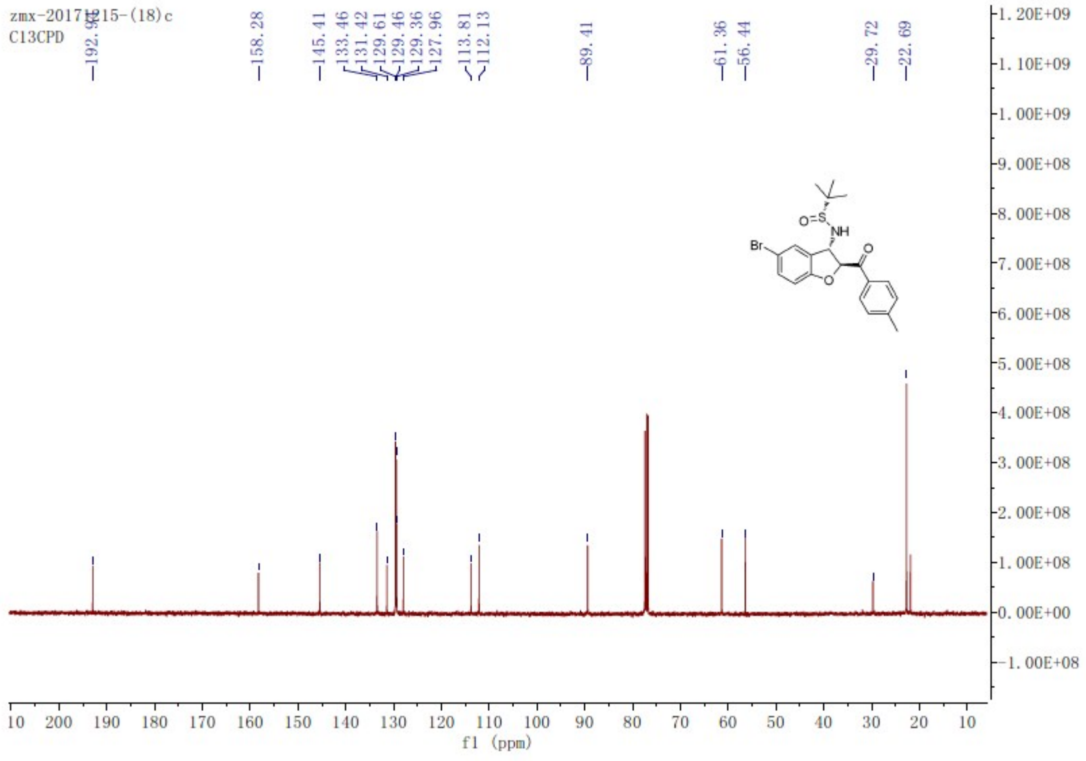

Copies of ^{1}H NMR and ^{13}C NMR spectra of ***cis-(Ss,2R,3R)*-3d**:

Cis-(S)-N-((2S,3R)-2-Benzoyl-5,7-dichloro-2,3-dihydrobenzofuran-3-yl)-2-methylpropane-2-sulfinamide (3d)

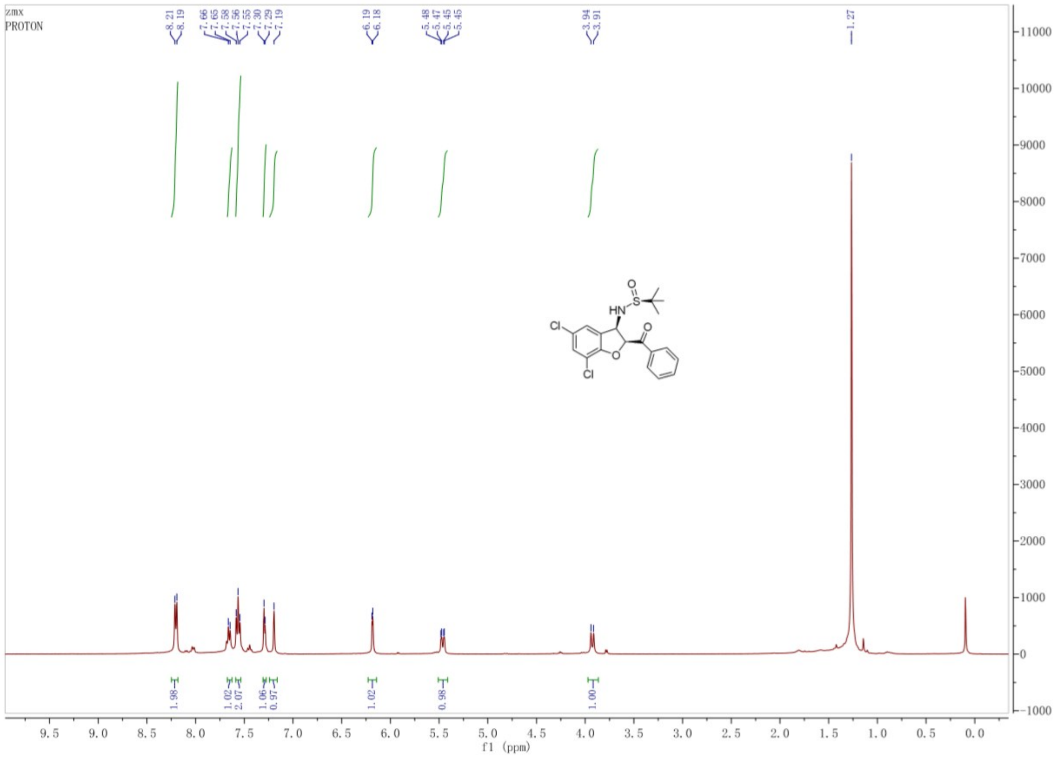

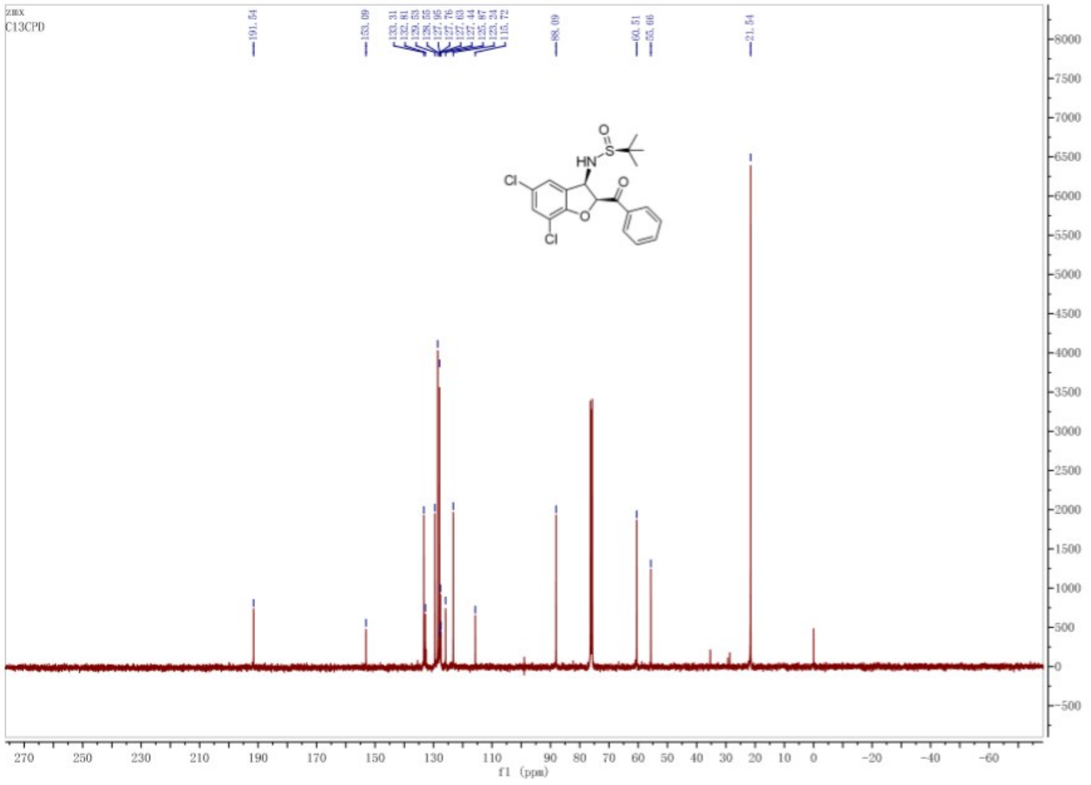

((2R,3R)-3-amino-2,3-dihydrobenzofuran-2-yl)(phenyl)methanone hydrochloride (4a)

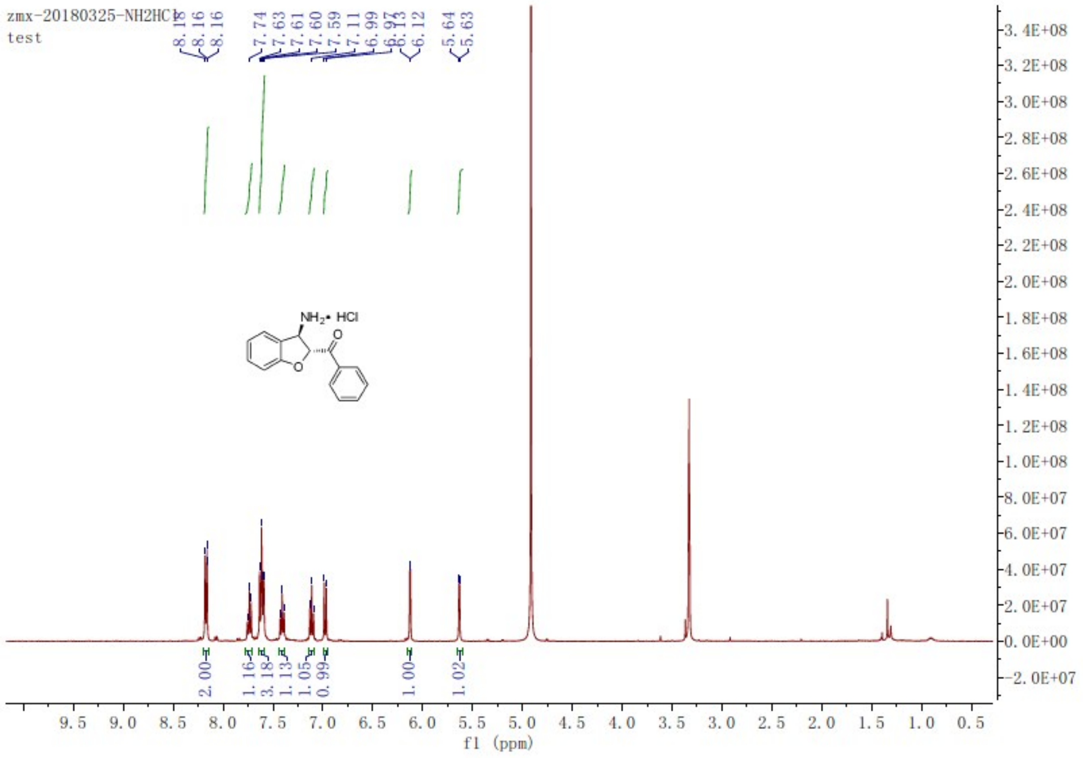

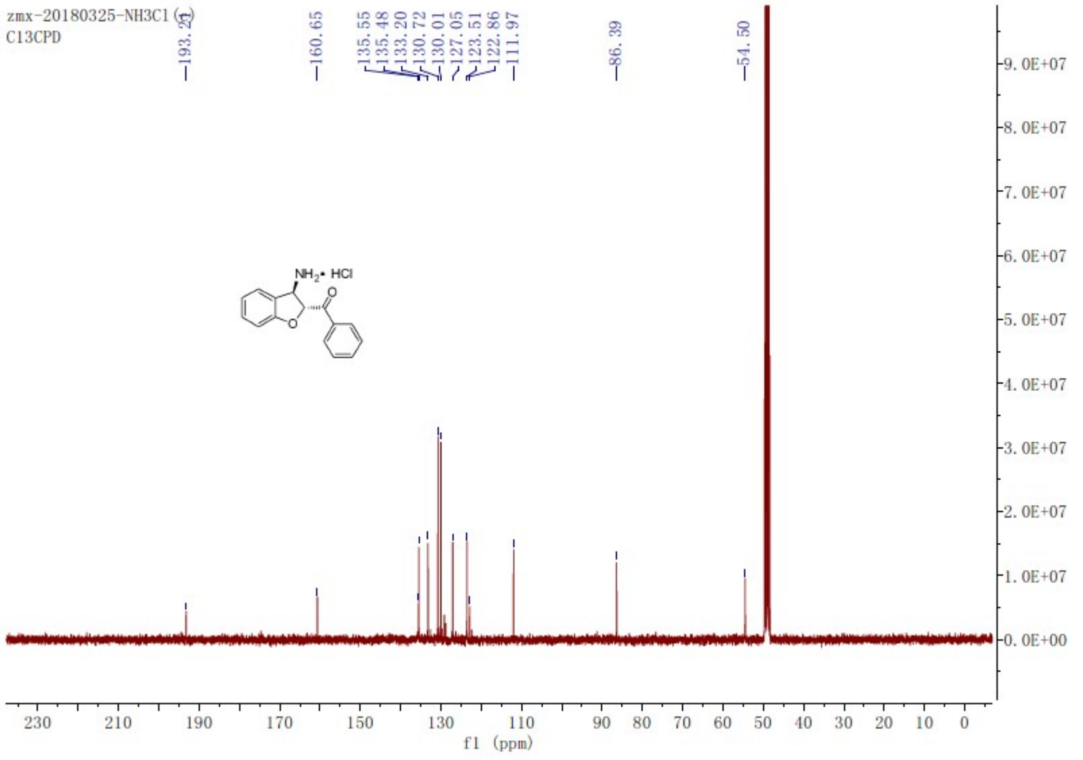

X-ray crystallographic data of compound *trans-(Ss,2R,3R)*-3a

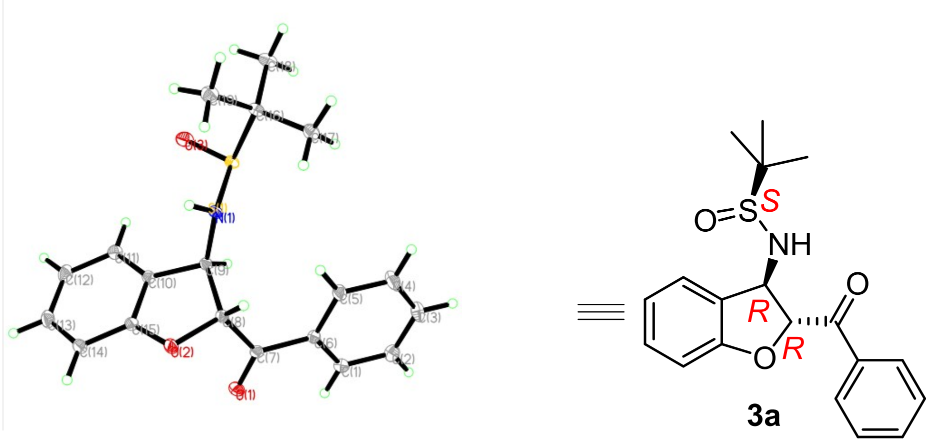

Figure S1 X-ray crystal structure of ***trans*-3a**

Displacement ellipsoids are shown at the 50% probability level.

Table S1 Crystal data and structure refinement for compound ***trans*-3a**

Name	Compound 193e
Empirical formula	$C_{19}H_{21}NO_3S$
Formula weight	343.43
Temperature/K	133.15
Crystal system	orthorhombic
Space group	$P2_12_12_1$
a/Å	9.1621(2)
b/Å	11.5165(2)
c/Å	17.1645(4)
α/°	90.00
β/°	90.00
γ/°	90.00
Volume/Å^3	1811.10(7)
Z	4
ρ_{calc}g/cm^3	1.260

μ/mm^{-1}	0.195
F(000)	728.0
Crystal size/mm^3	0.2 × 0.18 × 0.12
Radiation	MoKα (λ = 0.71073)
2Θ range for data collection/°	4.26 to 61.8
Index ranges	-12 ≤ h ≤ 12, -15 ≤ k ≤ 15, -24 ≤ l ≤ 23
Reflections collected	19767
Independent reflections	5059 [R_{int} = 0.0240, R_{sigma} = 0.0238]
Data/restraints/parameters	5059/1/223
Goodness-of-fit on F^2	1.047
Final R indexes [I>=2σ (I)]	R_1 = 0.0281, wR_2 = 0.0701
Final R indexes [all data]	R_1 = 0.0303, wR_2 = 0.0710
Largest diff. peak/hole / e Å^{-3}	0.21/-0.22
Flack parameter	-0.04(4)

X-ray crystallographic data of compound *trans-(Rs,2S,3S)*-3d

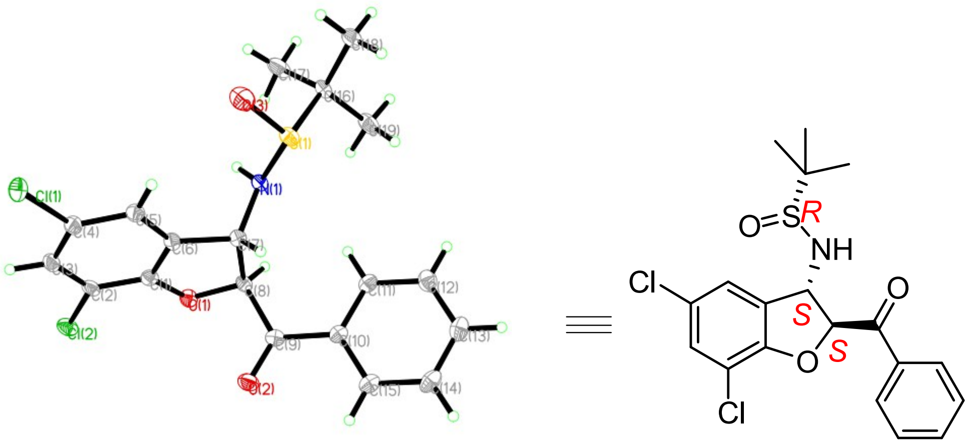

4c

Figure S2 X-ray crystal structure of ***trans*-3d**

Displacement ellipsoids are shown at the 50% probability level.

Table S2 Crystal data and structure refinement for compound ***trans*-3d**

Name	Compound 193c
Empirical formula	$C_{19}H_{19}Cl_2NO_3S$
Formula weight	412.31
Temperature/K	133.15
Crystal system	orthorhombic
Space group	$P2_12_12_1$
a/Å	8.4226(17)
b/Å	14.002(3)
c/Å	16.760(3)
α/°	90.00
β/°	90.00
γ/°	90.00
Volume/Å^3	1976.6(7)

Z	4
ρ_{calc}g/cm^3	1.386
μ/mm^{-1}	0.452
F(000)	856.0
Crystal size/mm^3	0.2 × 0.18 × 0.12
Radiation	MoKα (λ = 0.71073)
2Θ range for data collection/°	4.86 to 55.688
Index ranges	-10 ≤ h ≤ 11, -18 ≤ k ≤ 18, -22 ≤ l ≤ 22
Reflections collected	19834
Independent reflections	4700 [R_{int} = 0.0510, R_{sigma} = 0.0460]
Data/restraints/parameters	4700/1/243
Goodness-of-fit on F^2	0.993
Final R indexes [I>=2σ (I)]	R_1 = 0.0382, wR_2 = 0.0769
Final R indexes [all data]	R_1 = 0.0451, wR_2 = 0.0808
Largest diff. peak/hole / e Å^{-3}	0.33/-0.23
Flack parameter	-0.09(3)
